# A plasmid locus associated with *Klebsiella* clinical infections encodes a microbiome-dependent gut fitness factor

**DOI:** 10.1371/journal.ppat.1009537

**Published:** 2021-04-30

**Authors:** Jay Vornhagen, Christine M. Bassis, Srividya Ramakrishnan, Robert Hein, Sophia Mason, Yehudit Bergman, Nicole Sunshine, Yunfan Fan, Caitlyn L. Holmes, Winston Timp, Michael C. Schatz, Vincent B. Young, Patricia J. Simner, Michael A. Bachman

**Affiliations:** 1 Department of Pathology, University of Michigan, Ann Arbor, MI, United States of America; 2 Department of Microbiology & Immunology, University of Michigan, Ann Arbor, MI, United States of America; 3 Department of Internal Medicine/Infectious Diseases Division, University of Michigan, Ann Arbor, MI, United States of America; 4 Department of Computer Science, Johns Hopkins University, Baltimore, MD, United States of America; 5 Division of Medical Microbiology, Department of Pathology, Johns Hopkins University School of Medicine, Baltimore, MD, United States of America; 6 Department of Biomedical Engineering, Johns Hopkins University, Baltimore, MD, United States of America; 7 Department of Molecular Biology and Genetics, Johns Hopkins University School of Medicine, Baltimore, MD, United States of America; 8 Department of Medicine, Division of Infectious Disease, Johns Hopkins University School of Medicine, Baltimore, MD, United States of America; 9 Department of Biology, Johns Hopkins University, Baltimore, MD, United States of America; 10 Simons Center for Quantitative Biology, Cold Spring Harbor, NY, United States of America; Tufts University, UNITED STATES

## Abstract

*Klebsiella pneumoniae* (Kp) is an important cause of healthcare-associated infections, which increases patient morbidity, mortality, and hospitalization costs. Gut colonization by Kp is consistently associated with subsequent Kp disease, and patients are predominantly infected with their colonizing strain. Our previous comparative genomics study, between disease-causing and asymptomatically colonizing Kp isolates, identified a plasmid-encoded tellurite (TeO_3_^-2^)-resistance (*ter*) operon as strongly associated with infection. However, TeO_3_^-2^ is extremely rare and toxic to humans. Thus, we used a multidisciplinary approach to determine the biological link between *ter* and Kp infection. First, we used a genomic and bioinformatic approach to extensively characterize Kp plasmids encoding the *ter* locus. These plasmids displayed substantial variation in plasmid incompatibility type and gene content. Moreover, the *ter* operon was genetically independent of other plasmid-encoded virulence and antibiotic resistance loci, both in our original patient cohort and in a large set (n = 88) of publicly available *ter* operon-encoding Kp plasmids, indicating that the *ter* operon is likely playing a direct, but yet undescribed role in Kp disease. Next, we employed multiple mouse models of infection and colonization to show that 1) the *ter* operon is dispensable during bacteremia, 2) the *ter* operon enhances fitness in the gut, 3) this phenotype is dependent on the colony of origin of mice, and 4) antibiotic disruption of the gut microbiota eliminates the requirement for *ter*. Furthermore, using 16S rRNA gene sequencing, we show that the *ter* operon enhances Kp fitness in the gut in the presence of specific indigenous microbiota, including those predicted to produce short chain fatty acids. Finally, administration of exogenous short-chain fatty acids in our mouse model of colonization was sufficient to reduce fitness of a *ter* mutant. These findings indicate that the *ter* operon, strongly associated with human infection, encodes factors that resist stress induced by the indigenous gut microbiota during colonization. This work represents a substantial advancement in our molecular understanding of Kp pathogenesis and gut colonization, directly relevant to Kp disease in healthcare settings.

## Author summary

The bacterial pathogen *Klebsiella pneumoniae* is of substantial public health concern due to its ability to cause serious antibiotic-resistant infections. These infections frequently occur in healthcare settings, especially in patients with detectable gut colonization by *K*. *pneumoniae*. Importantly, infectious *K*. *pneumoniae* strains are often detected in the gut of patients with *K*. *pneumoniae* disease, indicating that the gut is a reservoir of infectious *K*. *pneumoniae*. Our previous work interrogating the genetic underpinnings of *K*. *pneumoniae* disease in colonized patients identified a strong association between *K*. *pneumoniae* infection and the presence of an enigmatic genetic locus known as the *ter* operon. We found that this operon is not needed for pneumonia and bacteremia, and therefore, we explored the importance of the *ter* operon in the gut. *K*. *pneumoniae* lacking *ter* function was at a disadvantage in the gut, thus explaining the connection between the *ter* operon and infection in hospitalized patients. Interestingly, the advantage conferred by the *ter* operon in the gut was associated with the presence of specific indigenous gut microbiota and the presence of short-chain fatty acids, which are metabolized by the host and gut microbiota. This work demonstrates that the *ter* operon is a microbiome-dependent gut fitness factor and suggests that indigenous gut bacteria may limit colonization by infectious *K*. *pneumoniae*.

## Introduction

The emergence and spread of highly antibiotic-resistant bacteria have substantially complicated disease treatment and control. Enterobacteriaceae are a significant contributor to the burden of antibiotic-resistant infections through the production of extended-spectrum beta-lactamases (ESBL-) and carbapenemases (CP-). Within the Enterobacteriaceae family is *Klebsiella pneumoniae* (Kp), which is a substantial threat to human health, as it is the third leading cause of all hospital-acquired infections [1,2]. Infection with ESBL- and CP-Kp is associated with staggeringly high mortality (>50%) and excessive healthcare costs [3,4], leading the Centers for Disease Control and Prevention to categorize ESBL- and CP-Kp as serious and urgent threats, respectively. Further complicating this issue, strains of hypervirulent Kp (hvKp) have independently emerged in Southeast Asia. HvKp strains cause severe community-acquired infections that are associated with mortality rates as high as 31% [5]. Furthermore, hvKp and antibiotic-resistant strains are reported to be converging, leading to dangerous, highly antibiotic-resistance strains of hvKp, which have recently been detected outside of Southeast Asia [6–9]. As these dangerous strains circulate more widely and as the number of effective treatments dwindles, alternative interventions are necessary to diminish the threat posed by these bacteria.

Gut colonization by Kp is consistently associated with subsequent Kp disease [10–14], and patients are predominantly infected with their colonizing strain [10,11]. However, colonizing strains likely vary in their potential to cause infection. Variation in virulence potential is likely mediated by the presence or absence of genes in the accessory genome of each isolate and may be important at any step between colonization maintenance in the gut to fitness at the eventual site of infection. As gut colonization often precedes infection [11,12], murine models of Kp colonization are needed that are relevant to human infection. Several studies have rapidly advanced our understanding of Kp gut colonization, including the relevance of the indigenous gut microbiota to Kp fitness during colonization [15,16]; however, the factors underlying these interactions remain unexplored. Importantly, previous studies have shown that gut microbiota differs by mouse vendor and by the room within each vendor facility (referred to as “barrier”) [17] and these differences can impact experimental output [18–20]. Therefore, any evaluation of fitness factors during colonization should account for variations in the microbiota and how different indigenous bacteria may interact with Kp directly or indirectly.

Comparing infected to asymptomatically colonized patients, we have previously identified the tellurium resistance operon, known as the *ter* operon, as highly associated with Kp pneumonia and bacteremia (OR = 11.3, 95% CI = 1.6–80.0 after adjustment for clinical variables). This enigmatic operon is found in many diverse bacteria, archaea, and some eukaryotes wherein it bestows resistance to the toxic compound tellurite oxide (TeO_3_^-2^) [21]. The antibacterial property of TeO_3_^-2^ was first described by Sir Alexander Fleming in 1932 [22], and the reduction of TeO_3_^-2^ to Te^0^ in bacterial cells underlying that antimicrobial property was discovered even earlier in 1914 [23]. Resistance to TeO_3_^-2^ has long been used for clinical detection of *Corynebacterium diphtheriae* and other pathogens [23–26]. There are several distinct genetic loci (*ter*, *teh*, *tel*, *kil*, others) involved in TeO_3_^-2^ resistance whose gene products are predicted to be mechanistically divergent [21,27]. Of these, the *ter* operon is least understood. It is highly unlikely that the physiological function of the *ter* operon is to resist TeO_3_^-2^, as this compound is exceedingly rare in the environment, and not present in humans. Previous mechanistic studies of the *ter* operon in non-Kp bacteria suggest a pleiotropic function, with evidence for resistance to oxidative, genotoxic, heavy metal, proton motive force, cell wall, membrane, phage, and protein synthesis stress [21,28,29], as well as a role for intracellular survival in macrophages [30,31]. Moreover, previous studies have suggested that the *ter* operon is transcriptionally regulated by the OxyRS system [29,32], further suggesting a connection between the *ter* operon and stress. Interestingly, this operon is found in several other pathogenic Enterobacterales, such as *Yersinia pestis*, *Proteus mirabilis*, and enterohemorrhagic *Escherichia coli* [29,31,33,34]. The association with infection, and the unique biology of TeO_3_^-2^ and the *ter* operon collectively imply that this operon is spuriously annotated based on historical *in vitro* findings, warranting further investigation into its true physiological function during Kp infection.

In Kp, the *ter* operon is found on pK2044-like plasmids that encode multiple virulence genes characteristic of hypervirulent Kp strains (hvKp) [35,36]. This suggests the association between the presence of the *ter* operon and Kp disease could be due to genetic linkage with plasmid-encoded virulence genes. Moreover, the *ter* operon was identified as a point of recombination for the Kp hypervirulence plasmid and a carbapenemase encoding plasmid [37], suggesting it can both enhance fitness and enable the convergence of two worrying Kp pathotypes. To distinguish linkage with virulence genes from an inherent function of the *ter* operon in pathogenesis, we performed comparative genomic studies on a broad collection of Kp plasmids and assessed fitness of isogenic *ter* mutants of a hypervirulent strain in a model of gut colonization in two distinct microbial communities. Collectively, these data reveal that the *ter* operon, highly associated with human infection, likely acts early in pathogenesis as a horizontally transferrable fitness factor promoting robust gut colonization in the presence of the indigenous microbiota. Analyses of these indigenous microbiota revealed a role for short-chain fatty acid (SCFA) metabolism in the reduction of gut fitness for Kp lacking a functional *ter* operon.

## Results

### Characterization of the Kp *ter* operon

**Fig 1:**
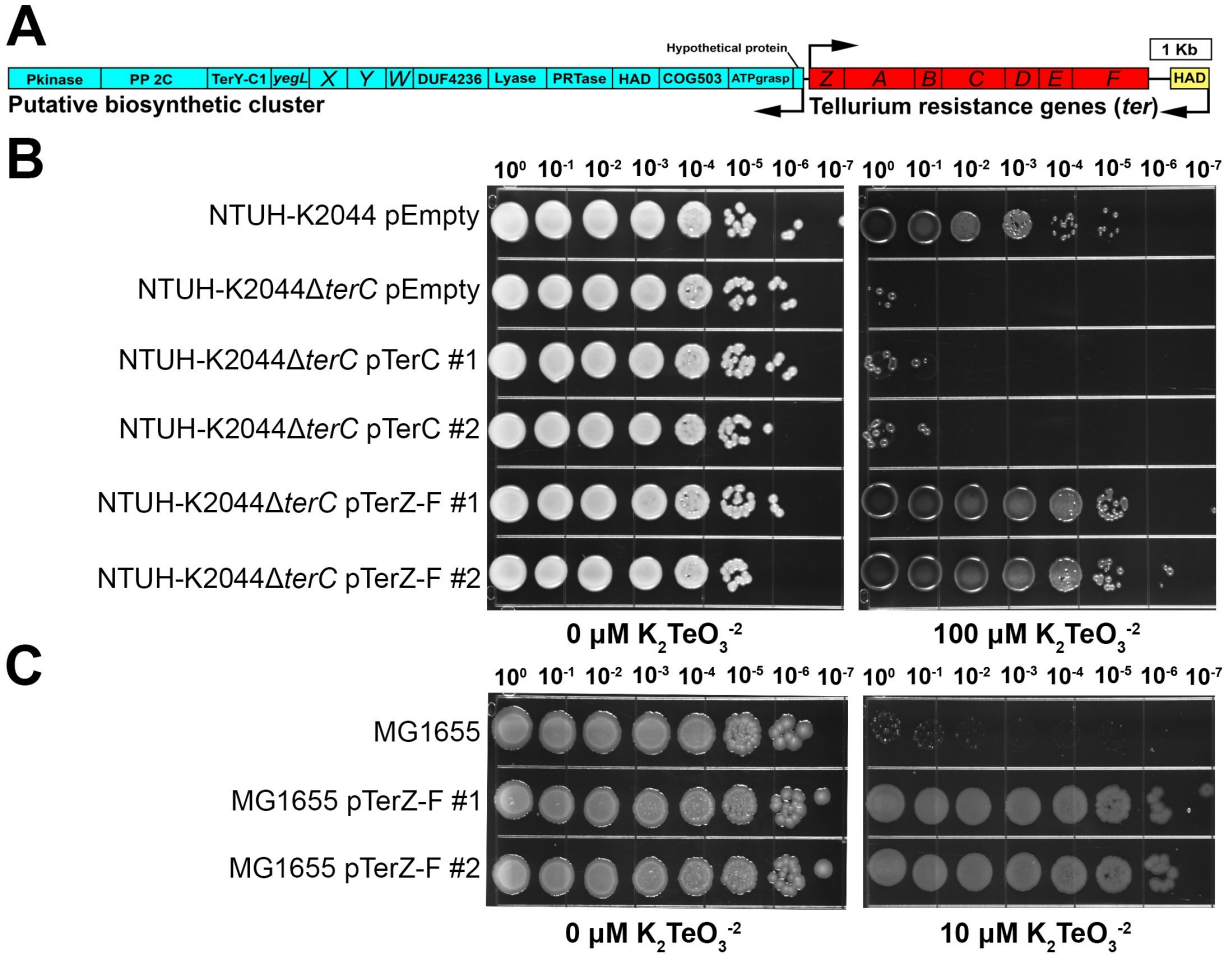
The Kp *terZ-F* genes are sufficient for TeO_3_^-2^ resistance. (A) The *ter* locus is organized in two operons, a putative biosynthetic cluster and a TeO_3_^-2^ resistance cluster. These sections are found on opposite DNA strands and are encoded bidirectionally. The representative *ter* locus from the hvKp strain NTUH-K2044 is shown. NTUH-K2044 containing the empty vector pACYC184, the isogenic Δ*terC* mutant (clone Kp2259) containing an empty vector, the pTerC, or the pTerZ-F plasmid (B), and the *E*. *coli* K12 strain MG1655 with or without the pTerZ-F plasmid (C) were grown on LB or LB containing 10 or 100 μM K_2_TeO_3_^-2^ to visualize inhibition of growth (dilution series 10^0^−10^−7^ of overnight culture). Two representative clones (labeled #1 and #2) of NTUH-K2044Δ*terC* containing the pTerC or the pTerZ-F plasmid and MG1655 containing the pTerZ-F plasmid are shown.

The complete *ter* locus consists of two distinct, but highly conserved, operons encoded on opposite DNA strands: a set of 14 individual genes with potential biosynthetic functionality that includes *terXYW*, and a tellurium resistance operon consisting of *terZABCDEF* (referred to as the “*ter* operon”). This is followed by a predicted haloacid dehydrogenase (HAD) that is highly conserved among *ter*-encoding Kp isolates (Fig 1A and S1 Table). Computational prediction of protein structure and function based on amino acid sequence using I-TASSER [38–40] indicates that many of the *ter* operon proteins are involved in response to stress (S1 Table), which comports with previous studies [21]. The deletion of *terC*, which is predicted to be involved in transmembrane transport (S1 Table), from the pK2044 plasmid renders hypervirulent Kp strain NTUH-K2044 (clone Kp2259) exquisitely sensitive to TeO_3_^-2^ (Fig 1B); however, *terC* expressed *in trans* in a Δ*terC* strain did not restore phenotypic resistance to TeO_3_^-2^ (Fig 1B). Sequencing of a second clone (Kp2257) did not reveal any spurious mutations and demonstrated insertion into the *terC* locus while replicating the sensitive to TeO_3_^-2^ phenotype (S1A and S1B Fig); thus, this phenotype is attributable to insufficient *terC* expression from the plasmid or polar effects of the mutation. The induction of TeO_3_^-2^ sensitivity via polar inactivation of *terDEF* via mutation of *terC* is consistent with previous studies that demonstrate that *terBCDE* are necessary for resistance to TeO_3_^-2^ [28,34]. Expression of *terZ-F in trans* in a Δ*terC* mutant fully restored TeO_3_^-2^ resistance (Fig 1B). Furthermore, the expression of *terZ-F in trans* was sufficient to confer TeO_3_^-2^ resistance to the *Escherichia coli* strain MG1655 (Fig 1C). These results indicate that an intact *ter* operon is necessary for TeO_3_^-2^ resistance in Kp NTUH-K2044.

### The *ter* operon is a genetically independent factor, rather than a biomarker of hvKp

**Fig 2:**
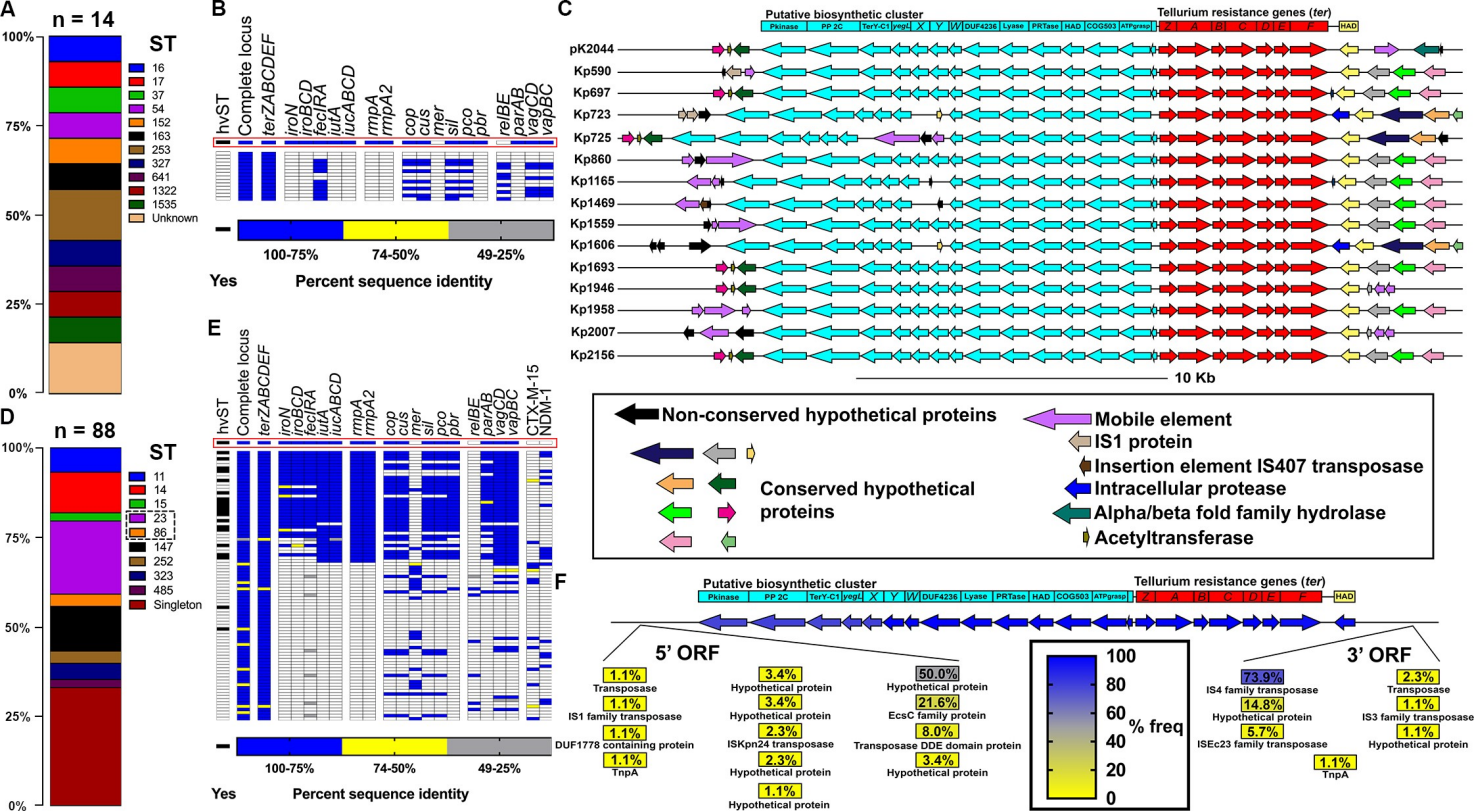
The Kp *ter* operon is not exclusive to hypervirulence plasmids. *ter*+ plasmids from Martin *et al*. mSystems, 2018 [10] (A-C) and reference strains from the NCBI database (D-F) were analyzed. (A,D) Relative frequencies of sequence types (ST) of Kp strains containing *ter*+ plasmids. HvKp sequence types previously associated with the *ter* operon are outlined in a dashed line. (B,E) Heat map of *ter*+ plasmid sequence similarity to genes known to influence infection and antibiotic resistance genes. Each row represents an individual plasmid in the order of S2 Table (Martin *et al*. mSystems, 2018 [10] index 1–14, NCBI reference strains index 15–102). The pK2044 hvKp plasmid is highlighted by the red box, and hypervirulent Kp sequence types (hvST) previously associated with the *ter* operon are indicated. (C,F) To determine if any neighboring gene was consistently associated with *ter*, the gene neighborhood of *ter* plasmids encoding the *ter* operon from Martin *et al*. mSystems, 2018 [10] was visualized (C) and the frequency of ORFs adjacent to the *ter* operon encoded on reference plasmids from the NCBI database was calculated (F).

Next, we sought to determine if *ter* is genetically independent or a biomarker of hvKp, as has been previously reported [35,36]. To this end, we returned to the *ter*-encoding (*ter*+) Kp isolates from our previous study [10]. These isolates were highly diverse, as reflected by their sequence types (Fig 2A) and none were a hvKp sequence type previously associated with the *ter* operon. To determine virulence gene content, we sequenced *ter*-encoding plasmids from our patient isolates using long-read sequencing (Oxford Nanopore Technologies, Oxford, UK). *ter*-encoding plasmids were characteristically large (86.8–430.8 kb; S2 Fig) and some were derived from plasmid fusions (S2 Table). These plasmids displayed substantial sequence variation outside the *ter* locus (S3A and S3B Fig), indicating that the *ter* operon is not a marker for a widely circulating, highly conserved, plasmid. Additionally, these *ter*-encoding plasmids do not contain virulence genes associated with Kp hypervirulence, nor was a single antibiotic resistance gene highly present on these plasmids (Fig 2B and S2 and S3 Tables). This supports the premise that the *ter* operon is an independent fitness factor during infection, rather than a marker of a hypervirulence or antibiotic resistance-encoding plasmid. Finally, predicted open reading frames (ORFs) directly up- and downstream of the *ter* operon displayed minimal functional conservation (Fig 2C), further indicating that this operon is not tightly linked to a virulence factor. Next, we repeated this analysis using publicly available reference genomes of *ter*-encoding Kp isolates (n = 88). These isolates were not limited to hvKp sequence types associated with the *ter* operon (Fig 2D), and indeed, the *ter+* plasmids displayed a high degree of sequence variability (S3C Fig). 42% of *ter*+ plasmids contained a *rmpA/A2* homolog and an accessory iron acquisition system. The remaining 58% *ter*+ plasmids had no classical hypervirulence factor present (Fig 2E and S2 and S3 Tables), and again, the predicted up- and downstream ORFs displayed little functional conservation except for transposase activity (Fig 2F and S1 Table). Together, these results indicate that *ter* is a genetically independent factor, and is likely playing a direct, but yet undescribed role in Kp disease.

To determine the geographical and ecological range of *Klebsiella* encoding the *ter* operon, 14,060 *Klebsiella* sp. genomes, including 1,989 containing *terZ-F*, and their associated metadata were extracted from the Pathosystems Resource Integration Center (S1 Data) [41]. These genomes were derived from *Klebsiella* sp. strains from 6 of 7 continents (S4A Fig), indicating a wide geographical range that corresponds to the environmental ubiquity of *Klebsiella* sp. Assessment of the specific source of *Klebsiella* sp. isolation indicates a wide variety of hosts, including both animals and plants, and a number of environmental sources (S4B Fig). Interestingly, we found that *terZ-F* containing isolates were evenly distributed amongst all isolation sources (~14% of all isolates, S4C Fig); however, humans are the most represented isolation source, which is not surprising given the over-representation of human isolates in bacterial genome repositories. Many *ter*-containing *Klebsiella* sp. were isolated from human gut, blood, and respiratory samples (S4B and S4D Fig), which both supports a role for the *ter* operon in the human gut and comports with our previous studies where we originally identified a strong association between the *ter* operon and infection in Kp colonized patients [10]. Overall, relatively few *ter*-containing *Klebsiella* sp. isolates came from liver abscesses (S4D Fig), which are traditionally associated with hvKp [36], although they were enriched in this infection site (S4D Fig). Overall, *ter*-containing strains have a wide geographical and ecological range and are found across multiple sites of human infection.

### TerC is a microbiome-dependent gut fitness factor

**Fig 3:**
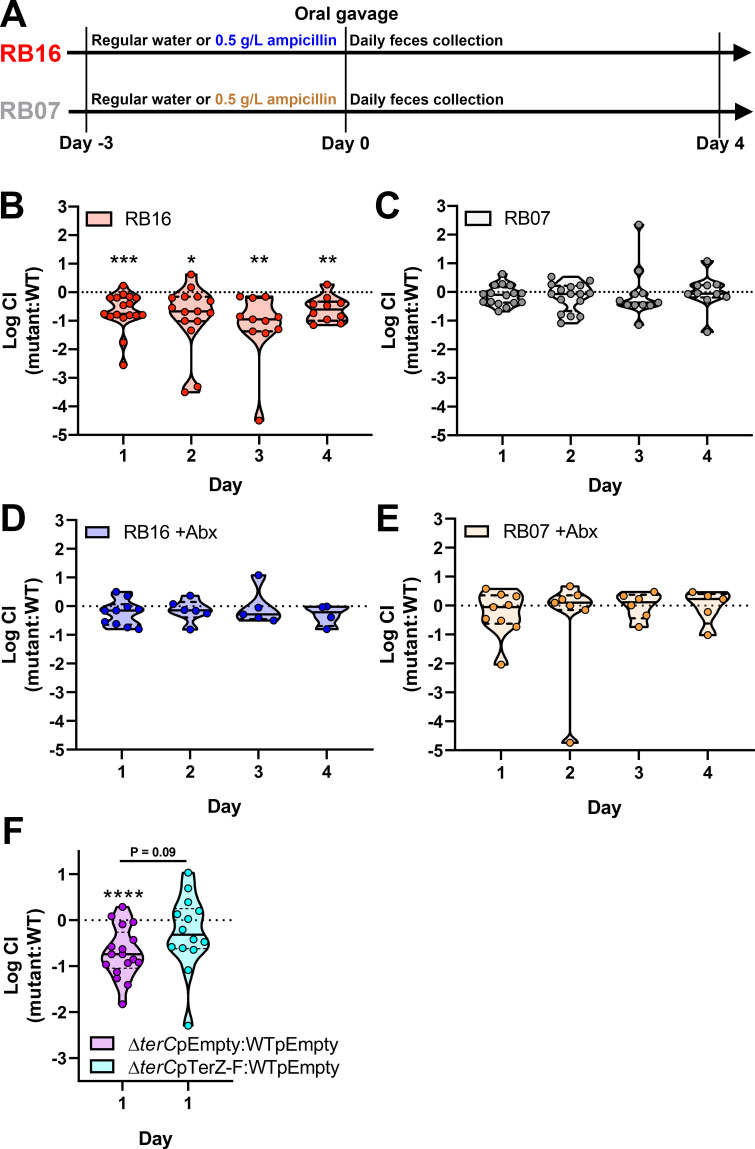
TerC is a fitness factor during gut colonization. (A) Three days prior to inoculation, male and female C57BL6/J mice sourced from barriers RB16 and RB07 were treated with 0.5 g/L ampicillin or regular drinking water. (B-E) NTUH-K2044 and the isogenic Δ*terC* mutant (clone Kp2259) were mixed 1:1 and approximately 5x10^6^ CFU were orally gavaged into mice (n = 9–18 per group). A fresh fecal pellet was collected daily from each animal, CFUs were enumerated, and log competitive indices (mutant:WT) were calculated (median and IQR displayed, *P < 0.05, **P < 0.005, ***P < 0.0005, one-sample *t* test compared to a hypothetical value of 0). (F) NTUH-K2044 and the isogenic Δ*terC* mutant containing an empty vector or the pTerZ-F plasmid were mixed 1:1 and approximately 5x10^6^ CFU were orally gavaged into mice sourced from barrier RB16 (n = 14–16). A fresh fecal pellet was collected 24 hours after inoculation, CFUs were enumerated, and log competitive indices (mutant:WT) were calculated (F, median and IQR displayed, ****P < 0.00005, one-sample *t* test compared to a hypothetical value of 0 or Student’s *t* test). Each data point represents an individual animal.

We previously reported a strong association between the *ter* operon and Kp infection (pneumonia and bacteremia) in Kp colonized patients, yet also found that *terC* is dispensable in a murine model of pneumonia [10]. To determine if the *ter* operon is important for bacteremia, WT Kp and Δ*terC* were competed in a peritoneal injection model of murine bacteremia. *terC* was dispensable in all tissues with the exception of a modest defect in the brain (S5 Fig). We did not explore this finding further, as a role in meningitis would not explain the correlation between the *ter* operon and infections observed in patients. We then hypothesized that the *ter* operon may be required during gut colonization, which precedes infection [11,12]. Exposure to antibiotics was not associated with Kp colonization or subsequent infection in our intensive care unit patient population [10,42], indicating that Kp must contend with the indigenous microbiota to colonize the gut and cause infection. Therefore, C57BL/6J mice were sourced from two different housing sites at The Jackson Laboratory (barriers RB16 and RB07) to control for natural variations in the gut microbiota induced by housing conditions [17–20]. Mice were orally gavaged with 100 μL of a mixture of wild-type and Δ*terC* Kp (Fig 3A). Intriguingly, a fitness defect (median 5.8-, 4.7-, 8.9-, and 4.0-fold-defect on days 1–4, respectively) was observed consistently for Δ*terC* in the mice sourced from RB16 over several days (Figs 3B and S6A) but not from RB07, despite their genetic identity (Figs 3C and S6B). These data suggest that the fitness defect exhibited by Δ*terC* is dependent on the gut microbiota.

To begin to characterize the effect of the indigenous microbiota on the Δ*terC* mutant, mice were treated with antibiotics and the experiment was repeated. Consistent with previous studies [15], treatment with antibiotics increased overall Kp colonization density in mice from both barriers (S6C and S6D Fig); however, this treatment also restored the fitness of Δ*terC* in mice sourced from RB16 (Figs 3D and S6C). Conversely, antibiotic treatment of mice sourced from RB07 did not impact Δ*terC* fitness (Figs 3E and S6D). These data indicate that an antibiotic-susceptible member or members of the microbiota of mice sourced from RB16 are involved in reducing the fitness of the Δ*terC* mutant, as opposed to the microbiota of mice sourced from RB07 enhancing Δ*terC* fitness. Furthermore, complementation of the Δ*terC* mutant by expression of *terZ-F in trans* ameliorated its fitness defect in mice sourced from RB16 (Fig 3F). This finding was confirmed with the fully sequenced Δ*terC* clone (S1C Fig). To determine if the *ter* operon is required for colonization, mono-colonization studies were performed. These results show that both the wild type and Δ*terC* mutant were able to colonize the gut (S7 Fig), although there was mouse-to-mouse variation that may obfuscate an advantage of the *ter* operon during mono-colonization. Consistent with previous studies [43–46], the mice exhibited mortality throughout the duration of the experiment. However, the increased bacterial load due to antibiotics (S6 Fig) was not associated with increased mortality, and both strains exhibited equal virulence in this model regardless of barrier (S8 Fig). Collectively, these data suggest that the *ter* operon is a microbiome-dependent gut fitness factor.

### Reduced fitness of the Δ*terC* mutant is associated with specific gut microbiota constituents

**Fig 4:**
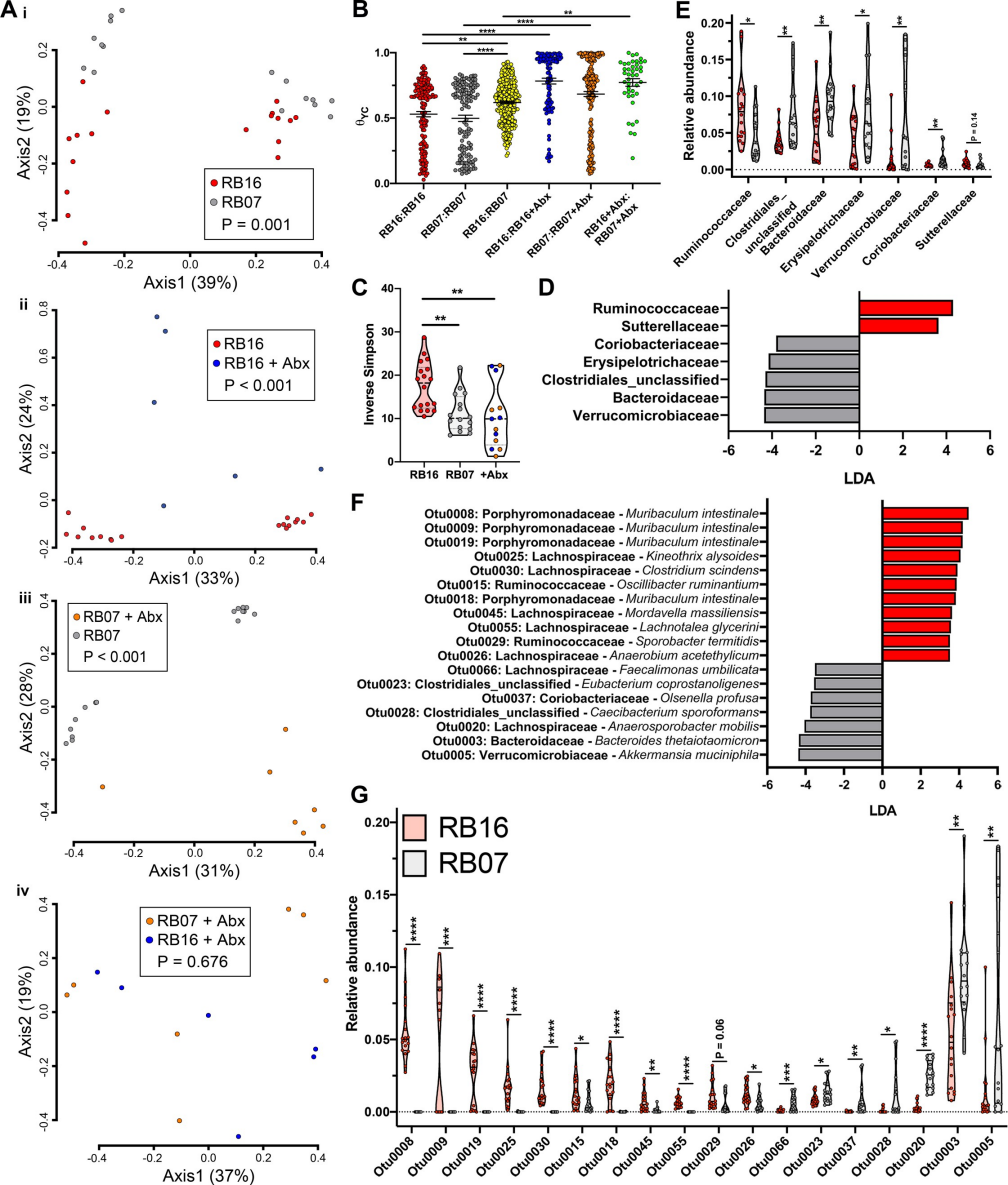
The fecal microbiota in which *terC* is (RB16) and is not (RB07) a fitness factor are distinct. Fecal pellets collected from male and female C57BL6/J mice sourced from barriers RB16 and RB07 (n = 9–20 mice per group) on the day of Kp inoculation were subjected to 16S rRNA sequencing. Pairwise community dissimilarity values between the fecal microbiota communities of barriers RB16 and RB07 with or without three days treatment with 0.5 g/L ampicillin were visualized by Principal coordinates analysis (A, AMOVA) and individually (B, **P < 0.005, ****P < 0.00005, one-way ANOVA followed by Tukey’s multiple comparisons post-hoc test). (C) Diversity of the fecal microbiota was summarized by inverse Simpson index (blue points: RB16+Abx, orange points: RB07+Abx, **P < 0.005, one-way ANOVA followed by Tukey’s multiple comparisons post-hoc test). LEfSe was used to determine if specific bacterial families (D) or OTUs (F) were differentially abundant between the fecal microbiota of RB16 and RB07 (D, LDA ≥ 3.5 and P < 0.05 are shown). Differential bacterial families (E) or OTUs (G) relative abundance values were plotted (E, *P < 0.05, **P < 0.005, ***P < 0.0005, ****P < 0.00005, Student’s *t* test).

To determine if the composition of the gut microbiota of mice sourced from RB16 and mice sourced from RB07 differed, we performed 16S rRNA gene sequence analysis from fecal DNA collected throughout the course of these experiments (S9 Fig and S2 Data). To compare the microbiota between all groups (mice sourced from RB16, RB07, RB16+Abx, RB07+Abx) and all time points, θ_YC_ distances [47] were calculated, and principal coordinates analysis was used to visualize these distances. θ_YC_ dissimilarity accounts for both the number of shared and unique species as well as differential species abundance in a single metric [47]. As expected, microbiota differed between male and female mice (Fig 4A.i, axis 1, females cluster on left of graph) [48–50]. Despite sex-based differences, the fecal microbiota of RB16 and RB07 were significantly dissimilar on the day of inoculation (Fig 4A.i, axis 2, AMOVA P = 0.001; Fig 4B), suggesting that the results observed in Fig 3B–3C were attributable to differences in the microbiota of these mice. In addition, the fecal microbiota of antibiotic treated mice sourced from RB16 and RB07 were dissimilar from their untreated counterparts (Fig 4A.ii and iii, AMOVA P < 0.001), but not from one another (Fig 4A.iv, AMOVA P = 0.676). Assessment of the magnitude of dissimilarity indicated that intergroup dissimilarity was higher than intragroup dissimilarity, and antibiotic treatment resulted in the greatest dissimilarity (Fig 4B, higher values indicate higher dissimilarity). These findings were consistent across all time points (S10–S14 Figs). These data demonstrate an association between *ter*-dependent fitness in the gut and the composition of the gut microbiota and suggest that an individual or group of gut microbiota constituents in the mice sourced from RB16 underlies the observed loss of fitness.

There were several differences between the microbiota in mice sourced from RB16 and RB07. The diversity of the fecal microbiota of mice sourced from RB16 was significantly higher than mice sourced from RB07 on the day of inoculation (Fig 4C), and this difference was present throughout the experiment (S15 Fig). We next sought to determine if a bacterial family or families differentiated the fecal microbiota of mice sourced from RB16 and RB07 through the use of linear discriminant analysis (LDA) effect size (LEfSe) [51]. On the day of inoculation, 7 bacterial families were found to be differentially abundant, 2 of which were more abundant in mice sourced from RB16 and 5 of which were more abundant in mice sourced from RB07 (Fig 4D and 4E). Only unclassified Clostridiales, associated with mice sourced from RB07, remained differential throughout the entire experiment (S16 and S17 Figs). We hypothesized that no family consistently distinguished mice sourced from RB16 from mice sourced from RB07 because variations in individual operational taxonomic unit (OTU) relative abundances obscured family-level analysis. As such, we used LEfSe to determine differentially abundant OTUs between these microbiotas. On the day of inoculation, 18 OTUs were found to be differentially abundant, 7 of which were more abundant in mice sourced from RB07, and 11 of which were more abundant in mice sourced from RB16 (Fig 4F). Intriguingly, 4 of the 11 OTUs associated with mice sourced from RB16 had 16S rRNA sequences most similar to *Muribaculum intestinale* (Fig 4F; 0008, 0009, 0018, 0019) [52–54]. These 4 OTUs, as well as 0030 which had 16S rRNA sequence most similar to *Clostridium scindens*, remained more abundant in mice sourced from RB16 than RB07 for the entire experiment (S18 and S19 Figs). Notably, the Porphyromonadaceae family, of which *M*. *intestinale* was considered a member in our dataset, was also enriched in mice sourced from RB16 on days 1–3 post-inoculation (S16 and S17 Figs). The identification of these species as differentially abundant in mice sourced from RB16 is notable, as *M*. *intestinale* belongs to family S24-7 which is suggested to play a role in gut inflammatory homeostasis [20,54–57], and *C*. *scindens* has been reported to be a clinically relevant probiotic candidate [58]. The only OTU that was more abundant in mice sourced from RB07 throughout the experiment is 0037, which is most similar to *Olsenella profusa* (Figs 4F and S18 and S19). OTUs associated with mice sourced from RB16 by LEfSe were mostly absent in mice sourced from RB07 (Fig 4G) throughout the course of the experiment (S18 and S19 Figs). If these OTUs are tightly associated with the observed Δ*terC* mutant fitness defect, they should also be sensitive to the antibiotic treatment that ameliorates the defect. Indeed, differential OTUs, as well as corresponding families, were sensitive to antibiotic treatment (S20 and S21 Figs) highlighting their potential role in reducing the fitness of the Δ*terC* mutant. Finally, we asked if the introduction of Kp impacted the relative abundance of these families and OTUs. At the family level, the microbiota of mice sourced from RB16 was only minimally modulated (S22A Fig) by Kp inoculation, and the only OTU that differentiated mice sourced from RB16 from RB07 that was modulated was 0045 (*Mordavella massiliensis*, S21B Fig). Further analysis of the gut communities of mice sourced from RB16 and RB07 demonstrated that these communities remained stable through the course of the experiment in antibiotic-naïve mice but shift drastically following antibiotic treatment (S22C Fig). These data indicate that the microbiota constituents of mice sourced from RB16 reduce the fitness of the Δ*terC* mutant and are highly stable during Kp colonization.

### SCFA metabolism is predicted to be enriched in the gut of mice sourced from RB16

Next, we interrogated what biological processes may result in the reduced fitness of the Δ*terC* mutant. Specifically, we were interested in exploring biological processes that were characteristic of the microbiota constituents of mice sourced from RB16 associated with reduced fitness of the Δ*terC* mutant. To this end, the metagenomes of the fecal microbiota of mice sourced from RB16 and RB07 were predicted using PICRUSt2 [59]. The relative abundance of predicted metabolic pathways as annotated by MetaCyc [60] differed between the fecal microbiota of mice sourced from RB16 and RB07 (S23 Fig and S3 Data). A significant difference overall between predicted metabolic pathways of the fecal microbiota of RB16 and RB07 was detected on the day of inoculation and on multiple days post-inoculation although this was not readily detected in the first two principal components that account for the majority of variation in the data (S23 Fig). The subtlety of this finding was not surprising, as the microbiota of mice sourced from RB16 and RB07 are distinct based on specific OTUs (Fig 4) but share a large number of gut constituents (S9 Fig). Thus, we expected that a limited number of predicted metabolic pathways would differentiate microbiota of mice sourced from RB16 and RB07, and that some of these pathways would correspond to the microbiota constituents of mice sourced from RB16 associated with the reduced the fitness of the Δ*terC* mutant.

LEfSe was used to determine which specific metabolic pathways differentiated the predicted metagenomic profiles of the gut microbiota of mice sourced from RB16 and RB07 [51]. This analysis revealed several metabolic pathways that were enriched in the gut microbiota of mice sourced from RB16 on the day of inoculation and throughout the experiment, including gluconeogenesis, peptidoglycan biosynthesis, and fermentation of short-chain fatty acids (S24 and S25 Figs, S4 Table and S3 Data). The enrichment of SCFA metabolic pathways in the gut microbiota of mice sourced from RB16 may be explained by the presence of *M*. *intestinale*, which was strongly associated with the gut microbiota of mice sourced from RB16. In addition, antibiotic treatment of mice sourced from RB16 led to a decrease in OTUs that correspond to *M*. *intestinale* (0008, 0009, 0018, 0019), and a drastic reduction in the relative abundance of predicted SCFA metabolic pathways (S26 Fig). This corresponds to our 16S rRNA sequencing data, as SCFA pathways underpin the impact of *M*. *intestinale* on gut inflammatory homeostasis [20,54–57].

### Exogenous SCFA administration reduces the fitness of the Δ*terC* mutant in the gut

SCFAs are known to have a wide variety of functions in the host, including increasing antimicrobial peptide production [61], intestinal epithelial barrier function [62], and acceleration of the immune response to Enterobacteriaceae [63]. Moreover, previous studies have identified a protective role for the SCFA acetate during Kp lung infection [64]. To determine if SCFA metabolism is responsible for the fitness defect exhibited by Δ*terC*, we first explored if SCFAs directly kill or inhibit the growth of Kp in a *ter*-dependent manner. Previous studies have shown that SCFAs are able to directly inhibit the growth of Kp under acidified conditions through disruption of respiration [65]. Thus, we grew WT Kp and Δ*terC* in the presence of SCFAs (acetate, butyrate, and propionate) in both neutral and acidified conditions. Similar to previous studies, high concentrations of SCFAs slightly inhibited growth of both strains in neutral conditions, but completely arrested growth in acidic conditions (S27A Fig). To determine if an individual SCFA was responsible for growth inhibition, WT Kp and Δ*terC* was grown in acetate, butyrate, and propionate individually and in combination in both neutral and acidified conditions. Acetate and butyrate had the largest impacts on Kp growth; however, growth inhibition was not dependent on the presence of *terC* (S27B Fig). Next, we assessed if growth inhibition occurs in a *ter*-dependent manner at lower concentrations of SFCAs. Again, titration of SCFAs in acidified media inhibited growth in a concentration-dependent manner, but not a *ter*-dependent manner (S27C Fig). To determine if WT Kp are able to antagonistically inhibit the growth of Δ*terC* in the presence of SCFAs, competitive growth assays were performed in the presence of SCFAs. No antagonism was observed between WT Kp and Δ*terC* in the presence of SCFAs (S27D Fig). Finally, we performed killing assays with the WT Kp and Δ*terC* strains to determine if SCFAs can kill Kp in a *ter*-dependent manner; however, no killing was observed for either strain (S27E Fig). Collectively, these data demonstrate that SCFAs can indeed inhibit Kp growth under acidified conditions as previously described [65], though not in a *ter*-dependent manner.

**Fig 5:**
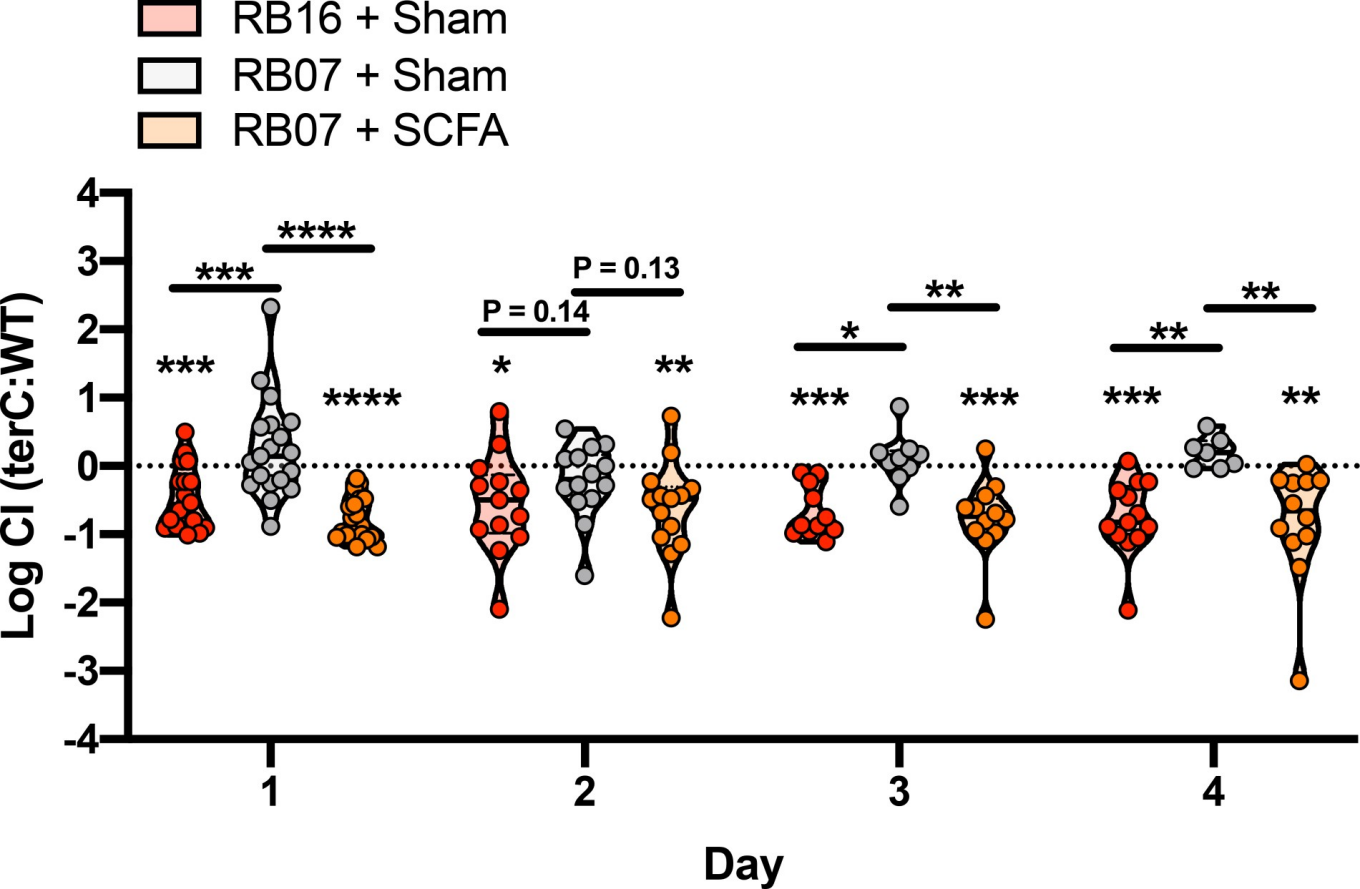
Exogenous treatment of mice with SCFAs results in a *terC* fitness defect. Seven days prior to inoculation, male and female C57BL6/J mice sourced from barriers RB16 and RB07 were treated with a SCFA cocktail or regular drinking water (sham). NTUH-K2044 and the isogenic Δ*terC* mutant (clone Kp2257) were mixed 1:1 and approximately 5x10^6^ CFU were orally gavaged into mice (n = 19 per group). A fresh fecal pellet was collected daily from each animal, CFUs were enumerated, and log competitive indices (mutant:WT) were calculated (median and IQR displayed, *P < 0.05, **P < 0.005, ***P < 0.0005, one-sample *t* test compared to a hypothetical value of 0 or Holm-Sidak multiple-comparison test following one-way ANOVA).

To assess the effect of SCFAs on Kp gut fitness *in vivo*, we repeated our competitive gut colonization experiments with mice sourced from RB16 and RB07, but also included mice sourced from RB07 treated continuously with a cocktail of SCFAs via drinking water. As previously observed, Δ*terC* Kp were less fit than WT Kp in mice sourced from RB16 but were equally fit in mice sourced from RB07 (Fig 5). Treatment of mice from RB07 with a SCFA cocktail led to significantly reduced fitness of the Δ*terC* mutant in the gut similar to that of what was observed in RB16 (Fig 5). Finally, we measured the presence of SCFAs in the fecal pellets of mice sourced from both barriers, as well as mice sourced from RB07 treated with exogenous SCFAs. Fecal SCFA quantification revealed higher SCFA concentrations in the feces of mice sourced from RB16 compared to those from RB07 (S28A Fig); however, this difference did not reach statistical significance. Treatment of mice sourced from RB16 with antibiotics, which restored the Δ*terC* fitness defect, also significantly reduced fecal SCFA levels (S29A Fig), specifically acetate and butyrate (S29C and S29D Fig). These data indicate that the reduced fitness of the Δ*terC* mutant is due to gut SCFA levels, which is associated with differences in specific indigenous gut microbiota constituents between mice sourced from RB16 and RB07.

## Discussion

The work presented here advances our understanding of Kp colonization and the transition to infection by combining the study of both host and pathogen to describe a factor involved in this process: the *ter* operon. Our previous work demonstrated a role for *ter* during Kp infection [10], therefore indicating that *ter* is important in the infectious process. Given that prior gut colonization often precedes disease [11–13,66], our finding that the *ter* operon is necessary for gut fitness in a microbiome-dependent manner corroborates our previous studies and explains why the *ter* operon is dispensable in murine models of pneumonia [10] and bacteremia but strongly associated with human infection in these sites. The importance of the *ter* operon is validated by the demonstration that this operon is genetically independent, rather than a marker of the Kp hypervirulence plasmid. Finally, we have identified a set of microbes that impact the fitness of Kp strains in a *ter*-dependent manner, likely through SCFA metabolism. The role of *ter* in resisting SCFAs is based on findings that 1) *M*. *intestinale* is a primary fermenter capable of producing SCFAs and is uniquely present in the restrictive gut microbiota of mice sourced from RB16 and 2) administration of SCFAs to mice sourced from RB07, which lack *M*. *intestinale*, results in diminished Δ*terC* fitness. Moreover, the association between the antibiotic-mediated reduction of *M*. *intestinale* from the gut of mice from RB16 and a significant reduction in fecal SCFAs support this conclusion. Collectively, these results show how a bacterial factor can interact with its host’s microbiome to enhance colonization, which may increase risk of infection in humans.

Gut colonization is a critical first step for many pathogens that cause both intestinal and extra-intestinal infection. Kp, including ESBL-, CP-, and hvKp, can be isolated from multiple sites of colonization, including the gut, nasopharynx, and skin. While skin colonization is considered a transient event [67], colonization of mucosal sites is an important event preceding many cases of Kp disease [10–14]. Few factors have been identified that influence the transition from colonization to infection, either in a hypothesis-driven or systematic manner [15,43,68–71]. Notably, published systematic studies rely on the administration of antibiotics to permit gut colonization [69–71], and therein are identifying Kp gut colonization factors in the absence of an intact microbiota. Disruption of the gut microbiome can lead to the expansion of potential pathogens in the gut and increased susceptibility to infection [72–75]; however, our prior analysis of Kp colonization in over 2,400 patients found high rates of colonization (up to 17%) but no positive association between colonization and prior antibiotic exposure [42]. This indicates that Kp can colonize the gut in the absence of microbiome perturbation. Moreover, highly antibiotic-resistant bacteria have been isolated from otherwise healthy adults [76–80], suggesting that antibiotic-resistant bacteria are also able to invade intact microbiomes. An attractive hypothesis is that Kp circumvents microbiota-mediated colonization resistance by occupying newly accessible niches following antibiotic disruption of the gut microbiome. Yet, this fails to address how Kp invades the intact gut microbiome in the absence of antibiotic exposure. This study indicates an alternative means of circumventing microbiota-mediated colonization resistance, wherein specific Kp factors enhance fitness during invasion of the intact gut microbiome. In this case, the *ter* locus is needed for optimal fitness in the presence of certain indigenous gut microbes, suggesting that acquisition of *ter+* plasmids can expand the host range of a pathogen by resisting the competitive pressures of these bacteria. This evasion of colonization resistance by horizontal gene transfer echoes the bacterial arms race seen in response to nutritional immunity and antibiotics.

To be fit in the gut environment, Kp must overcome the stress of direct interspecies competition, nutrient limitation, and anti-microbial stress induced by the indigenous microbiota (extensively reviewed in [81]). Kp has received increased notoriety for its ability to compete in stressful polymicrobial environments [82–84]; however, relatively little is known about how Kp gains an advantage over its competitors in the gut. The hvKp T6SS has been implicated in direct interspecies competition in the gut [43], and the trehalose-6-phosphate hydrolase [45] and cellobiose-specific PTS transporter CelB [46] have been suggested to play a role in gut nutrient acquisition when the indigenous microbiota is present. Additionally, the Sap (Sensitivity to antimicrobial peptides) transporter [85] and the acid-sensitive transcriptional regulator CadC [68] are important for optimal gut fitness in the presence of the indigenous microbiota. Our data indicates that the indigenous gut microbiota of mice sourced from RB16 create an environment that limits Kp fitness via SCFA metabolism when a functional *ter* operon is absent. Notably, many of the OTUs that differentiate the gut microbiota of mice sourced from RB16 from RB07 correspond to *M*. *intestinale* (also known as S24-7), which are associated with increased SCFA levels [86] and known to influence gut inflammatory homeostasis. Mice treated with a consortium of bacteria containing *M*. *intestinale* by oral gavage were more resistant to *Salmonella typhimurium* infection in an inflammation-dependent manner [20]. Additionally, depletion of the S24-7 family of bacteria, which includes *M*. *intestinale*, from the gut has been associated with lower expression of antimicrobial peptides [57], increased inflammatory cytokines [56], and increased gut permeability [56,57]. Interestingly, another study incidentally demonstrated an inverse relationship between family S24-7 abundance and Kp gut colonization density [15]. Thus, the *ter* operon enhances Kp fitness, likely by resisting stress induced by specific indigenous gut microbiota via SCFA metabolism.

The characterization of the *ter* operon as a microbiome-dependent gut fitness factor is of interest, as the biology of this operon has long been enigmatic. It is possible that the *ter* operon facilitates a general stress response and is not specific to TeO_3_^-2^.The antibacterial properties of TeO_3_^-2^ are attributable to its ability to disrupt membrane stability and strong oxidizing action [27,87–89]. The reduction of TeO_3_^-2^ results in the formation of superoxides, leading to abrogation of DNA synthesis, protein synthesis and exhaustion of cellular reductases [90,91]. The diversity of stress induced by TeO_3_^-2^, in combination with corresponding studies confirming these stresses, accounts for the pleotropic characterization of the *ter* operon; however, these studies fall short of concluding that the *ter* operon is necessary for response to a specific physiologically relevant stress. Further complicating this paradigm, other factors are often implicated in the response to TeO_3_^-2^-induced stress, such as superoxide dismutase [27] or 6-phosphogluconate dehydrogenase [92]. Studies aimed at understanding the regulation of the *ter* operon have provided some insight, as they have suggested transcriptional control by OxyR [29,32]. While these studies further connect the *ter* operon to oxidative stress, we were unable to identify an OxyR binding motif in the promoter region of the Kp *ter* operon. The chromosomal location of the *ter* operon in these studies [29,32], as opposed to the plasmid locality in Kp, may account for this difference. In lieu of a clear physiologically relevant stress or an obvious regulator, *in silico* studies have suggested the gene products of *terZABCDEF* form a membrane-associated complex with TerC acting as the transmembrane protein ([21] and S1 Table). This complex is thought to sense a soluble ligand or regulate membrane permeability, therein linking the extracellular and intracellular environments [21]. Our *in silico* data is consistent with many of the predicted functions of the *ter* operon gene products [21], and some experimental evidence exists supporting the formation of a membrane-associated TerC complex [93]. The putative biosynthetic cluster is yet more complicated and is predicted to synthesize a nucleoside-like metabolite or potentially be involved in DNA processing [21], though it is unclear how the TerC complex would functionally interact with this gene cluster or its products. In addition to TeO_3_^-2^, *ter* in non-Kp species has been implicated in resistance to phage and colicins that can be found in the gut [28], the latter being antibacterial proteins produced by bacteria to kill their competitors [94]. It may be the case that the presence of SCFAs or SCFA-producing bacteria create a gut environment where Kp encounters these stresses more frequently.

Despite their simplicity, SCFAs are one of the most important gut metabolites. In the gut, SCFAs are products of bacterial fermentation, the most common of which are acetate, butyrate, and propionate, which have many critical functions in colonization resistance. First, they are able to directly inhibit bacterial growth through intracellular acidification [65,95]. We observed a dose-dependent growth inhibition of Kp by SCFAs; however, this was independent of the presence of a functional *ter* operon, suggesting that SCFAs negatively impact Δ*terC* fitness in the gut by a different mechanism. Alternatively, the TerC protein is a predicted proton symporter, and therein may be able to sense intracellular pH, leading to regulation of other genes necessary for complete fitness in the gut in response to pH perturbations caused by SCFAs that would not be observable *in vitro*. Regardless, SCFAs are highly plastic in their function. Second, SCFAs are important for maintaining immune homeostasis (reviewed in [96]). This function is critical in the context of colonization resistance, as SCFAs reduce epithelial oxygenation [97,98] and stimulate antimicrobial peptide expression in the gut [61], leading to reduced pathogen colonization. Interestingly, SCFAs have been shown to accelerate the immune response to Enterobacteriaceae in the gut [63]. Interestingly, gut microbiota-influenced inflammatory homeostasis has been shown to influence Kp gut colonization via IL-36 signaling though this was not linked to SCFAs [16]. Notably, the SCFA that showed the largest difference between mice sourced from RB16 compared to those from RB07 was acetate. This is notable since *M*. *intestinale* is known to produce SCFAs, and specifically acetate [55]. Therefore, stimulation of immune pathways via microbiota derived SCFAs may reduce the fitness of the Δ*terC* mutant in the gut. Alternatively, Kp strains encoding the *ter* operon may antagonize Kp strains that lack the *ter* operon in the gut in an SCFA- or microbiome-dependent manner. Though we explored antagonism between the WT Kp and Δ*terC* strains, these experiments were performed under *in vitro* conditions and do not rule out the possibility of *in vivo* antagonism. Finally, SCFAs may induce production of metabolites from members of the indigenous gut microbiota that reduce Δ*terC* fitness in the gut. The varied impacts of SCFAs on gut homeostasis may explain why exogenous SCFA administration significantly reduces the fitness of the Δ*terC* strain while only subtle differences in fecal SCFA concentration were observed. Importantly, absorption and/or metabolism of these substrates by the host in the small intestine, cecum, proximal colon, or by the indigenous microbiota may alter gut homeostasis while simultaneously masking large differences in fecal SCFA concentrations [99,100]. In summary, the *ter* operon represents a novel, transferrable locus that enhances fitness of Kp NTUH-K2044 in the presence of specific gut microbiota and is associated with increased risk of infection in hospitalized patients. Given the breadth of genetic diversity exhibited by Kp [101], further studies with additional strains are necessary to determine the full impact of the interaction between SCFAs and the *ter* operon on Kp gut fitness. As interventions that modulate gut homeostasis, such as fecal microbiota transplants and administration of SCFA-producing probiotic bacteria, become more common, an understanding of how pathogens are able to overcome these barriers to colonization will be critical to ensure their success.

## Methods

### Ethics statement

This study was performed in strict accordance with the recommendations in the *Guide for the Care and Use of Laboratory Animals* [102]. The University of Michigan Institutional Animal Care and Use Committee approved this research (PRO00007474).

### Materials, media, and bacterial strains

All chemicals were purchased from Sigma-Aldrich (St. Louis, MO) unless otherwise indicated. *E*. *coli* K12 strain MG1655, Kp strain NTUH-K2044 [103], and isogenic mutants were cultured in Luria-Bertani (LB, Becton, Dickinson and Company, Franklin Lakes, NJ) broth at 37°C with shaking, or on LB agar at 27°C (Thermo Fisher Scientific). The isogenic Δ*terC* mutant was generated as previously described [10]. Briefly, the *λ-*red mutagenesis system was used to inactivate the *terC* gene [104]. Electrocompetent NTUH-K2044 cells encoding the pKD46 plasmid were transformed with a kanamycin resistance cassette amplified from the pKD4 plasmid containing homologous overhangs to the *terC* locus. Transformed cells were recovered overnight at 30° C in SOC media, then selected in the presence of 40 μg/mL kanamycin. The isogenic Δ*terC* mutant was confirmed by whole-genome sequencing using the Illumina NexteraXT kit on the Illumina MiSeq using a 2x250 bp V2 kit. Illumina reads are available on the Sequence Read Archive (SRA) in BioProject PRJNA464397. To construct the pTerC and pTerZ-F complementation plasmids, PCR products derived from WT NTUH-K2044 containing the *terC* or *terZABCDEF* open reading frames were inserted into pCR 2.1 using TOPO TA cloning (Life Technologies, Carlsbad, CA) and directionally ligated into pACYC184 following digestion with Xbal and HindIII. The ligation mixture was transformed into NEB 10-beta Competent *E*. *coli* (New England Biolabs, Ipswich, MA) by heat shock. *E*. *coli* transformants were selected at 37°C on LB agar containing 30 μg/ml chloramphenicol, re-cultured, and confirmed by colony PCR. Single transformants were then grown in batch culture for plasmid extraction using the Plasmid Midi Kit (Qiagen, Germantown, MD). MG1655 and NTUH-K2044 competent cells were prepared as previously described [105], electroporated with the complementation plasmids or corresponding empty vector, and selected at 37°C on LB agar containing 30 (MG1655) or 80 μg/ml (NTUH-K2044) chloramphenicol. Following selection, transformants were re-cultured, and confirmed by colony PCR and by growth in presence of TeO_3_^-2^. All primer sequences can be found in S5 Table. For all subsequent experiments, complemented and control strains were grown in the presence of the appropriate antibiotic.

### *ter+* genome identification

For plasmid analysis, *ter*-encoding reference strains and plasmids from the National Center for Biotechnology Information (NCBI) nucleotide collection database were identified using BLAST [106], wherein the entire *ter* locus (S3 Table) was used as the query, and *Klebsiella pneumoniae* (taxid:573) as the subject (extraction date 03/27/2019). The *ter* operon was not identified on any Kp chromosomes. For identification of *Klebsiella* sp. encoding the *ter* operon, individual PATRIC Global Family annotations corresponding to the NTUH-K2044 *terZABCDEF* gene products were searched against the Pathosystems Resource Integration Center (PATRIC) genome database [41]. The resulting list of genomes and corresponding metadata (extraction date 11/16/2020) was then restricted to *Klebsiella* sp. and further refined by identifying genomes that have every NTUH-K2044 *terZABCDEF* gene product annotation and are of good quality as noted by PATRIC. Metadata was visualized in R (v.3.6.3) using the “ggplot2,” “ggmap,” “maps,” and “mapdata” packages and Prism 8 (GraphPad Software, La Jolla, CA).

### Plasmid sequencing and analysis

To characterize *ter*-encoding Kp strains, the multi-locus sequence type (MLST) was assigned using the Bacterial Isolate Genome Sequence Database (BIGSdb) [107,108]. To characterize the *ter*-encoding plasmids from our previous study [10], genomic DNA was extracted from pure Kp cultures using the DNeasy PowerSoil Pro Kit (Qiagen, Hilden, Germany). Long-read genomic sequencing was performed using GridION X5 (Oxford, England) sequencing instrument. Each Nanopore sequencing library was prepared using 1 μg of DNA with the 1D ligation kit (SQK-LSK108, Oxford Nanopore Technologies) and sequenced using R9.4.1 flowcells (FLO-MIN106). MinKNOW software was used to collect sequencing data. Nanopore reads were called using Albacore v2.2.3 and assembled using Canu v1.7 [109]. Assemblies were corrected for ten rounds with Illumina reads using Pilon v1.22 [110] in conjunction with the bowtie2 v2.3.3.1 aligner [111]. Assembled plasmid sequences were circularized and annotated using Dfast [112] prokaryotic annotation pipeline. Pairwise alignments were performed using BLAST [106]. To assess the presence of hvKp and antibiotic resistance genes, reference sequences (S3 Table) were extracted, and BLAST [106] was used to align these reference sequences to *ter* encoding plasmid sequences. To study the genes conserved around the *ter* locus, gene level multiple sequence alignment (MSA) of the genes within 10 kbp upstream of the putative biosynthetic locus and downstream of the *ter* operon in all the plasmids. These loci were visualized by coding annotated genes using their 4-character gene names and unannotated hypothetical proteins using their gene cluster identifier as determined by CD-HIT software [113] for the MSA. An additional MSA was performed using MAFFT [114] in the L-INS-i mode and visualized the MSA using MSAviewer [115] to understand conserved genes around the *ter* locus. To predict protein structure and function of genes within and adjacent to the *ter* locus, *ter* encoding plasmids were annotated using the PATRIC RAST tool kit [116,117]. Unique annotation frequencies were calculated, and then unique predicted amino acid sequences were annotated predictively using I-TASSER [38–40]. S1 Table indicates reference amino acid sequences used for protein structure and function prediction. Only the highest scoring Predicted Biological Process, Predicted Molecular Function, and Predicted Pubchem Ligand Binding Site are reported. Finally, complete plasmid MSA was performed and visualized using Mauve (MegAlign Pro, DNASTAR Inc., Madison, WI). Illumina and Nanopore reads are available on the SRA in BioProject PRJNA464397.

### Murine models of infection

Six- to 12-week-old C57BL/6J male and female mice from barriers RB07 and RB16 (Jackson Laboratory, Jackson, ME) were used for all murine models of infection. Gender was evenly distributed in all groups. For bacteremia studies, WT NTUH-K2044 and NTUH-K2044Δ*terC* were cultured overnight in LB, then bacteria were pelleted, resuspended, mixed 1:1, diluted in sterile PBS to the appropriate dose, and mice were inoculated intraperitoneally with approximately 5×10^5^ CFU in 100 μL of PBS. After 24 hours, mice were euthanized by CO_2_ asphyxiation and blood, spleen, liver, and brain were collected. Solid organs were weighed and homogenized in sterile PBS, and whole blood and solid organ homogenates were plated on selective media. For oral inoculation studies, mice from both barriers were given regular drinking water, water containing 0.5 g/L ampicillin 3 days prior to inoculation and throughout the experiment, or water containing a SCFA cocktail (67.5 mM sodium acetate, 40 mM sodium butyrate, 25.9 sodium propionate) 7 days prior to inoculation and throughout the experiment. The SCFA dose and duration was chosen based on previous studies [64,118]. Antibiotic or SCFA-containing water was changed every 3 days. Kp strains were cultured overnight in LB in the presence of antibiotics when appropriate, then bacteria were pelleted, resuspended, mixed 1:1, diluted in sterile PBS to the appropriate dose, and mice were orally inoculated via oral gavage with approximately 5×10^6^ CFU in 100 μL of PBS. Single strain infections were performed as above without mixing the two strains. For four days post-inoculation, a fresh fecal pellet was collected from each mouse, weighed, and homogenized in sterile PBS, and homogenates were dilution plated on both LB agar containing 10 μg/ml ampicillin or 40 μg/ml kanamycin to determine Kp load. When complemented or empty vector control strains were used, plasmid maintenance was monitored by plating both on LB agar containing 10 μg/ml ampicillin or 40 μg/ml kanamycin to determine total Kp load, and on LB agar containing 10 μg/ml ampicillin and 80 μg/ml chloramphenicol or 40 μg/ml kanamycin and 80 μg/ml chloramphenicol to determine plasmid maintaining Kp load. In all models, mice were monitored daily for signs of distress (hunched posture, ruffled fur, decreased mobility, and dehydration) and euthanized at predetermined timepoints, or if signs of significant distress were displayed. No blinding was performed between experimental groups.

### 16S rRNA sequencing and analysis

Fecal DNA was isolated using the MagAttract PowerMicrobiome DNA/RNA Kit (Qiagen) and an epMotion 5075 liquid handling system. The V4 region of the 16S rRNA gene was amplified and sequenced as previously described [119]. Standard PCRs used 1, 2 or 7 μL of undiluted DNA and touchdown PCR used 7 μL of undiluted DNA. 16S rRNA gene sequence data was processed and analyzed using the software package mothur (v.1.40.2) [120,121]. Sequences were binned into OTUs based on 97% sequence similarity using the OptiClust method [122] following sequence processing and alignment to the SILVA reference alignment (release 128) [123]. θ_YC_ distances [47] were calculated between communities, and AMOVA [124] was used to determine statistically significant differences between experimental groups [47]. Principal coordinates analysis was used to visualize the θ_YC_ distances between samples. Taxonomic composition of the bacterial communities was assessed by classifying sequences within mothur using a modified version of the Ribosomal Database Project training set (version 16) [125,126], and diversity metrics, including inverse Simpson, were calculated. Finally, linear discriminant analysis effect size was used to determine if specific families and OTUs were differentially abundant in different groups [51]. Putative genus and species assignments were performed by comparing the representative 16S rRNA sequences from OTUs to the NCBI 16S ribosomal RNA sequence database. These assignments were confirmed using the Ribosomal Database Project (RDP) database, with the exception of OTUs assigned to *Muribaculum intestinale* based on NCBI, but to Porphyromonadaceae by RDP [125]. All other assignments were in agreement. 16S rRNA gene sequencing reads are available on the SRA in BioProject PRJNA464397.

### PICRUSt2 metagenome prediction

16S rRNA gene sequence data processed using the software package mothur (v.1.40.2) [120,121] was used for metagenome prediction analysis using the PICRUSt2 pipeline [59]. 16S rRNA gene sequences were aligned using HMMER (v.3.3, hmmer.org), placed in the default 16S rRNA gene reference tree, which is comprised of 20,000 16S rRNA gene sequences in the Integrated Microbial Genomes database [127], using EPA-NG [128], and then the complete tree was constructed with GAPPA [129]. Following tree construction, unknown lineages were inferred, and KEGG pathway copy number was predicted using castor [130]. Finally, metabolic pathway abundances were inferred from MetaCyc using MinPath [131]. For analysis, metabolic pathway abundances were rounded to the nearest whole number and normalized across each sample to determine the relative abundance of each gene family and inferred metabolic pathway. Principle coordinate analysis was performed in R (v.3.6.3) using the “ggplot2” and “ggfortify” packages to visualize differences in inferred metabolic pathway relative abundances between experimental groups. AMOVA P values [124] were calculated using the “vegan” package and used to determine statistically significant differences between experimental groups [47].

### SCFA growth and killing assays

SCFA containing LB was prepared by adding acetic acid, butyric acid, and/or propionic acid to LB at the appropriate concentration. The pH of SCFA-containing LB was adjusted to 7.5 or 5.75 with HCl or NaOH until the appropriate pH was achieved. Control LB lacking SCFAs was also pH adjusted to 7.5 or 5.75. Finally, pH adjusted media was sterile filtered using a 0.22 μM filter. For growth assays, Kp strains were cultured overnight at 37° C with aeration in LB in the presence of antibiotics when appropriate. Overnight cultures were diluted to an OD_600_ of 0.02 in pH adjusted LB, then mixed 1:1 with pH adjusted LB with or without 2X SCFAs, to achieve a final 1X dilution of SCFAs and OD_600_ of 0.01. For competitive growth assays, overnight cultures were mixed 1:1 before dilution. Cultures were incubated at 37° C with aeration and OD_600_ readings were taken every 15 min using an Eon microplate reader with Gen5 software (Version 2.0, BioTek, Winooski, VT) for 24 hours. Area under the curve was quantified using Prism 8 (GraphPad Software, La Jolla, CA). For killing assays, 1 mL of overnight culture was pelleted, resuspended in pH adjusted LB with or without SCFAs, and cultured at 37° C with aeration. Cultures were sampled immediately, then every 2 hours and dilution plated on LB agar to quantify bacterial viability.

### SCFA quantification

SCFAs were quantified as previously described [86]. Briefly, archived fecal pellets were suspended 1:2, 1:5, or 1:10 (weight:volume) in sterile PBS and homogenized. Homogenized samples were then centrifuged at 10,000 × *g* for 5 minutes to pellet the solid fraction and the supernatant was retained. The supernatant was then vacuum through a 0.22 μm filter prior to HPLC analysis. SCFA composition was measured using a Shimadzu HPLC (Shimadzu Scientific Instruments) equipped with an RID-10A refractive index detector. 30 μL injections were run on an Aminex HPX-87H column (Bio-Rad Laboratories, Hercules, CA) at 50° C with 0.01 H_2_SO_4_ mobile phase and a flow rate of 0.6 mL/minute. SCFA concentration was determined by interpolation from a 9-point standard curve containing acetate, butyrate, and propionate at concentrations between 0.1 mM to 40 mM, then normalized to dilution factor and tissue weight. Total SCFA concentration is a sum of acetate, butyrate, and propionate concentrations.

### Statistical analysis

For *in vitro* studies, two-tailed Student’s *t*-tests or ANOVA followed by indicated post-hoc test was used to determine significant differences between groups. All *in vitro* experimental replicates represent biological replicates. All animal experiments were repeated at least twice with independent bacterial cultures. Competitive indices ((CFU mutant output/CFU WT output)/(CFU mutant input/CFU WT input)) [132] were log transformed and a one-sample *t-*test was used to determine significant differences from a hypothetical value of 0 and paired ratio *t*-test, or two-tailed Student’s *t-*test as indicated in figure legends was used to determine significant differences between groups. A P value of less than 0.05 was considered statistically significant for all experiments, and analysis was performed using Prism 8 (GraphPad Software, La Jolla, CA) unless otherwise indicated.

## Supporting information

S1 Fig Confirmation of the *terC λ-*red mutation. The WT NTUH-K2044 and an isogenic Δ*terC* mutant clone (Kp2257) were sequenced to identify the position of the kanamycin resistance cassette (“KanR”, A) and ensure that no spurious mutations occurred during the generation of this isogenic mutant. (B) NTUH-K2044 containing an empty vector and the sequenced isogenic Δ*terC* mutant clone containing an empty vector or the pTerZ-F plasmid were grown on LB or LB containing 100 μM K_2_TeO_3_^-2^ to visualize inhibition of growth (dilution series 10^0^−10^−6^ of overnight culture). (C) NTUH-K2044 and the sequenced isogenic Δ*terC* mutant clone containing an empty vector or the pTerZ-F plasmid were mixed 1:1 and approximately 5x10^6^ CFU were orally gavaged into mice sourced from barrier RB16 (n = 6–7). A fresh fecal pellet was collected 24 hours after inoculation, CFUs were enumerated, and log competitive indices (mutant:WT) were calculated (median and IQR displayed, **P < 0.005, one-sample *t* test compared to a hypothetical value of 0 or Student’s *t* test). Each data point represents an individual animal.
(TIF)

S2 Fig Size of *ter*-encoding plasmids. The size and predicted number of coding sequences (CDS) was determined for plasmids encoding the *ter* operon from Martin *et al*. mSystems, 2018 (red) or reference strains from the NCBI database (blue). The pK2044 hvKp plasmid is shown in black.
(TIF)

S3 Fig *ter*-encoding plasmids display variable sequence similarity, gene arrangement and gene content. Pairwise sequence similarities were determined for plasmids from Martin *et al*. mSystems, 2018 (A), and visualized using Mauve (B). Pairwise sequence similarities were also determined for Kp reference plasmids from the NCBI database (C). For A and C, each row and column represent one plasmid. The Kp reference plasmid heat map is organized by pairwise similarity to the pK2044 hvKp plasmid.
(TIF)

S4 Fig *Klebsiella* sp. containing *terZ-F* are distributed globally and can be found in many different, unrelated environments. 14,060 high-quality *Klebsiella* sp. genomes, 1,989 of which contain *terZ-F*, and their corresponding metadata were extracted from the Pathosystems Resource Integration Center (PATRIC). For *terZ-F* containing genomes, country of isolation metadata was summarized by genome counts per country (A). 665 genomes did not have corresponding country of isolation metadata. Host species of origin metadata and corresponding environment of origin metadata of *terZ-F* containing genomes was also summarized (B). *Klebsiella* sp. isolation metadata was also compared between *Klebsiella* sp. genomes stratified by the presence of the *ter* operon. Metadata from 10,687 human-derived, 459 non-human derived, and 2,914 *Klebsiella* sp. of unknown origin isolates was compared between the source of isolation (C). Human-derived isolates were further stratified by the site of infection, and odds ratios were calculated between isolation site and the presence of *terZ-F* (D). The numbers in parentheses indicates the total number of isolates from that site and the numbers in the heat map boxes indicate the percent of isolates that contain or lack *terZ-F* at that site.
(TIF)

S5 Fig *terC* is dispensable during bacteremia. NTUH-K2044 and the isogenic Δ*terC* mutant (clone Kp2259) were mixed 1:1 and approximately 5x10^5^ CFU were inoculated into male and female C57BL6/J mice via peritoneal infection (n = 12). 24 hours post-inoculation, mice were euthanized, tissue CFUs were enumerated (A, mean displayed, *P < 0.05, unpaired t test), and log competitive indices (mutant:WT) were calculated (B, mean displayed, **P < 0.005, one-sample t test compared to a hypothetical value of 0). Each data point represents an individual animal.
(TIF)

S6 Fig Gut Kp load during competitive gut colonization. (A-D) Three days prior to inoculation, male and female C57BL6/J mice sourced from barriers RB16 and RB07 were treated with 0.5 g/L ampicillin or regular drinking water. NTUH-K2044 and the isogenic Δ*terC* mutant (clone Kp2259) were mixed 1:1 and approximately 5x10^6^ CFU were orally gavaged into mice (n = 9–18 per group). A fresh fecal pellet was collected daily from each animal and CFUs were enumerated (*P < 0.05, ratio paired t test).
(TIF)

S7 Fig *terC* is not a colonization factor during mono-strain gut colonization. Male and female C57BL6/J mice sourced from barriers RB16 (A) and RB07 (B) were orally gavaged with approximately 5x10^6^ CFU of NTUH-K2044 or the isogenic Δ*terC* mutant (clone Kp2259, n = 14–24 per group). A fresh fecal pellet was collected daily from each animal and CFUs were enumerated (geometric mean displayed). Each data point represents an individual animal.
(TIF)

S8 Fig Mouse survival during gut colonization. Survival of mice from male and female C57BL6/J barriers RB16 and RB07 (16–20 per group) following oral gavage with approximately 5x10^6^ CFU of a 1:1 mix of NTUH-K2044 and the isogenic Δ*terC* mutant (clone Kp2259, A). Survival of mice from male and female C57BL6/J barriers RB07 and RB16 (n = 14–24 per group) following oral gavage with approximately 5x10^6^ CFU of NTUH-K2044 or the isogenic Δ*terC* mutant (B). Data were analyzed by Mantel-Cox test between each treatment group in A and between each treatment group and between WT and *ΔterC* treated groups in B.
(TIF)

S9 Fig Community composition of mouse microbiota over time. (A-D) Fecal pellets collected daily from male and female C57BL6/J mice sourced from barriers RB16 and RB07 with or without three days treatment with 0.5 g/L ampicillin (n = 9–20 mice per group) following Kp inoculation were subjected to 16S rRNA gene sequencing. Average relative abundance values for bacterial families where relative abundance values are greater than 0.01 are displayed.
(TIF)

S10 Fig Differences in community composition between the microbiota of mice sourced from barriers RB16 and RB07 remain stable over time. Fecal pellets collected daily from male and female C57BL6/J mice sourced from barriers RB16 and RB07 (n = 16–18 mice per group) following Kp inoculation were subjected to 16S rRNA gene sequencing. Pairwise community dissimilarity values between the fecal microbiota communities were visualized by Principal coordinates analysis (groups compared by AMOVA). Each data point represents an individual animal.
(TIF)

S11 Fig Differences in community composition between the microbiota of mice sourced from barrier RB16 with or without antibiotic treatment remain stable over time. Fecal pellets collected daily from male and female C57BL6/J mice sourced from barrier RB16 with or without three days treatment with 0.5 g/L ampicillin (n = 10–18 mice per group) following Kp inoculation were subjected to 16S rRNA gene sequencing. Pairwise community dissimilarity values between the fecal microbiota communities were visualized by Principal coordinates analysis (groups compared by AMOVA). Each data point represents an individual animal.
(TIF)

S12 Fig Differences in community composition between the microbiota of mice sourced from barrier RB07 with or without antibiotic treatment remain stable over time. Fecal pellets collected daily from male and female C57BL6/J mice sourced from barrier RB07 with or without three days treatment with 0.5 g/L ampicillin (n = 9–16 mice per group) following Kp inoculation were subjected to 16S rRNA gene sequencing. Pairwise community dissimilarity values between the fecal microbiota communities were visualized by Principal coordinates analysis (groups compared by AMOVA). Each data point represents an individual animal.
(TIF)

S13 Fig Differences in community composition between the microbiota of mice sourced from barriers RB16 and RB07 with antibiotic treatment remain stable over time. Fecal pellets collected daily from male and female C57BL6/J mice sourced from barriers RB16 and RB07 with three days treatment with 0.5 g/L ampicillin (n = 9–10 mice per group) following Kp inoculation were subjected to 16S rRNA gene sequencing. Pairwise community dissimilarity values between the fecal microbiota communities were visualized by Principal coordinates analysis (groups compared by AMOVA). Each data point represents an individual animal.
(TIF)

S14 Fig Dissimilarity between the microbiota of mice sourced from barriers RB16 and RB07 remains stable over time. Fecal pellets collected daily from male and female C57BL6/J mice sourced from barriers RB16 and RB07 (n = 9–20 mice per group) following Kp inoculation were subjected to 16S rRNA gene sequencing. Pairwise community dissimilarity values between fecal microbiota communities were compared (**P < 0.005, ****P < 0.00005, one-way ANOVA followed by Tukey’s multiple comparisons post-hoc test). Each data point represents an individual comparison.
(TIF)

S15 Fig Differences in community diversity of mouse microbiota remain stable over time. Fecal pellets collected daily from male and female C57BL6/J mice sourced from barriers RB16 and RB07 with or without three days treatment with 0.5 g/L ampicillin (n = 9–20 mice per group) following Kp inoculation were subjected to 16S rRNA gene sequencing. Diversity of the fecal microbiota was summarized by inverse Simpson index (*P < 0.05, **P < 0.005, ****P < 0.00005, one-way ANOVA followed by Tukey’s multiple comparisons post-hoc test). Each data point represents an individual animal. RB16 +Abx is displayed in blue, and RB07 +Abx is displayed in orange.
(TIF)

S16 Fig Bacterial families that differentiate the microbiota of mice sourced from RB16 and RB07 over time. Fecal pellets collected daily from male and female C57BL6/J mice sourced from barriers RB16 and RB07 (n = 16–18 mice per group) following Kp inoculation were subjected to 16S rRNA gene sequencing. LEfSe was used to determine if specific bacterial families were differentially abundant between the fecal microbiota of RB16 and RB07 (Families with LDA ≥ 3.5 and P < 0.05 are shown).
(TIF)

S17 Fig Differences in relative abundance of bacterial families that differentiate the microbiota of mice sourced from RB16 and RB07 over time. Fecal pellets collected daily from male and female C57BL6/J mice sourced from barriers RB16 and RB07 (n = 16–18 mice per group) following Kp inoculation were subjected to 16S rRNA gene sequencing. Relative abundance of specific bacterial families that were differentially abundant between the fecal microbiota of RB16 and RB07 by LEfSe are displayed (*P < 0.05, **P < 0.005, ***P < 0.0005, ****P < 0.00005, Student’s *t* test).
(TIF)

S18 Fig OTUs that differentiate the microbiota of mice sourced from RB16 and RB07 remain stable over time. Fecal pellets collected daily from male and female C57BL6/J mice sourced from barriers RB16 and RB07 (n = 16–18 mice per group) following Kp inoculation were subjected to 16S rRNA gene sequencing. LEfSe was used to determine if specific OTUs were differentially abundant between the fecal microbiota of RB16 and RB07 (OTUs with LDA ≥ 3.5 and P < 0.05 are shown).
(TIF)

S19 Fig Differences in relative abundance of OTUs that differentiate the microbiota of mice sourced from RB16 and RB07 remain stable over time. Fecal pellets collected daily from male and female C57BL6/J mice sourced from barriers RB16 and RB07 (n = 16–18 mice per group) following Kp inoculation were subjected to 16S rRNA gene sequencing. Relative abundance of specific OTUs that were differentially abundant between the fecal microbiota of RB16 and RB07 by LEfSe are displayed (*P < 0.05, **P < 0.005, ***P < 0.0005, ****P < 0.00005, Student’s *t* test).
(TIF)

S20 Fig Bacterial families that differentiate the microbiota of mice sourced from RB16 from RB07 are sensitive to antibiotic treatment. Fecal pellets collected daily from male and female C57BL6/J mice sourced from barriers RB16 and RB16+Abx (n = 10–18 mice per group) following Kp inoculation were subjected to 16S rRNA gene sequencing. Relative abundance of specific bacterial families that were differentially abundant between the fecal microbiota of RB16 and RB07 by LEfSe are displayed (**P < 0.005, ***P < 0.0005, ****P < 0.00005, Student’s *t* test).
(TIF)

S21 Fig OTUs that differentiate the microbiota of mice sourced from RB16 from RB07 are sensitive to antibiotic treatment. Fecal pellets collected daily from male and female C57BL6/J mice sourced from barriers RB16 and RB16+Abx (n = 10–18 mice per group) following Kp inoculation were subjected to 16S rRNA gene sequencing. Relative abundance of specific OTUs that were differentially abundant between the fecal microbiota of RB16 and RB07 by LEfSe are displayed (*P < 0.05, **P < 0.005, ***P < 0.0005, ****P < 0.00005, Student’s *t* test).
(TIF)

S22 Fig Impact of Kp inoculation on the stability of microbiota of mice sourced from RB16 and RB07. Family (A) and OTU (B) relative abundance values pre- (Day 0) and post-Kp inoculation (Day 1) were subtracted to determine the impact of Kp inoculation on the fecal microbiota communities of barriers RB16 and RB07. Only significant differential relative abundance values are displayed (median and IQR displayed, one-sample t test compared to a hypothetical value of 0). Each data point represents an individual animal. Community dissimilarity between pre- (Day 0) and post-Kp inoculation was tested by AMOVA for each day following Kp inoculation (C). Each cell contains the AMOVA P value for the indicated comparison.
(TIF)

S23 Fig Differences in PICRUSt2 metagenome metabolic pathway predictions over time. Fecal pellets collected daily from male and female C57BL6/J mice sourced from barriers RB16 and RB07 (n = 16–18 mice per group) following Kp inoculation were subjected to 16S rRNA gene sequencing and analyzed using PICRUSt2. Predicted metabolic pathway relative abundance values were visualized by Principal coordinates analysis and dissimilarity was tested by AMOVA. Each data point represents an individual animal.
(TIF)

S24 Fig Short-chain fatty acid metabolic pathways differentiate the predicted metagenomes of the microbiota of mice sourced from RB16 and RB07 over time. Fecal pellets collected daily from male and female C57BL6/J mice sourced from barriers RB16 and RB07 (n = 16–18 mice per group) following Kp inoculation were subjected to 16S rRNA gene sequencing and analyzed using PICRUSt2. LEfSe was used to determine if predicted fermentation to short-chain fatty acid metabolic pathways were differentially abundant between the predicted metagenome of RB16 and RB07. Note that no predicted short-chain fatty acid metabolic pathways are enriched in the gut microbiota of RB07 following day 0.
(TIF)

S25 Fig Differences in relative abundance of predicted short-chain fatty acid metabolic pathways that differentiate the predicted metagenomes of the microbiota of mice sourced from RB16 and RB07 over time. Fecal pellets collected daily from male and female C57BL6/J mice sourced from barriers RB16 and RB07 (n = 16–18 mice per group) following Kp inoculation were subjected to 16S rRNA gene sequencing and analyzed using PICRUSt2. Relative abundance of specific PICRUSt2 predicted fermentation to short-chain fatty acid pathways that were differentially abundant between RB16 and RB07 by LEfSe are displayed (*P < 0.05, **P < 0.005, ***P < 0.0005, ****P < 0.00005, Student’s *t* test).
(TIF)

S26 Fig Antibiotic treatment reduces the relative abundance of PICRUSt2 predicted fermentation to short-chain fatty acid metabolic pathways that differentiate the microbiota of mice sourced from RB16 from RB07. Fecal pellets collected daily from male and female C57BL6/J mice sourced from barriers RB16 with or without three days treatment with 0.5 g/L ampicillin (n = 10–18 mice per group) following Kp inoculation were subjected to 16S rRNA gene sequencing and analyzed using PICRUSt2. Relative abundance of specific PICRUSt2 predicted fermentation to short-chain fatty acid pathways that were differentially abundant between RB16 and RB07 by LEfSe are displayed (*P < 0.05, **P < 0.005, ***P < 0.0005, ****P < 0.00005, Student’s *t* test).
(TIF)

S27 Fig Short-chain fatty acids (SCFAs) do not inhibit Kp growth or kill Kp in a *ter*-dependent manner. WT NTUH-K2044 and the isogenic Δ*terC* mutant (clone Kp2257) were grown in neutral (pH = 7.5) or acidic (pH = 5.75) conditions in the absence or presence of various SCFAs in combination or individually (A-B). Area under the curve (AUC) was calculated from growth curves (B, ****P < 0.00005 compared to no SCFA control, Tukey’s multiple comparison test following Two-way ANOVA, mean displayed ± SEM). WT NTUH-K2044, the isogenic Δ*terC* mutant, and the complement strain were grown in neutral (pH = 7.5) or acidic (pH = 5.75) conditions in the presence of increasing dilutions of SCFAs, and AUC was calculated (C). To assess inter-strain antagonism in a competitive growth assay, WT NTUH-K2044 and the isogenic Δ*terC* mutant were mixed 1:1 at an OD_600_ = 0.01 and exposed to indicated dilutions of SCFAs under acidic conditions (pH = 5.75) for 24 hours. After 24 hours, bacteria were dilution plated on selective media and log competitive indices (mutant:WT) were calculated (D). Stationary phase WT NTUH-K2044 and the isogenic Δ*terC* mutant were exposed to SCFAs under acidic conditions (pH = 5.75) to assess bacterial killing (E).
(TIF)

S28 Fig SCFA concentrations in fecal pellets of mice sourced from RB16 from RB07 with or without exogenous SCFA administration. Male and female C57BL6/J mice sourced from barriers RB16 and RB07 were treated with a SCFA cocktail or regular drinking water (sham) for 7 days. Total SCFAs (A), acetate (B), butyrate (C), and propionate (D) were quantified from fecal pellets (mean displayed, LSD test following one-way ANOVA).
(TIF)

S29 Fig SCFA concentrations in fecal pellets of mice sourced from RB16 with or without antibiotic treatment. Male and female C57BL6/J mice sourced from barrier RB16 were treated with or without antibiotics = for 3 days. Total SCFAs (A), acetate (B), butyrate (C), and propionate (D) were quantified from fecal pellets (mean displayed, *P < 0.05, **P < 0.005, ***P < 0.0005, Student’s *t* test).
(TIF)

S1 Table Conservation and function of *ter* locus and neighboring genes. (XLSX)

S2 Table *ter* plasmids and hvKp and antibiotic resistance gene characterization. (XLSX)

S3 Table hvKp and antibiotic resistance reference sequences. (XLSX)

S4 Table LDA analysis of MetaCyc pathways. (XLSX)

S5 Table Primers used in this study. (XLSX)

S1 Data *Klebsiella* sp. genome metadata summary. (XLSX)

S2 Data 16S rRNA gene sequencing summary. (XLSX)

S3 Data PICRUSt2 data summary. (XLSX)

S4 Data Experimental data. (XLSX)
