# Supplementary figures and images for: A plasmid locus associated with Klebsiella clinical infections encodes a microbiome-dependent gut fitness factor
Source: PLoS Pathog. 2021 Apr 30;17(4):e1009537. doi: 10.1371/journal.ppat.1009537 (PMC8115787; doi:10.1371/journal.ppat.1009537)

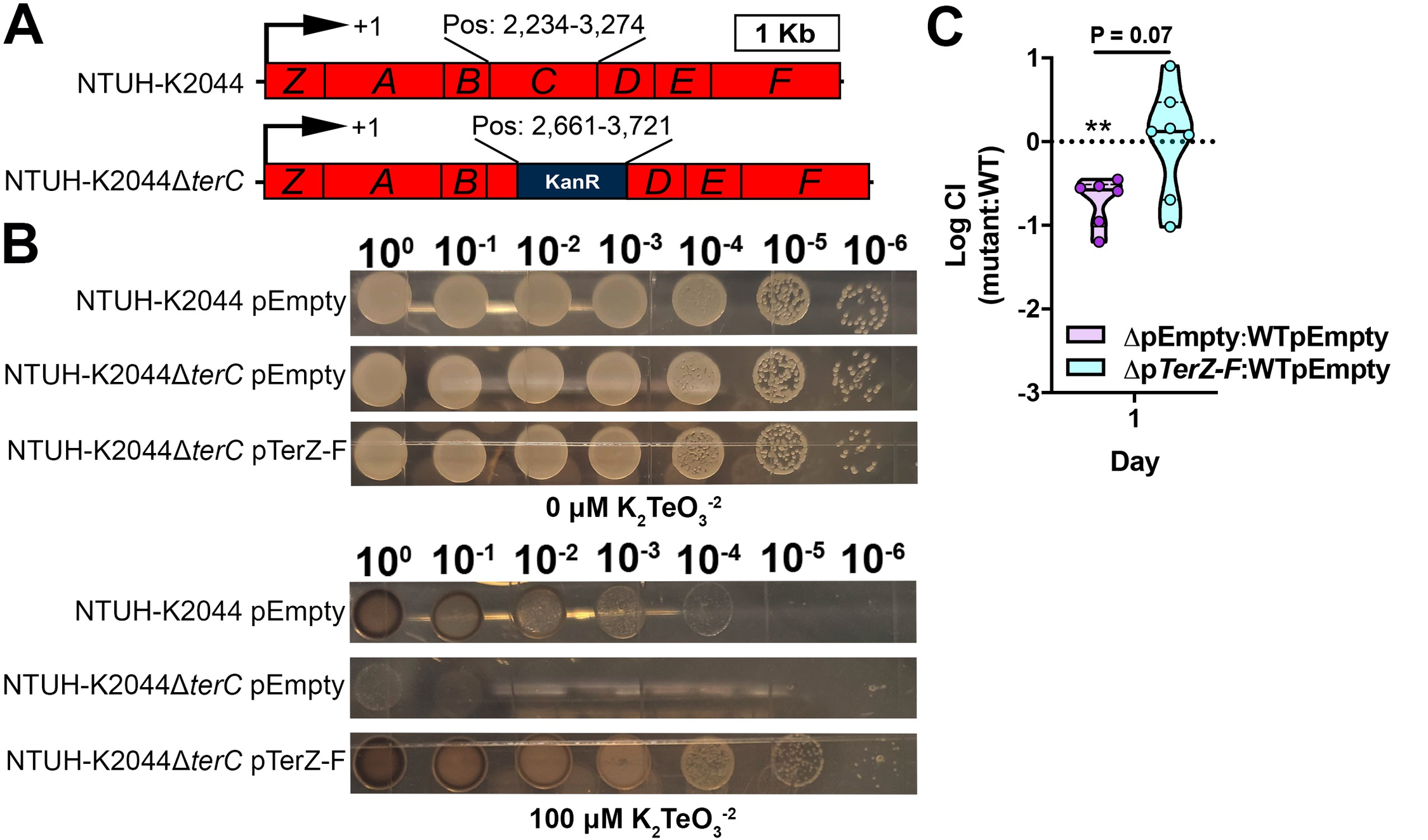

Supplement: S1 Fig — The WT NTUH-K2044 and an isogenic ΔterC mutant clone (Kp2257) were sequenced to identify the position of the kanamycin resistance cassette (“KanR”, A) and ensure that no spurious mutations occurred during the generation of this isogenic mutant. (B) NTUH-K2044 containing an empty vector and the sequenced isogenic ΔterC mutant clone containing an empty vector or the pTerZ-F plasmid were grown on LB or LB containing 100 μM K2TeO3-2 to visualize inhibition of growth (dilution series 100−10−6 of overnight culture). (C) NTUH-K2044 and the sequenced isogenic ΔterC mutant clone containing an empty vector or the pTerZ-F plasmid were mixed 1:1 and approximately 5x10^6^ CFU were orally gavaged into mice sourced from barrier RB16 (n = 6–7). A fresh fecal pellet was collected 24 hours after inoculation, CFUs were enumerated, and log competitive indices (mutant:WT) were calculated (median and IQR displayed, **P < 0.005, one-sample t test compared to a hypothetical value of 0 or Student’s t test). Each data point represents an individual animal. (TIF) [file ppat.1009537.s001.tif]

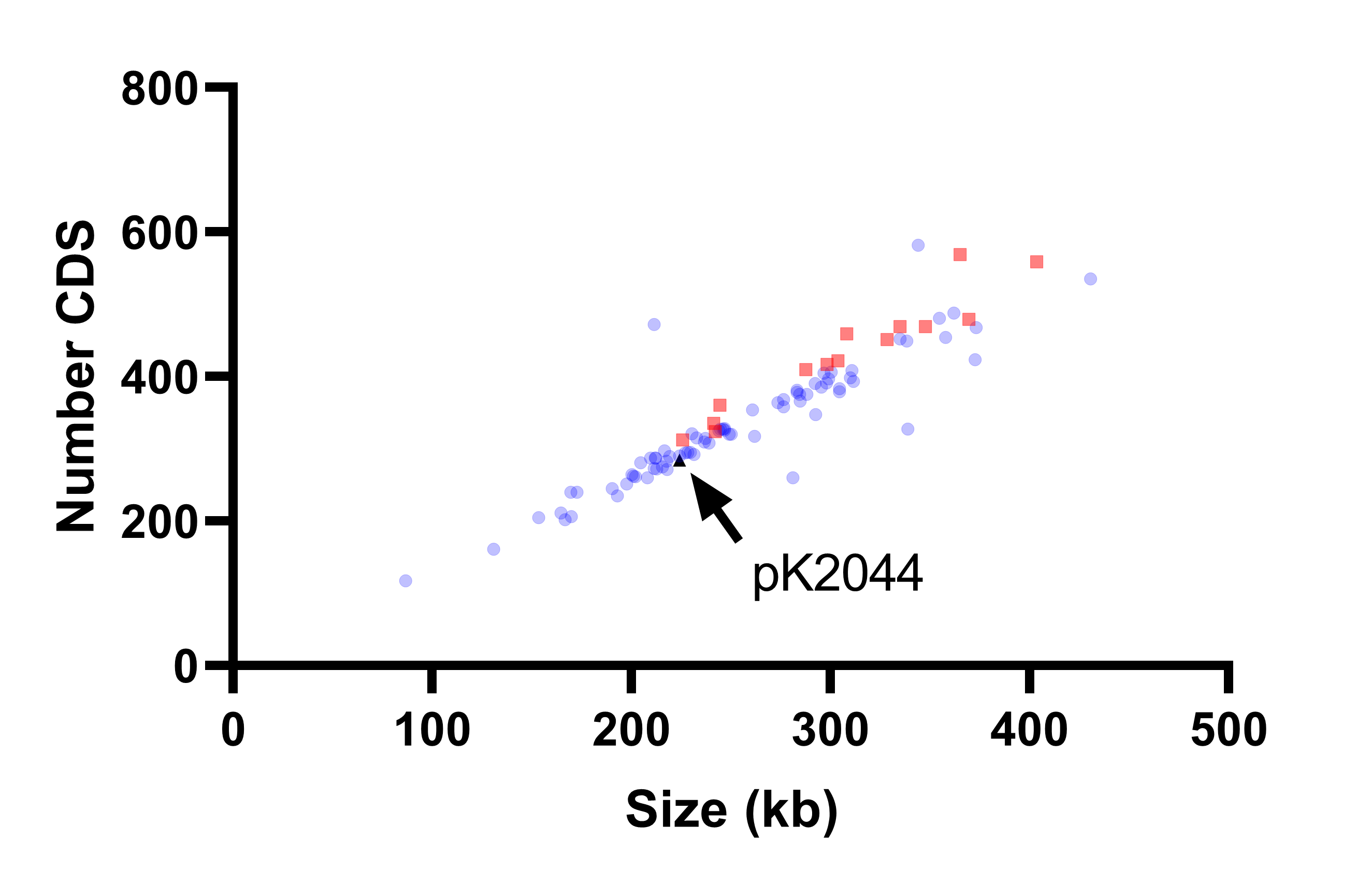

Supplement: S2 Fig — The size and predicted number of coding sequences (CDS) was determined for plasmids encoding the ter operon from Martin et al. mSystems, 2018 (red) or reference strains from the NCBI database (blue). The pK2044 hvKp plasmid is shown in black. (TIF) [file ppat.1009537.s002.tif]

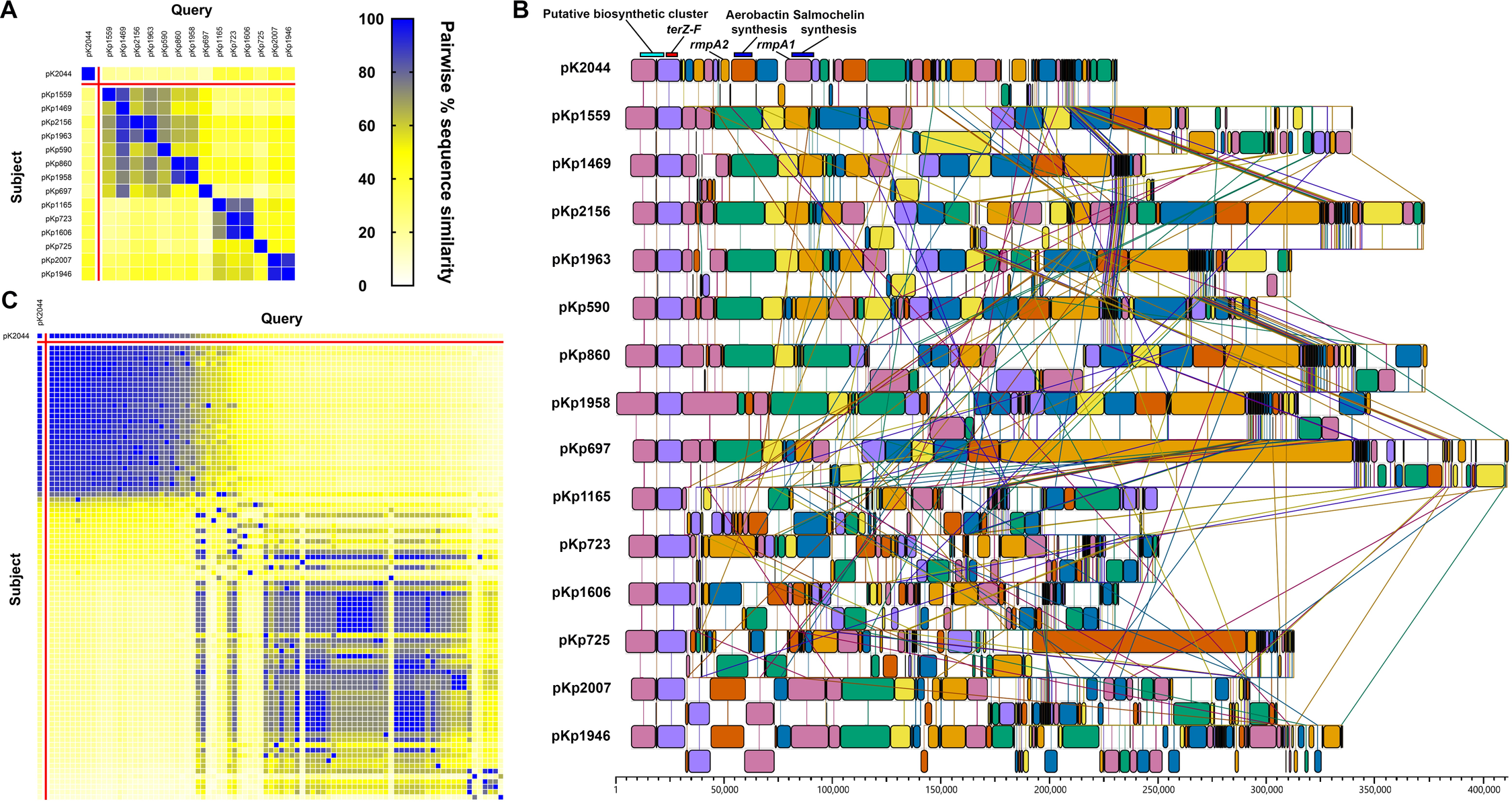

Supplement: S3 Fig — Pairwise sequence similarities were determined for plasmids from Martin et al. mSystems, 2018 (A), and visualized using Mauve (B). Pairwise sequence similarities were also determined for Kp reference plasmids from the NCBI database (C). For A and C, each row and column represent one plasmid. The Kp reference plasmid heat map is organized by pairwise similarity to the pK2044 hvKp plasmid. (TIF) [file ppat.1009537.s003.tif]

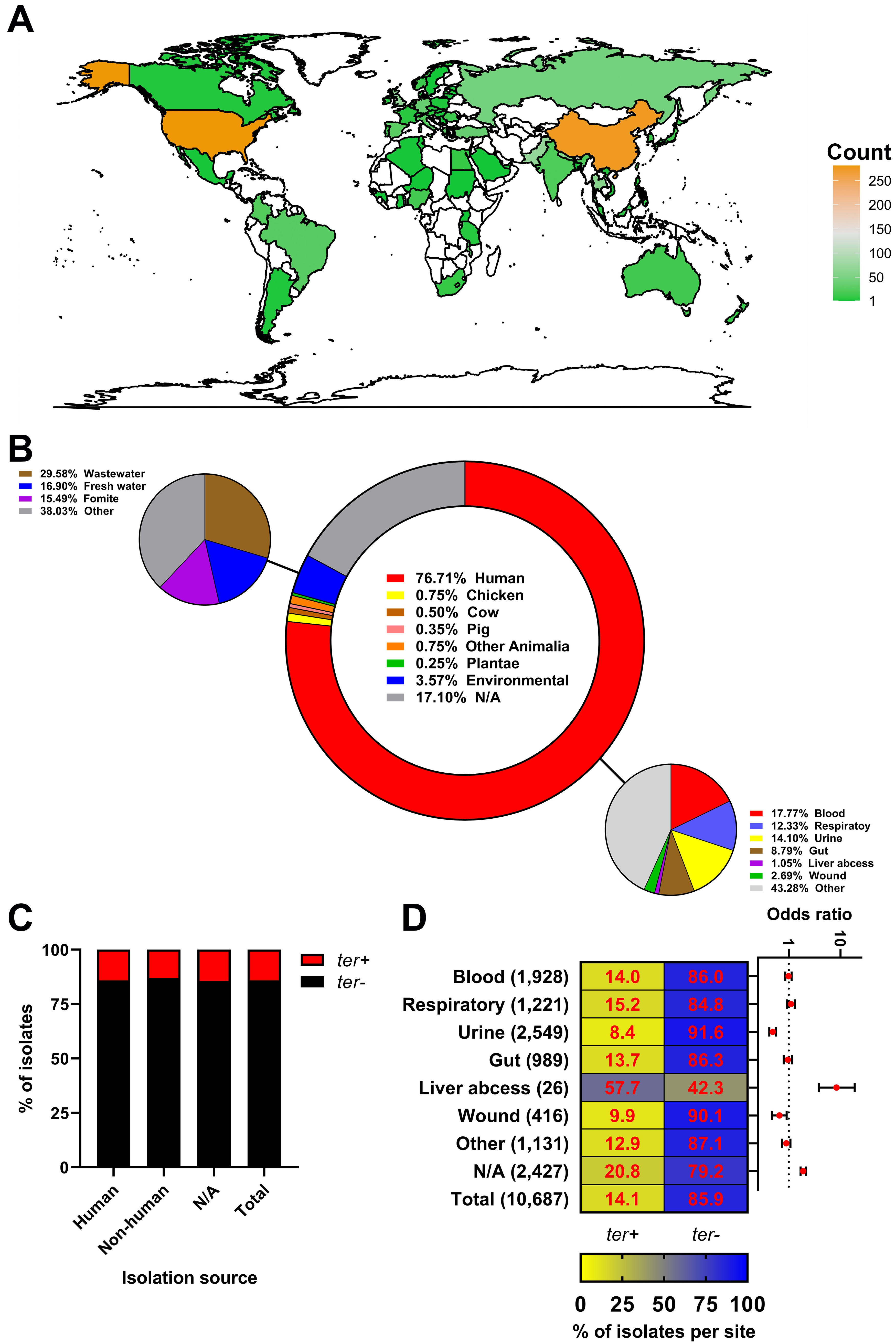

Supplement: S4 Fig — 14,060 high-quality Klebsiella sp. genomes, 1,989 of which contain terZ-F, and their corresponding metadata were extracted from the Pathosystems Resource Integration Center (PATRIC). For terZ-F containing genomes, country of isolation metadata was summarized by genome counts per country (A). 665 genomes did not have corresponding country of isolation metadata. Host species of origin metadata and corresponding environment of origin metadata of terZ-F containing genomes was also summarized (B). Klebsiella sp. isolation metadata was also compared between Klebsiella sp. genomes stratified by the presence of the ter operon. Metadata from 10,687 human-derived, 459 non-human derived, and 2,914 Klebsiella sp. of unknown origin isolates was compared between the source of isolation (C). Human-derived isolates were further stratified by the site of infection, and odds ratios were calculated between isolation site and the presence of terZ-F (D). The numbers in parentheses indicates the total number of isolates from that site and the numbers in the heat map boxes indicate the percent of isolates that contain or lack terZ-F at that site. (TIF) [file ppat.1009537.s004.tif]

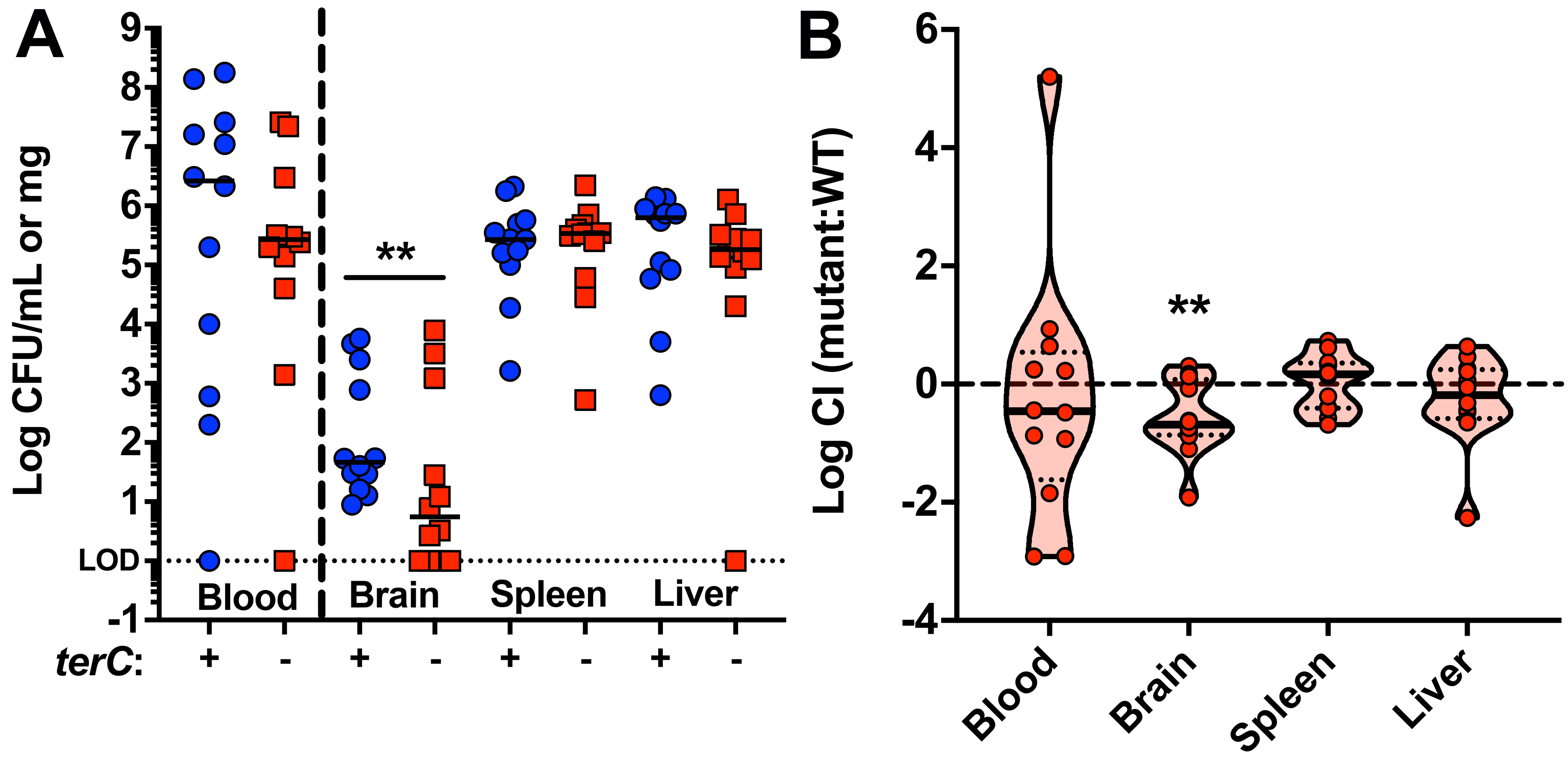

Supplement: S5 Fig — NTUH-K2044 and the isogenic ΔterC mutant (clone Kp2259) were mixed 1:1 and approximately 5x10^5^ CFU were inoculated into male and female C57BL6/J mice via peritoneal infection (n = 12). 24 hours post-inoculation, mice were euthanized, tissue CFUs were enumerated (A, mean displayed, *P < 0.05, unpaired t test), and log competitive indices (mutant:WT) were calculated (B, mean displayed, **P < 0.005, one-sample t test compared to a hypothetical value of 0). Each data point represents an individual animal. (TIF) [file ppat.1009537.s005.tif]

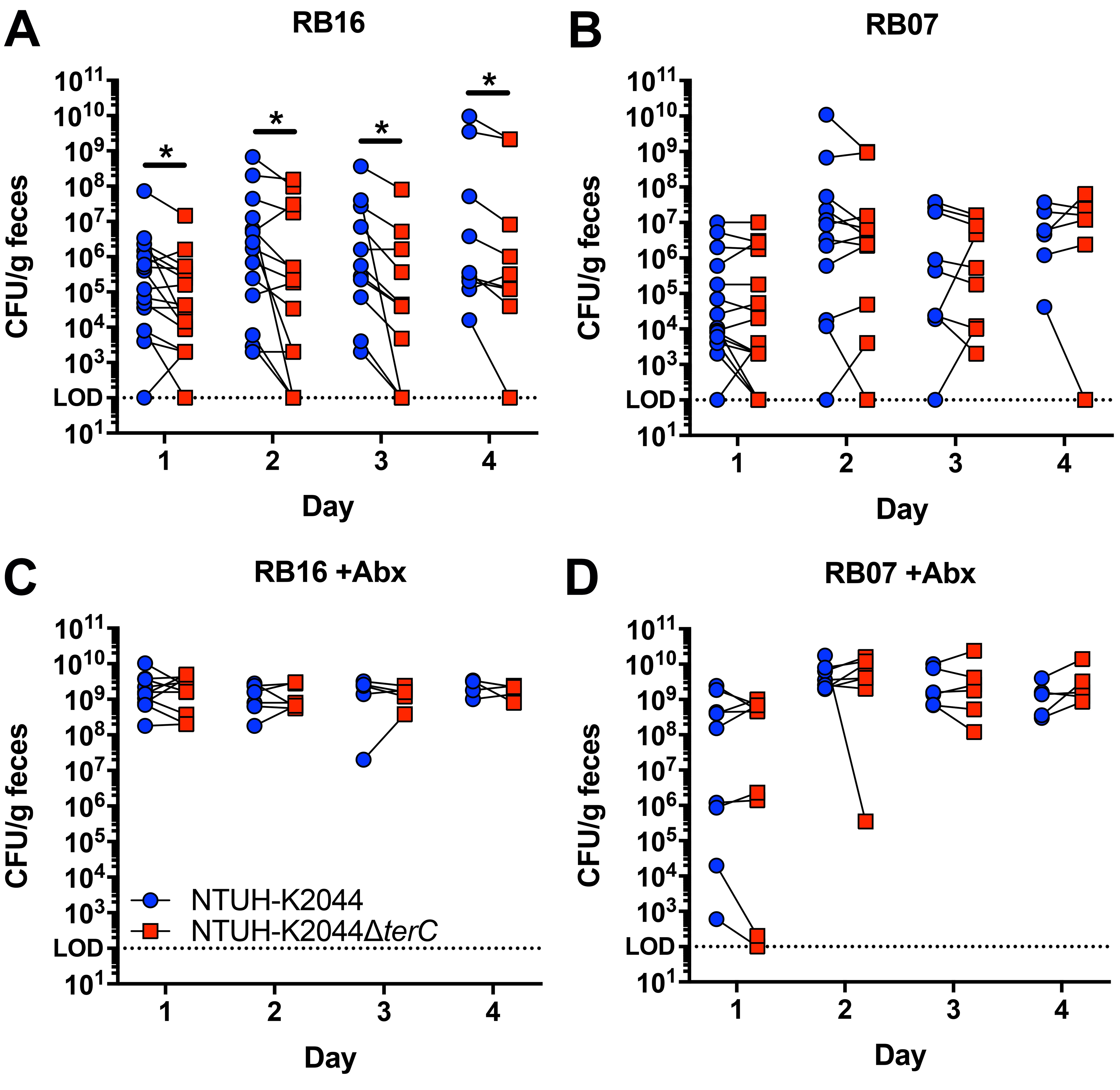

Supplement: S6 Fig — (A-D) Three days prior to inoculation, male and female C57BL6/J mice sourced from barriers RB16 and RB07 were treated with 0.5 g/L ampicillin or regular drinking water. NTUH-K2044 and the isogenic ΔterC mutant (clone Kp2259) were mixed 1:1 and approximately 5x10^6^ CFU were orally gavaged into mice (n = 9–18 per group). A fresh fecal pellet was collected daily from each animal and CFUs were enumerated (*P < 0.05, ratio paired t test). (TIF) [file ppat.1009537.s006.tif]

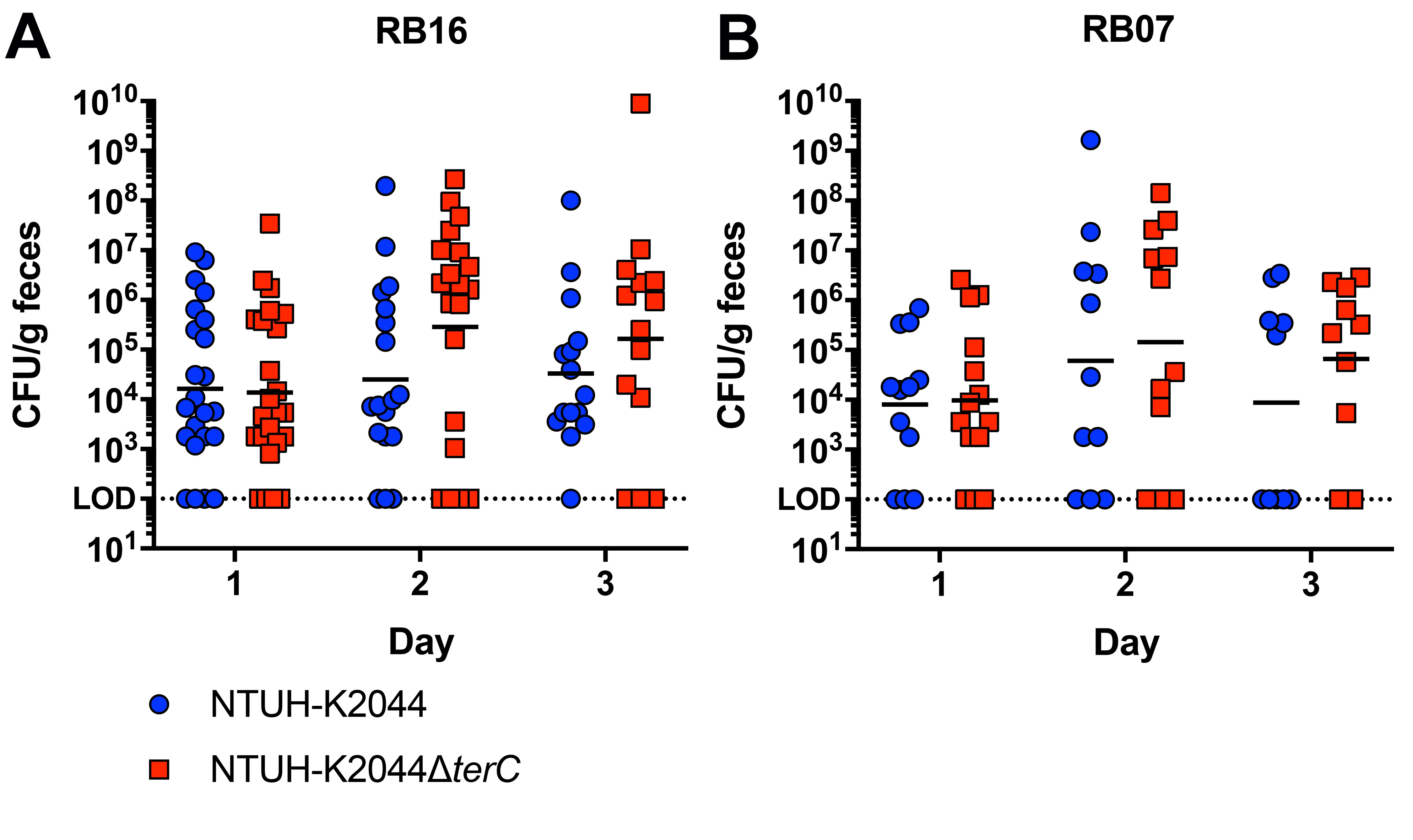

Supplement: S7 Fig — Male and female C57BL6/J mice sourced from barriers RB16 (A) and RB07 (B) were orally gavaged with approximately 5x10^6^ CFU of NTUH-K2044 or the isogenic ΔterC mutant (clone Kp2259, n = 14–24 per group). A fresh fecal pellet was collected daily from each animal and CFUs were enumerated (geometric mean displayed). Each data point represents an individual animal. (TIF) [file ppat.1009537.s007.tif]

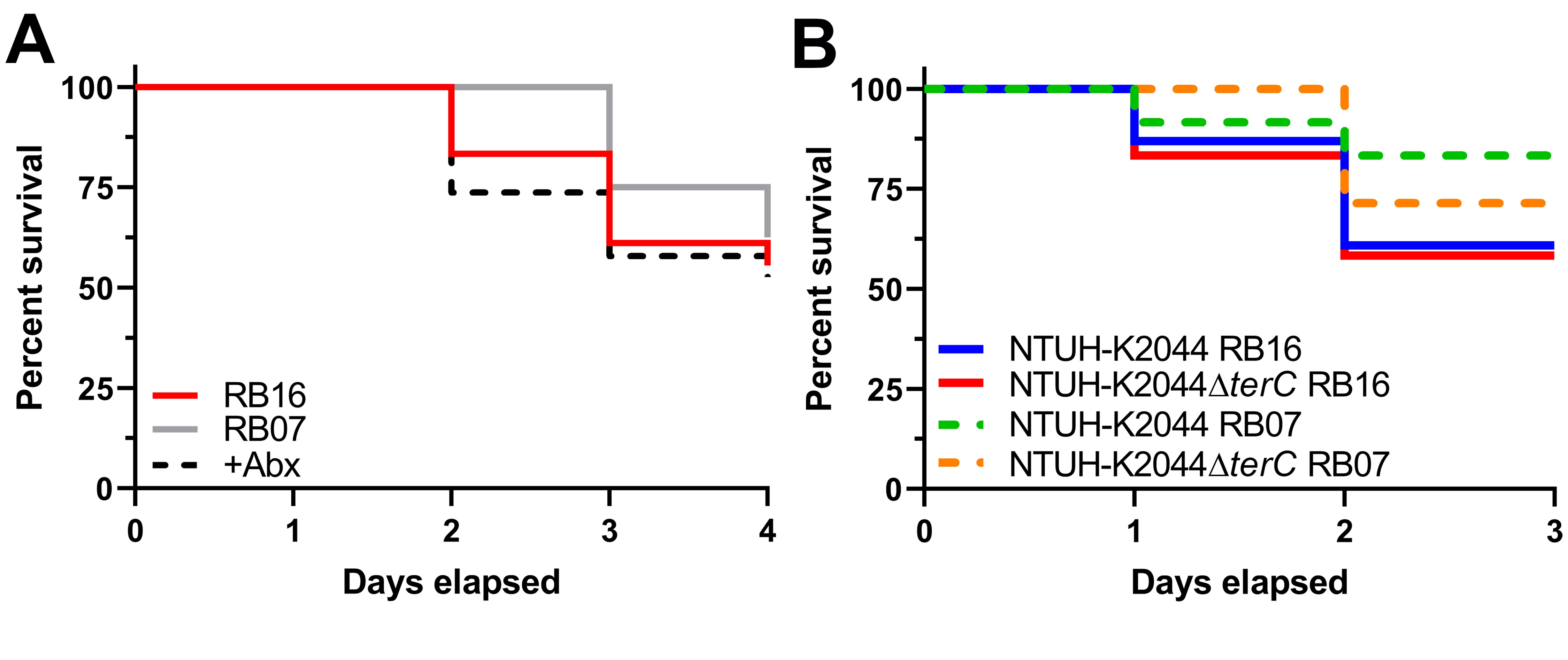

Supplement: S8 Fig — Survival of mice from male and female C57BL6/J barriers RB16 and RB07 (16–20 per group) following oral gavage with approximately 5x10^6^ CFU of a 1:1 mix of NTUH-K2044 and the isogenic ΔterC mutant (clone Kp2259, A). Survival of mice from male and female C57BL6/J barriers RB07 and RB16 (n = 14–24 per group) following oral gavage with approximately 5x10^6^ CFU of NTUH-K2044 or the isogenic ΔterC mutant (B). Data were analyzed by Mantel-Cox test between each treatment group in A and between each treatment group and between WT and ΔterC treated groups in B. (TIF) [file ppat.1009537.s008.tif]

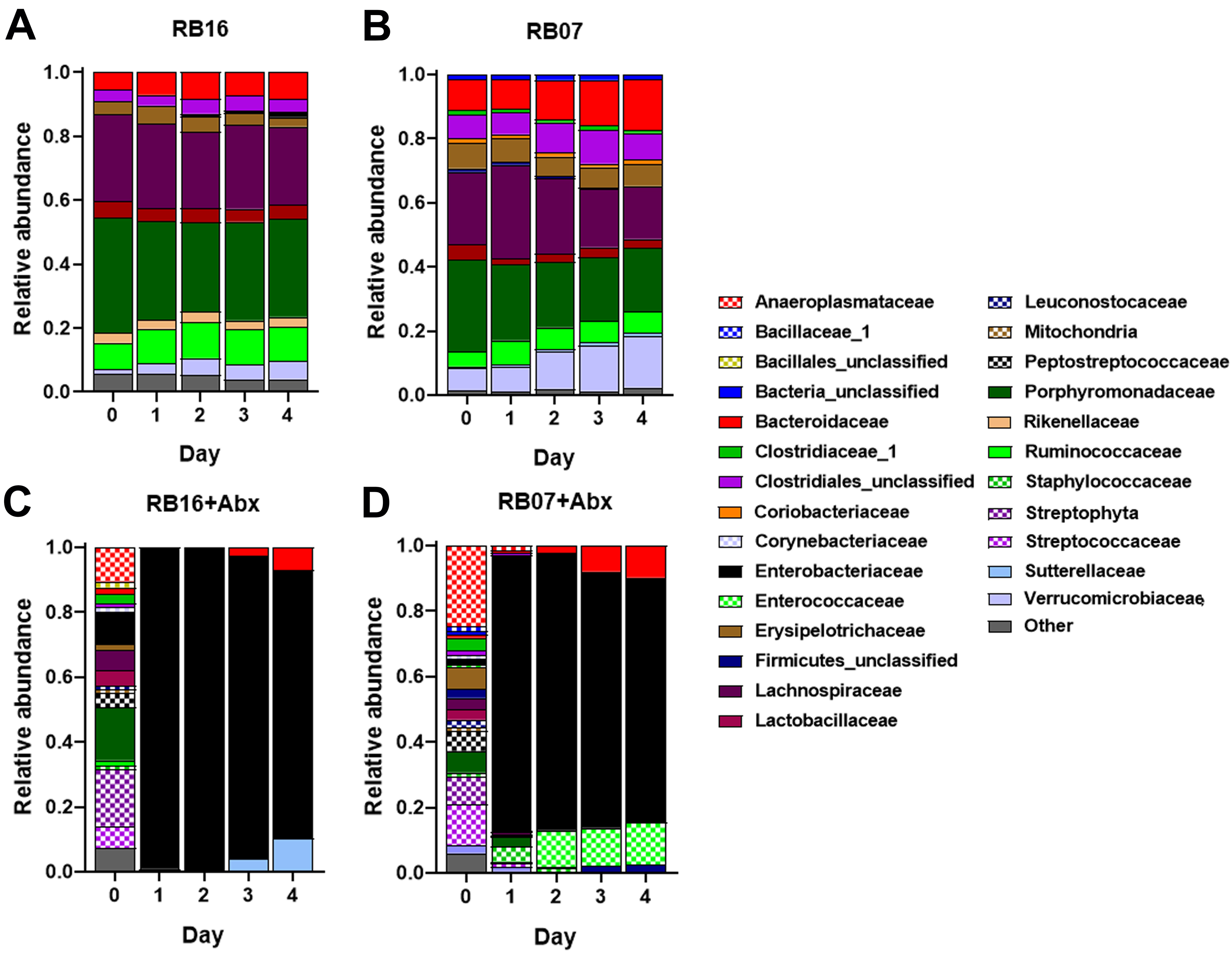

Supplement: S9 Fig — (A-D) Fecal pellets collected daily from male and female C57BL6/J mice sourced from barriers RB16 and RB07 with or without three days treatment with 0.5 g/L ampicillin (n = 9–20 mice per group) following Kp inoculation were subjected to 16S rRNA gene sequencing. Average relative abundance values for bacterial families where relative abundance values are greater than 0.01 are displayed. (TIF) [file ppat.1009537.s009.tif]

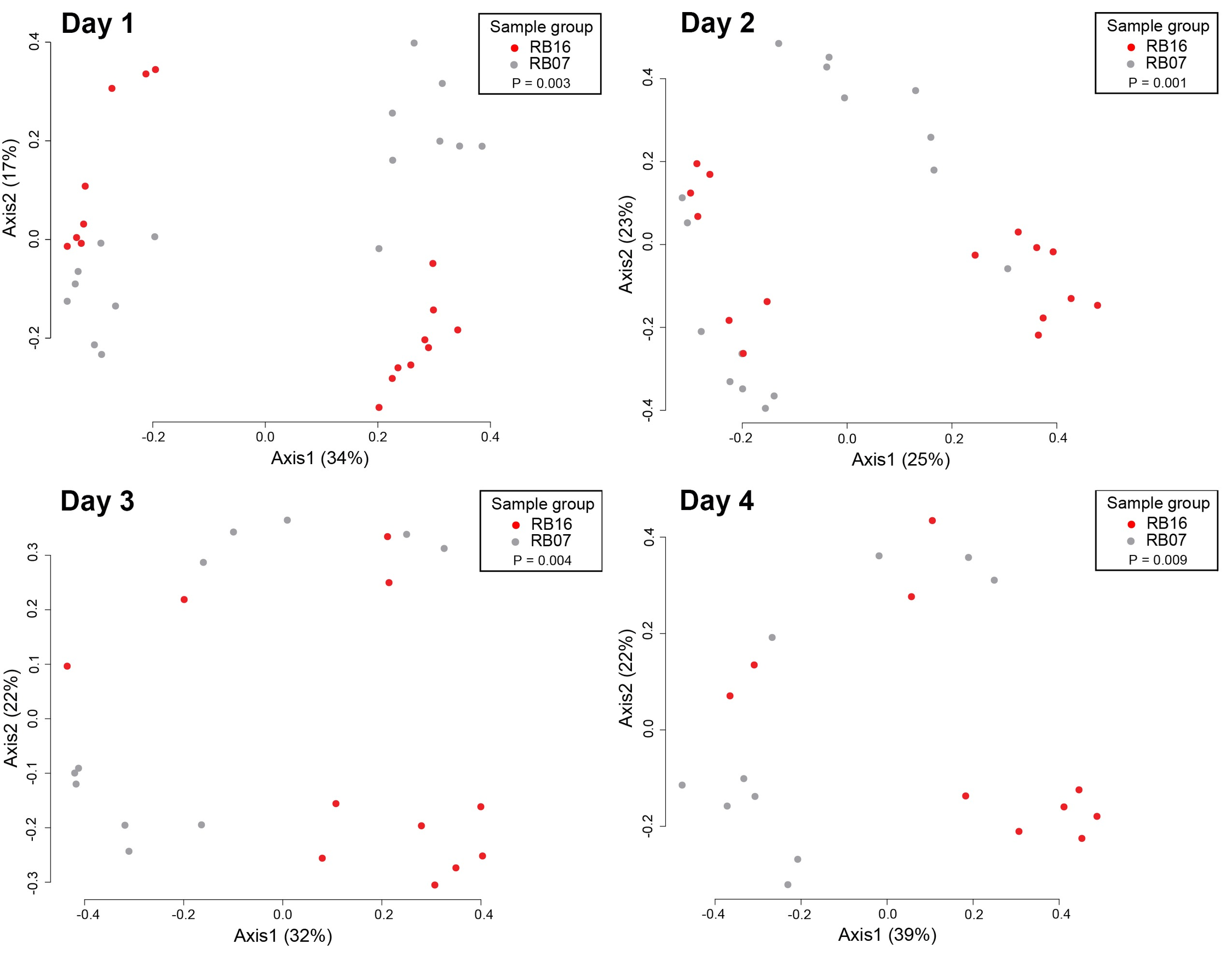

Supplement: S10 Fig — Fecal pellets collected daily from male and female C57BL6/J mice sourced from barriers RB16 and RB07 (n = 16–18 mice per group) following Kp inoculation were subjected to 16S rRNA gene sequencing. Pairwise community dissimilarity values between the fecal microbiota communities were visualized by Principal coordinates analysis (groups compared by AMOVA). Each data point represents an individual animal. (TIF) [file ppat.1009537.s010.tif]

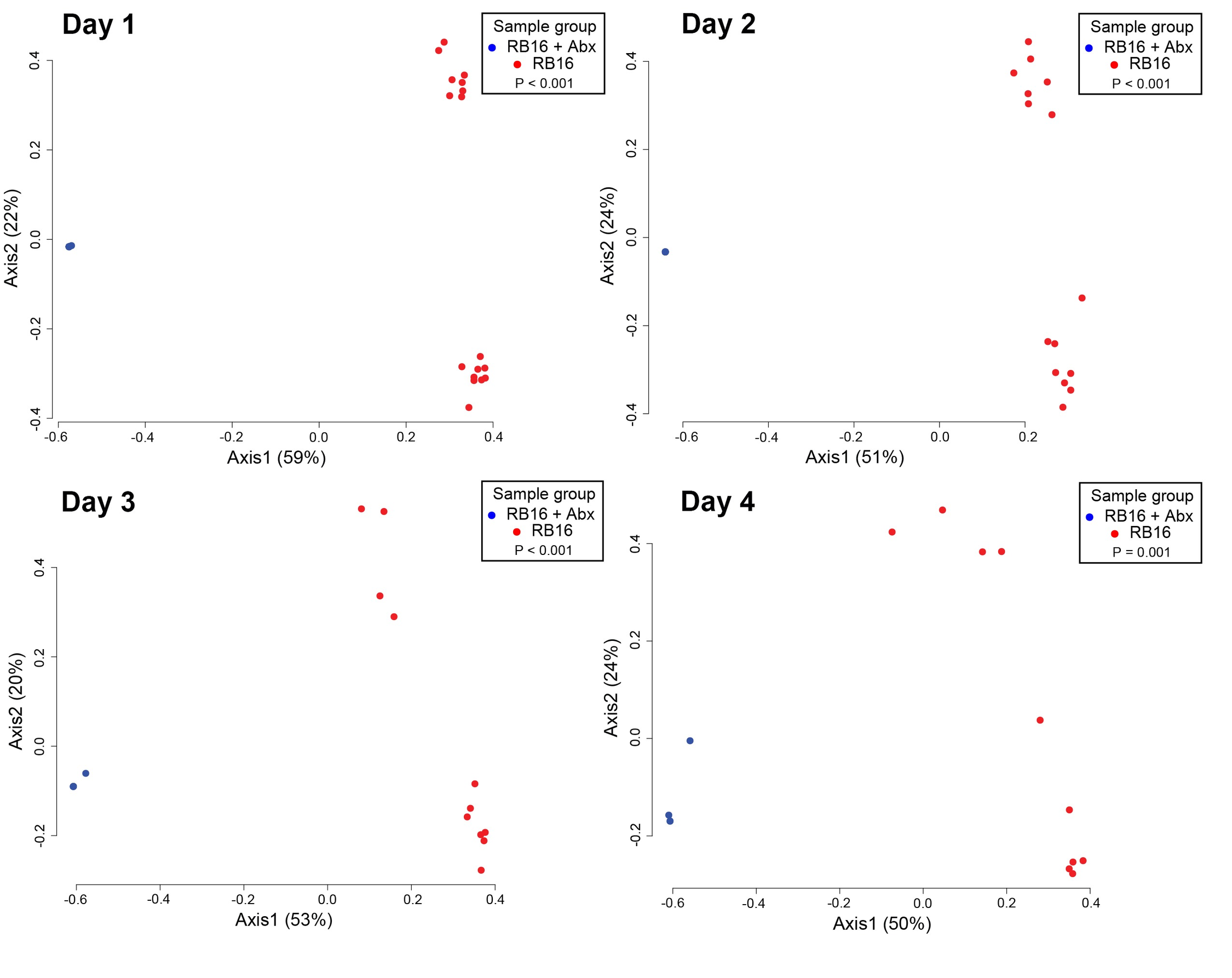

Supplement: S11 Fig — Fecal pellets collected daily from male and female C57BL6/J mice sourced from barrier RB16 with or without three days treatment with 0.5 g/L ampicillin (n = 10–18 mice per group) following Kp inoculation were subjected to 16S rRNA gene sequencing. Pairwise community dissimilarity values between the fecal microbiota communities were visualized by Principal coordinates analysis (groups compared by AMOVA). Each data point represents an individual animal. (TIF) [file ppat.1009537.s011.tif]

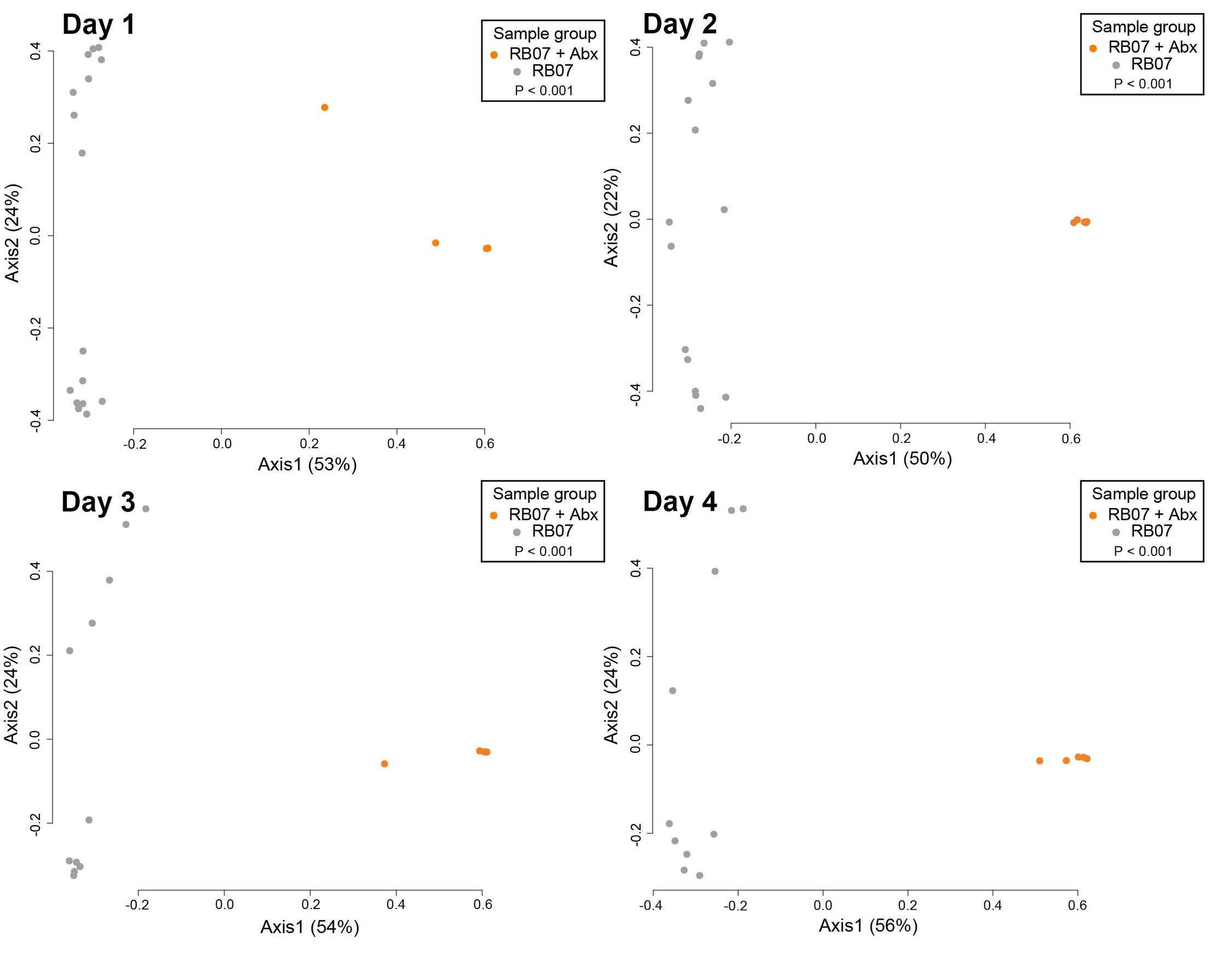

Supplement: S12 Fig — Fecal pellets collected daily from male and female C57BL6/J mice sourced from barrier RB07 with or without three days treatment with 0.5 g/L ampicillin (n = 9–16 mice per group) following Kp inoculation were subjected to 16S rRNA gene sequencing. Pairwise community dissimilarity values between the fecal microbiota communities were visualized by Principal coordinates analysis (groups compared by AMOVA). Each data point represents an individual animal. (TIF) [file ppat.1009537.s012.tif]

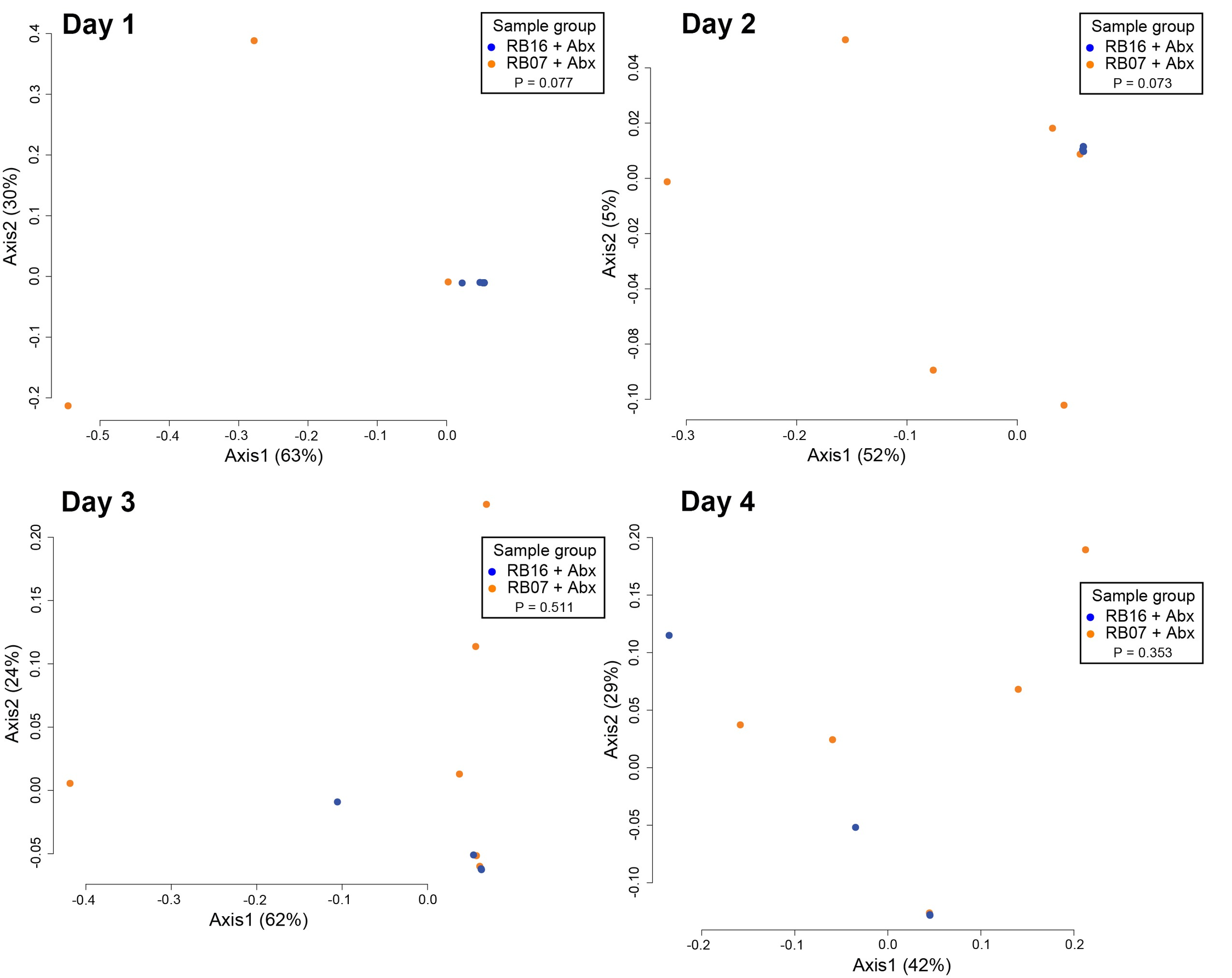

Supplement: S13 Fig — Fecal pellets collected daily from male and female C57BL6/J mice sourced from barriers RB16 and RB07 with three days treatment with 0.5 g/L ampicillin (n = 9–10 mice per group) following Kp inoculation were subjected to 16S rRNA gene sequencing. Pairwise community dissimilarity values between the fecal microbiota communities were visualized by Principal coordinates analysis (groups compared by AMOVA). Each data point represents an individual animal. (TIF) [file ppat.1009537.s013.tif]

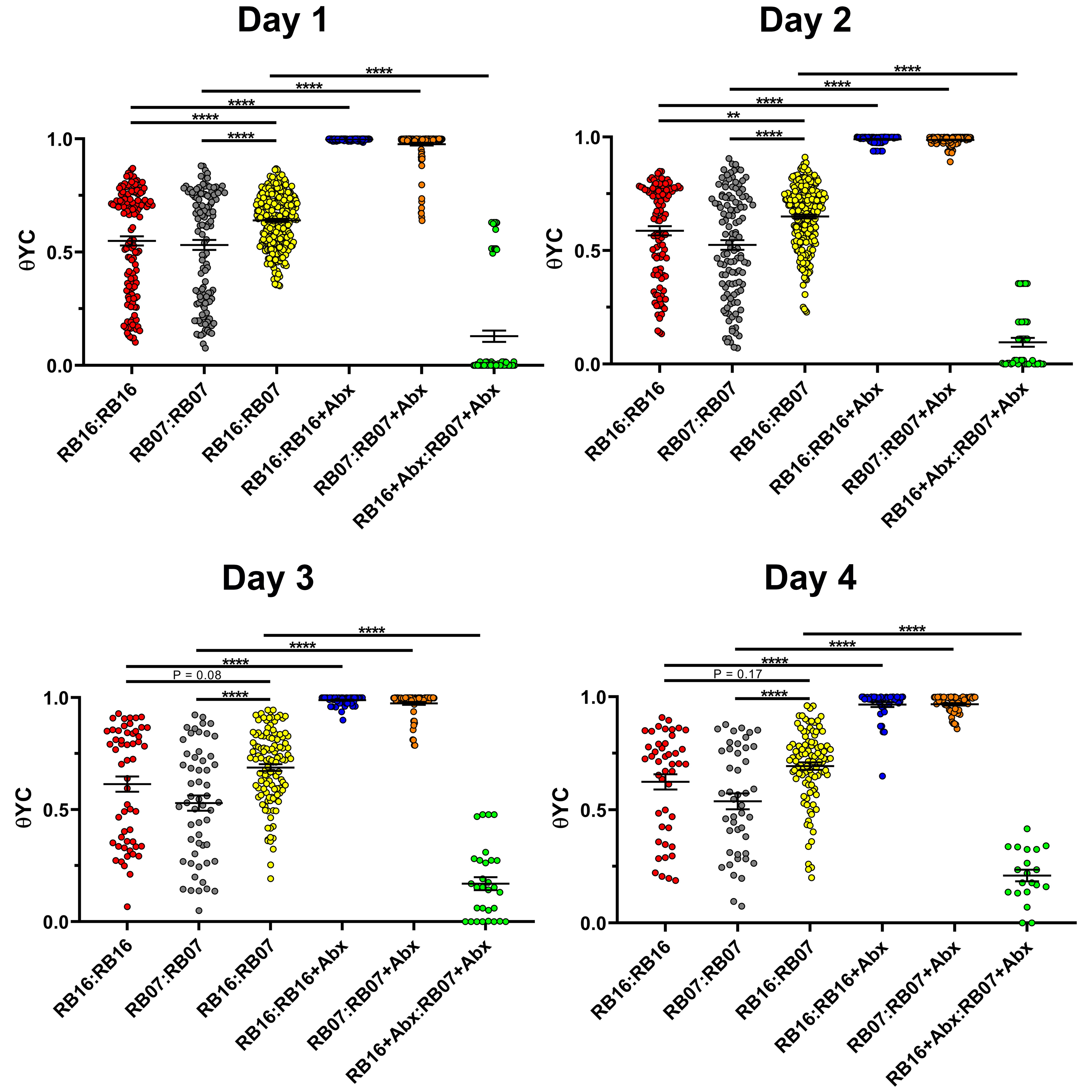

Supplement: S14 Fig — Fecal pellets collected daily from male and female C57BL6/J mice sourced from barriers RB16 and RB07 (n = 9–20 mice per group) following Kp inoculation were subjected to 16S rRNA gene sequencing. Pairwise community dissimilarity values between fecal microbiota communities were compared (**P < 0.005, ****P < 0.00005, one-way ANOVA followed by Tukey’s multiple comparisons post-hoc test). Each data point represents an individual comparison. (TIF) [file ppat.1009537.s014.tif]

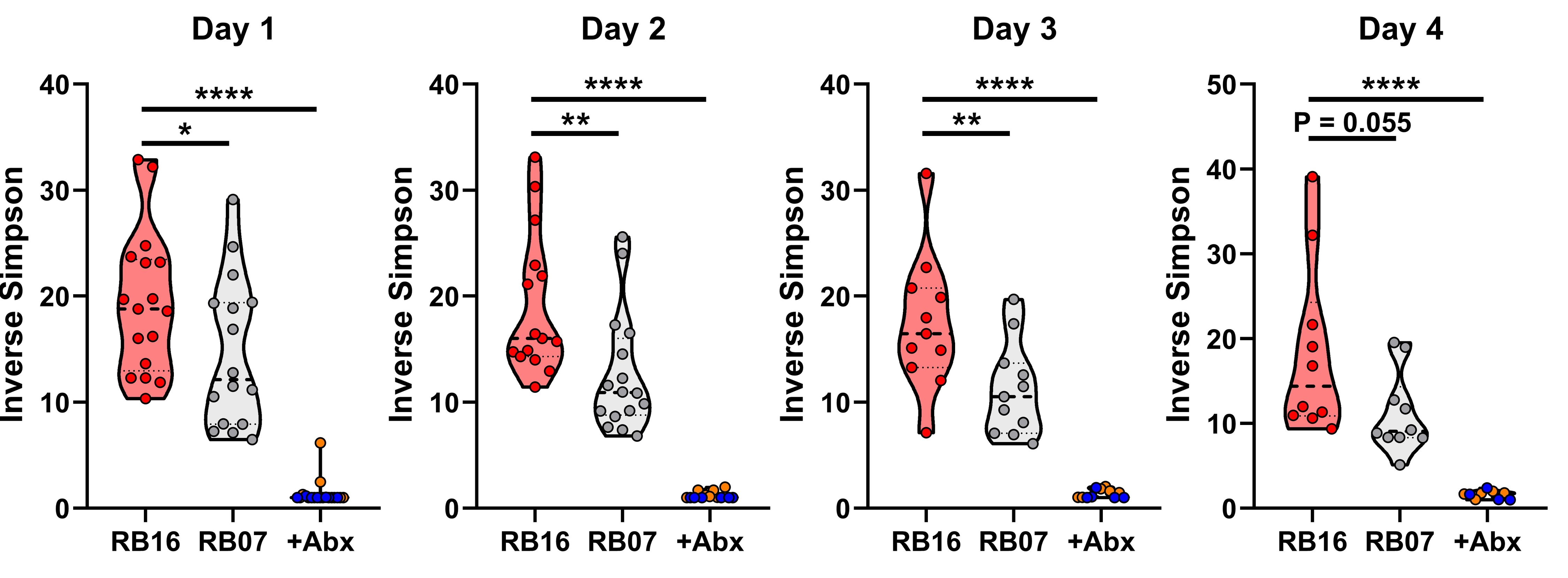

Supplement: S15 Fig — Fecal pellets collected daily from male and female C57BL6/J mice sourced from barriers RB16 and RB07 with or without three days treatment with 0.5 g/L ampicillin (n = 9–20 mice per group) following Kp inoculation were subjected to 16S rRNA gene sequencing. Diversity of the fecal microbiota was summarized by inverse Simpson index (*P < 0.05, **P < 0.005, ****P < 0.00005, one-way ANOVA followed by Tukey’s multiple comparisons post-hoc test). Each data point represents an individual animal. RB16 +Abx is displayed in blue, and RB07 +Abx is displayed in orange. (TIF) [file ppat.1009537.s015.tif]

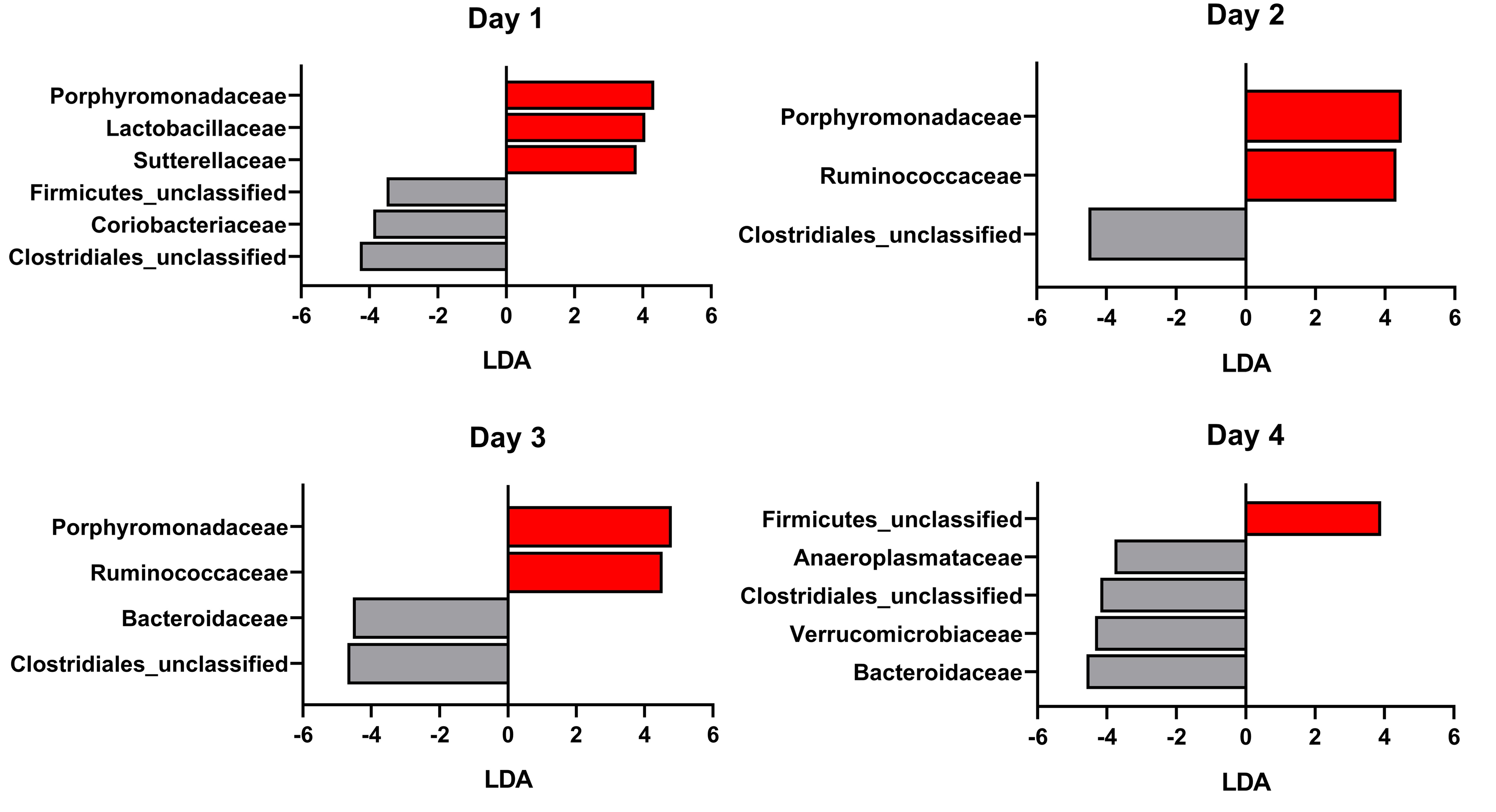

Supplement: S16 Fig — Fecal pellets collected daily from male and female C57BL6/J mice sourced from barriers RB16 and RB07 (n = 16–18 mice per group) following Kp inoculation were subjected to 16S rRNA gene sequencing. LEfSe was used to determine if specific bacterial families were differentially abundant between the fecal microbiota of RB16 and RB07 (Families with LDA ≥ 3.5 and P < 0.05 are shown). (TIF) [file ppat.1009537.s016.tif]

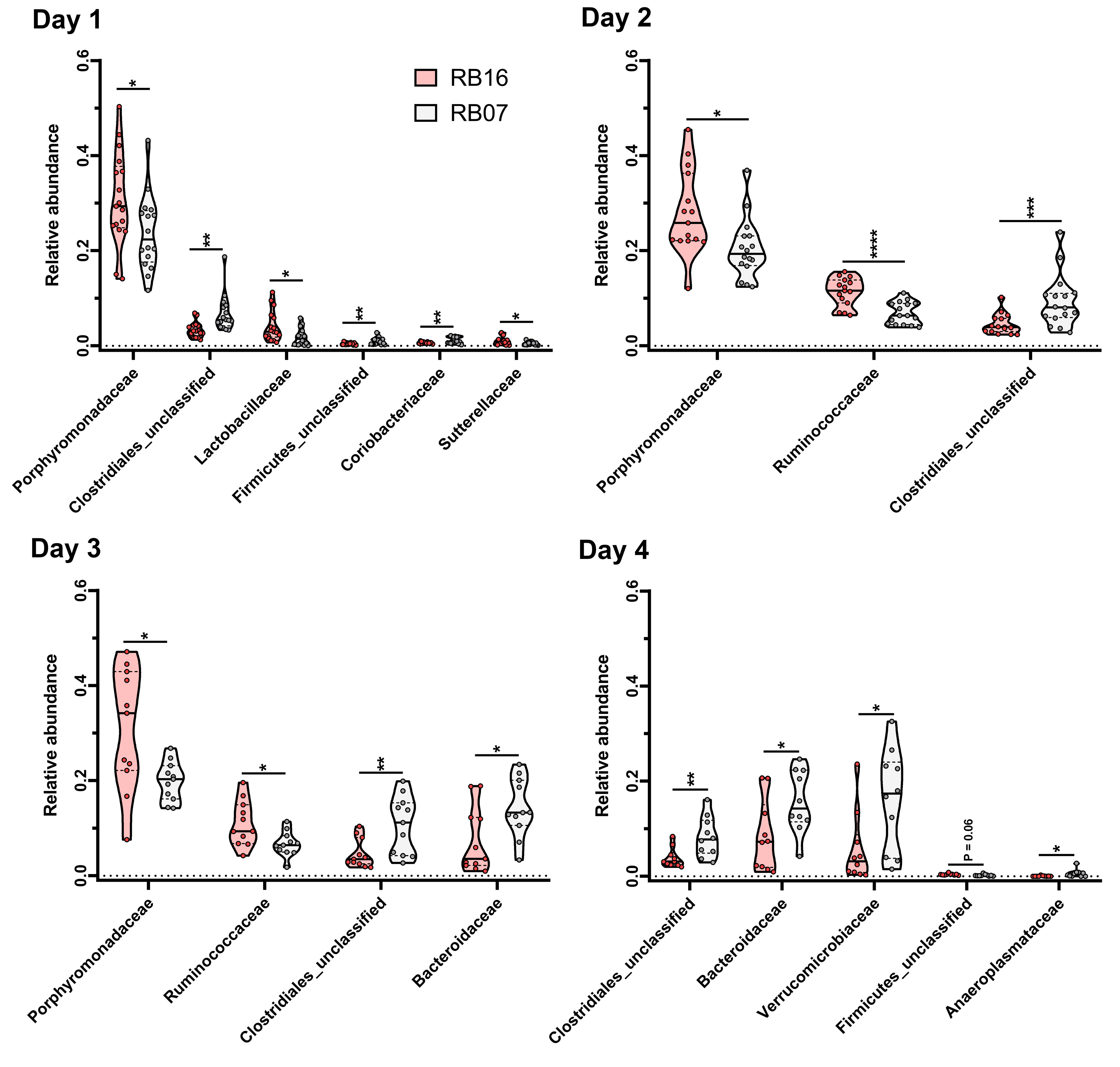

Supplement: S17 Fig — Fecal pellets collected daily from male and female C57BL6/J mice sourced from barriers RB16 and RB07 (n = 16–18 mice per group) following Kp inoculation were subjected to 16S rRNA gene sequencing. Relative abundance of specific bacterial families that were differentially abundant between the fecal microbiota of RB16 and RB07 by LEfSe are displayed (*P < 0.05, **P < 0.005, ***P < 0.0005, ****P < 0.00005, Student’s t test). (TIF) [file ppat.1009537.s017.tif]

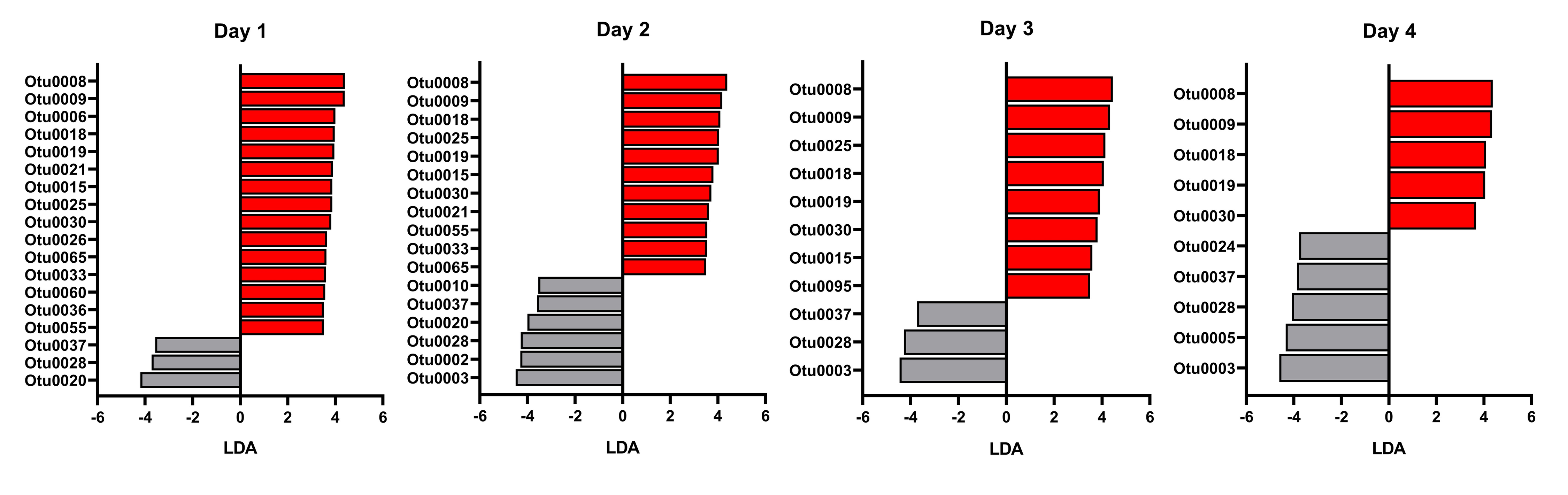

Supplement: S18 Fig — Fecal pellets collected daily from male and female C57BL6/J mice sourced from barriers RB16 and RB07 (n = 16–18 mice per group) following Kp inoculation were subjected to 16S rRNA gene sequencing. LEfSe was used to determine if specific OTUs were differentially abundant between the fecal microbiota of RB16 and RB07 (OTUs with LDA ≥ 3.5 and P < 0.05 are shown). (TIF) [file ppat.1009537.s018.tif]

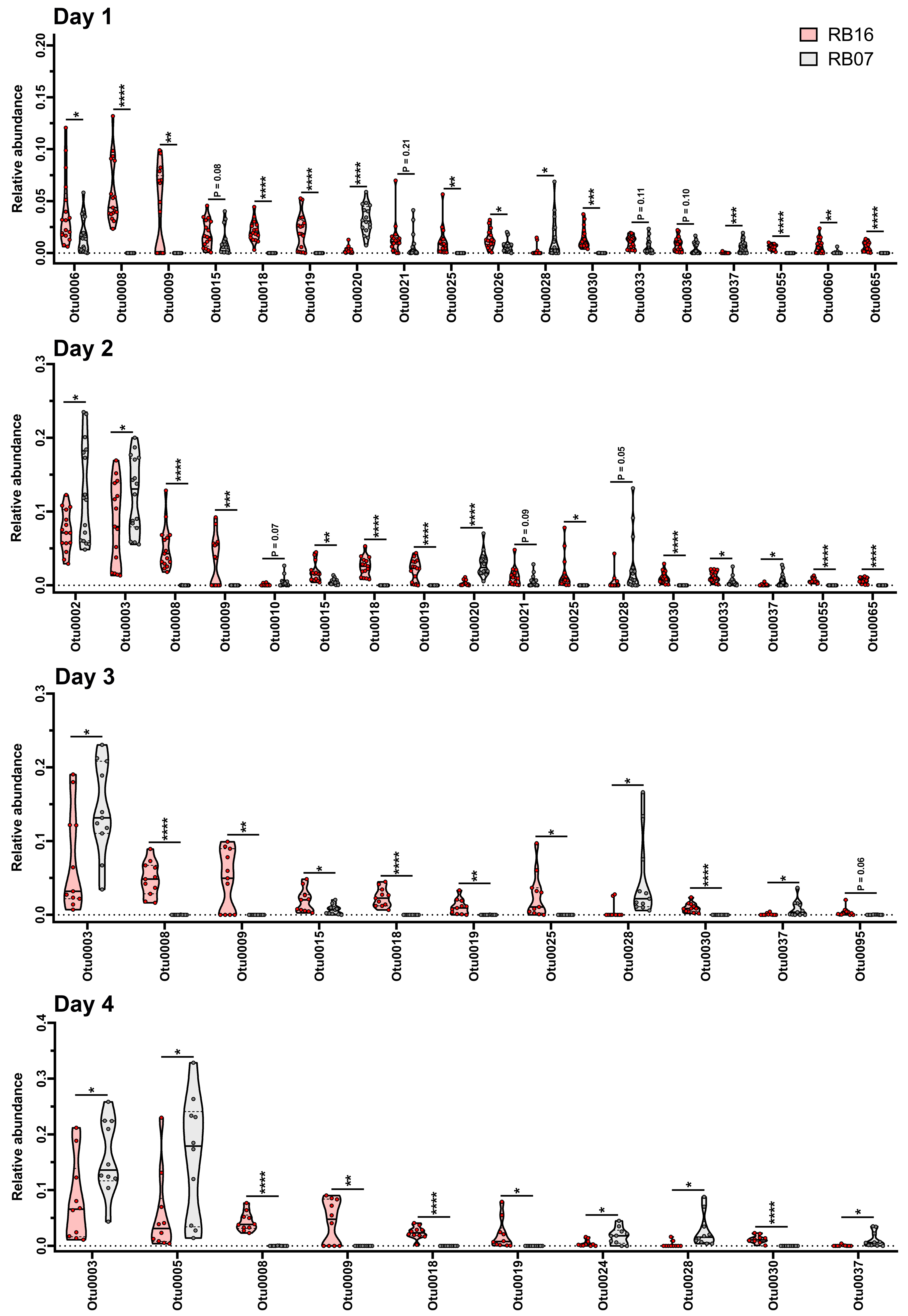

Supplement: S19 Fig — Fecal pellets collected daily from male and female C57BL6/J mice sourced from barriers RB16 and RB07 (n = 16–18 mice per group) following Kp inoculation were subjected to 16S rRNA gene sequencing. Relative abundance of specific OTUs that were differentially abundant between the fecal microbiota of RB16 and RB07 by LEfSe are displayed (*P < 0.05, **P < 0.005, ***P < 0.0005, ****P < 0.00005, Student’s t test). (TIF) [file ppat.1009537.s019.tif]

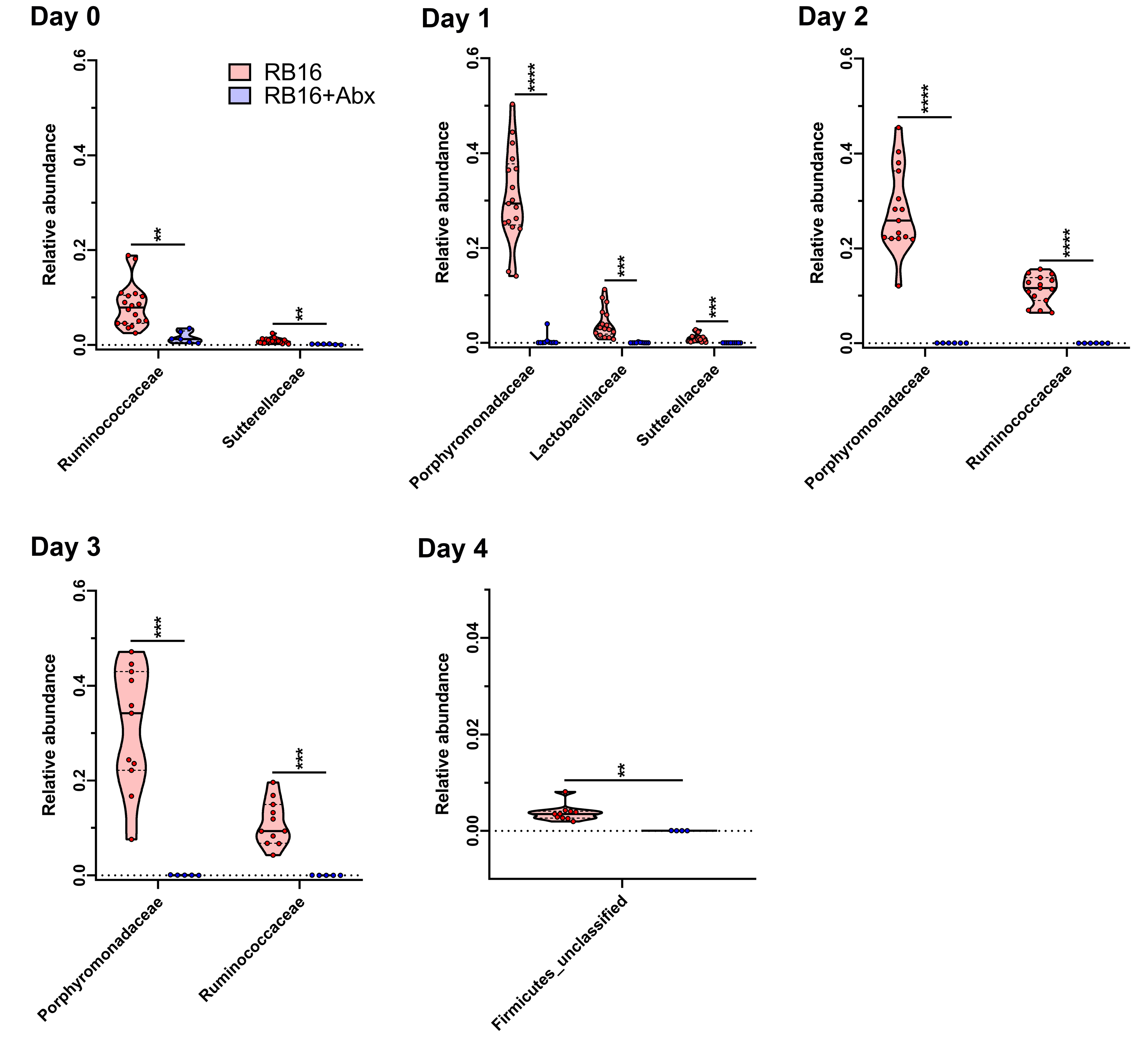

Supplement: S20 Fig — Fecal pellets collected daily from male and female C57BL6/J mice sourced from barriers RB16 and RB16+Abx (n = 10–18 mice per group) following Kp inoculation were subjected to 16S rRNA gene sequencing. Relative abundance of specific bacterial families that were differentially abundant between the fecal microbiota of RB16 and RB07 by LEfSe are displayed (**P < 0.005, ***P < 0.0005, ****P < 0.00005, Student’s t test). (TIF) [file ppat.1009537.s020.tif]

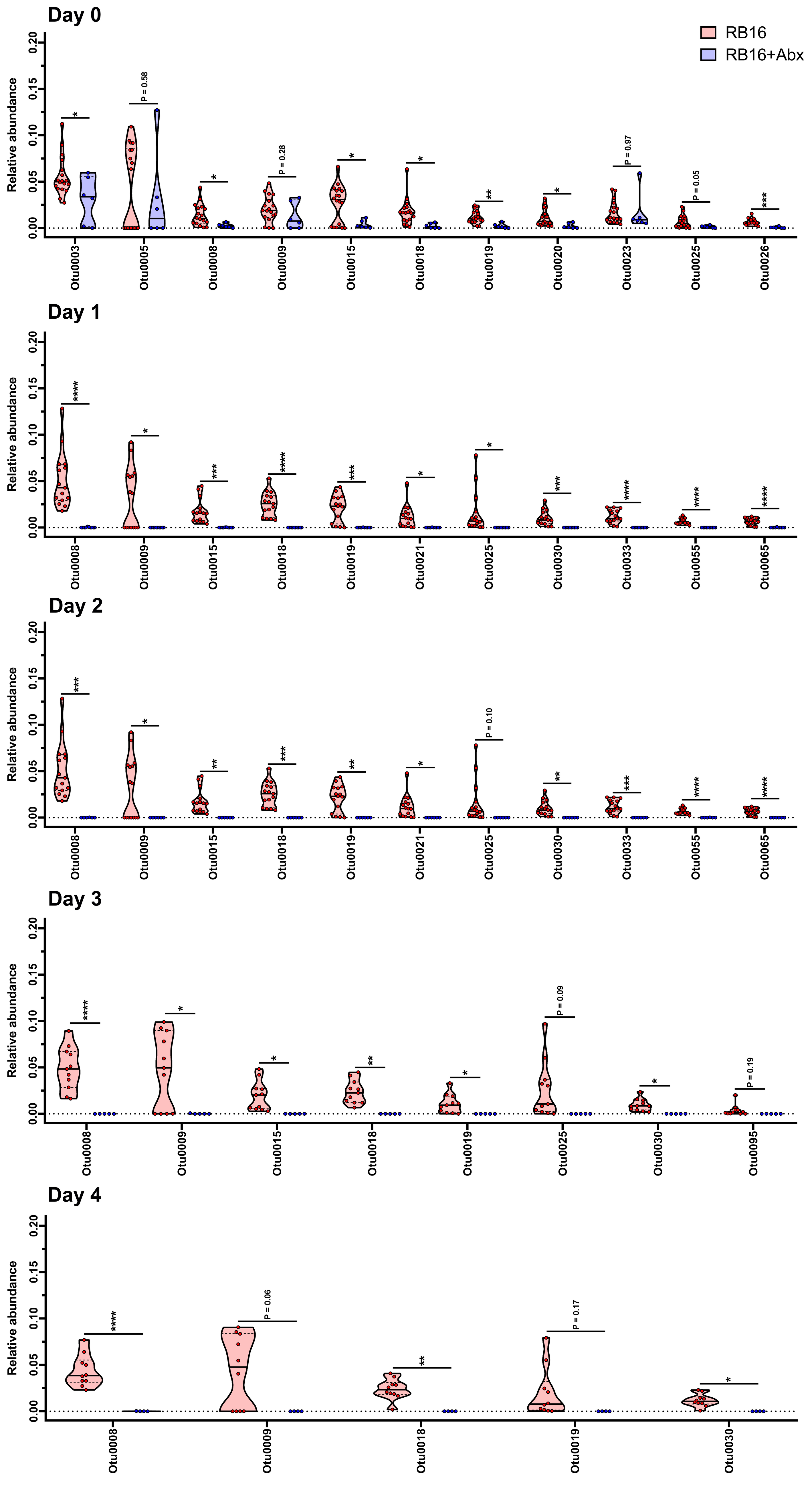

Supplement: S21 Fig — Fecal pellets collected daily from male and female C57BL6/J mice sourced from barriers RB16 and RB16+Abx (n = 10–18 mice per group) following Kp inoculation were subjected to 16S rRNA gene sequencing. Relative abundance of specific OTUs that were differentially abundant between the fecal microbiota of RB16 and RB07 by LEfSe are displayed (*P < 0.05, **P < 0.005, ***P < 0.0005, ****P < 0.00005, Student’s t test). (TIF) [file ppat.1009537.s021.tif]

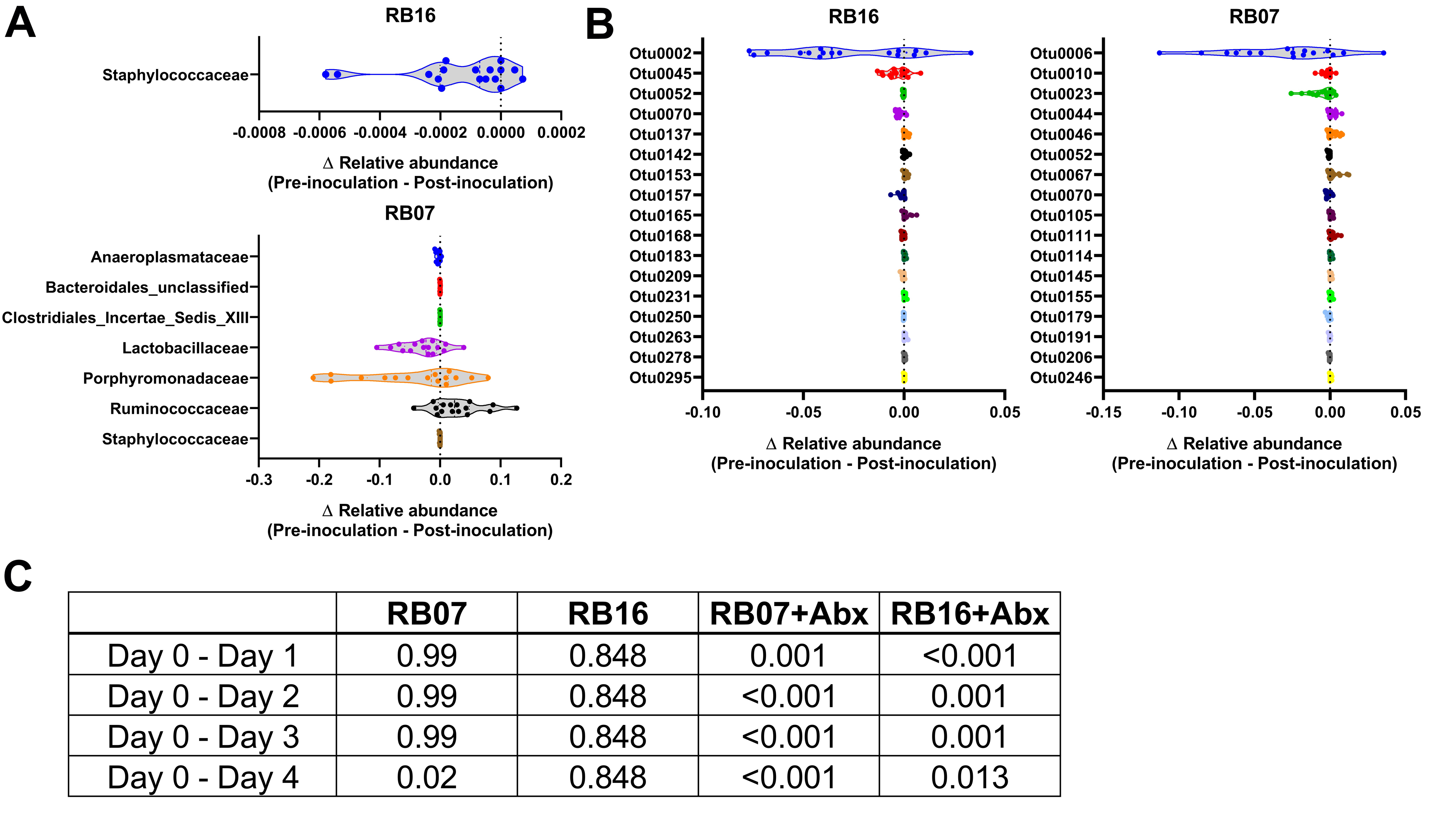

Supplement: S22 Fig — Family (A) and OTU (B) relative abundance values pre- (Day 0) and post-Kp inoculation (Day 1) were subtracted to determine the impact of Kp inoculation on the fecal microbiota communities of barriers RB16 and RB07. Only significant differential relative abundance values are displayed (median and IQR displayed, one-sample t test compared to a hypothetical value of 0). Each data point represents an individual animal. Community dissimilarity between pre- (Day 0) and post-Kp inoculation was tested by AMOVA for each day following Kp inoculation (C). Each cell contains the AMOVA P value for the indicated comparison. (TIF) [file ppat.1009537.s022.tif]

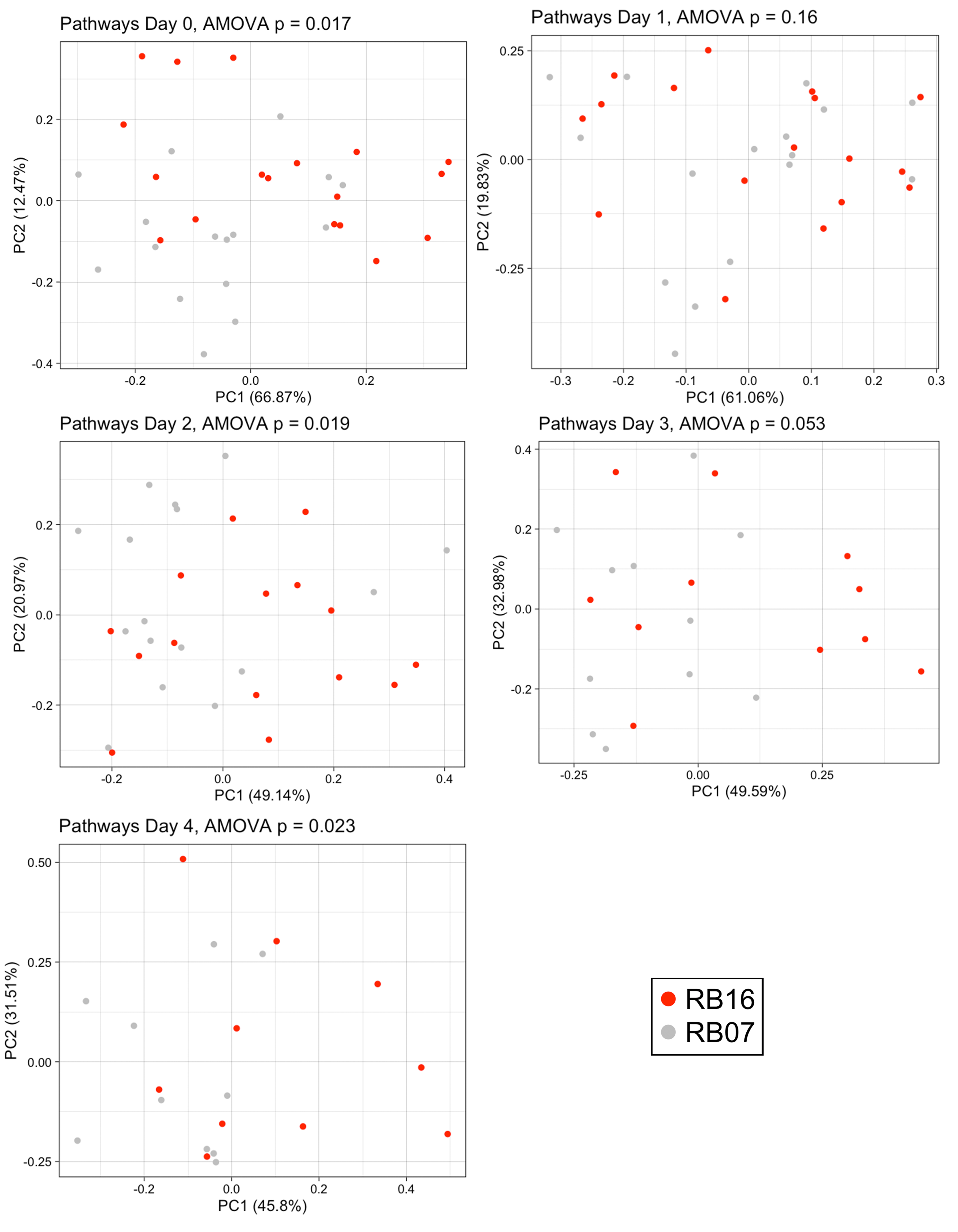

Supplement: S23 Fig — Fecal pellets collected daily from male and female C57BL6/J mice sourced from barriers RB16 and RB07 (n = 16–18 mice per group) following Kp inoculation were subjected to 16S rRNA gene sequencing and analyzed using PICRUSt2. Predicted metabolic pathway relative abundance values were visualized by Principal coordinates analysis and dissimilarity was tested by AMOVA. Each data point represents an individual animal. (TIF) [file ppat.1009537.s023.tif]

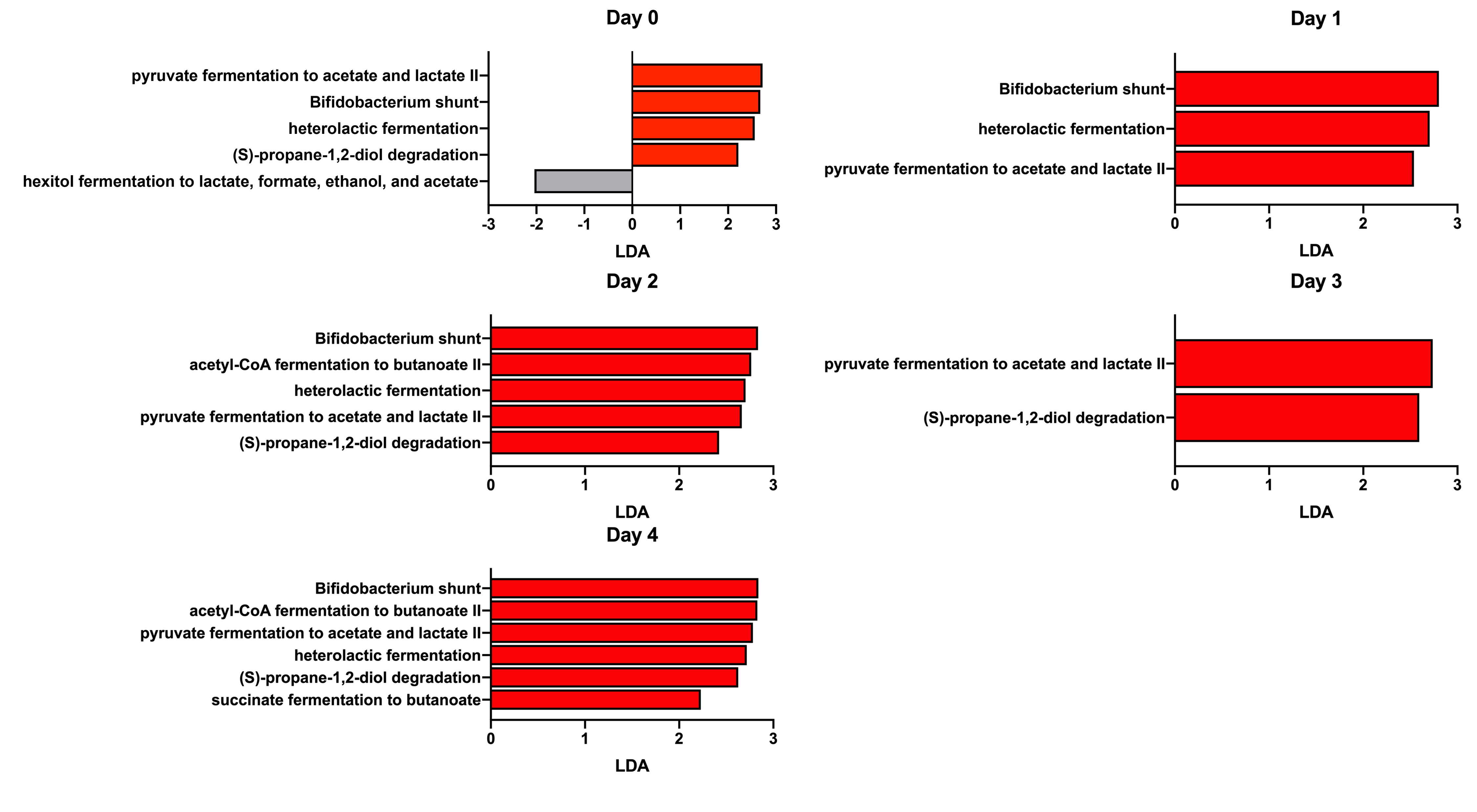

Supplement: S24 Fig — Fecal pellets collected daily from male and female C57BL6/J mice sourced from barriers RB16 and RB07 (n = 16–18 mice per group) following Kp inoculation were subjected to 16S rRNA gene sequencing and analyzed using PICRUSt2. LEfSe was used to determine if predicted fermentation to short-chain fatty acid metabolic pathways were differentially abundant between the predicted metagenome of RB16 and RB07. Note that no predicted short-chain fatty acid metabolic pathways are enriched in the gut microbiota of RB07 following day 0. (TIF) [file ppat.1009537.s024.tif]

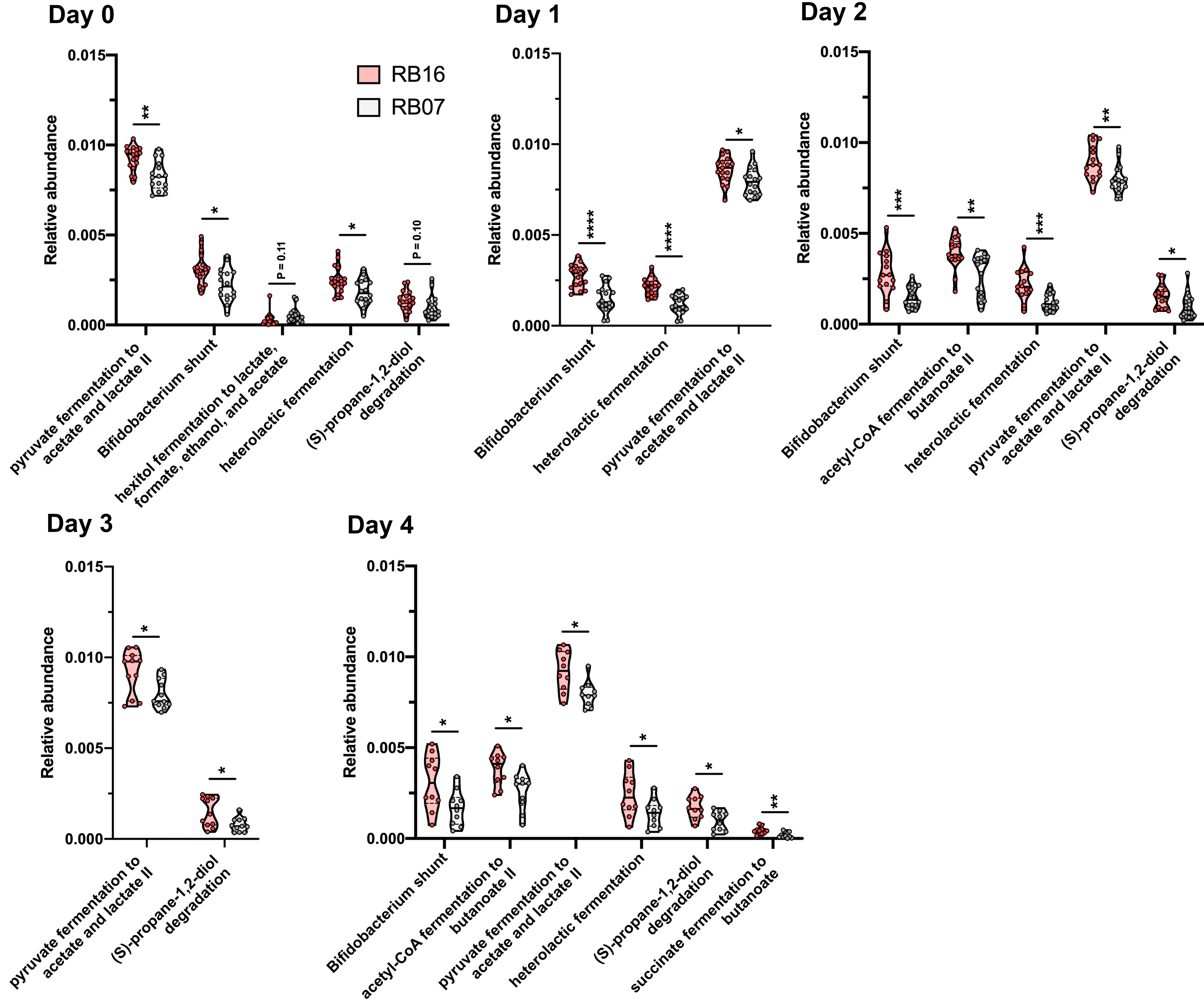

Supplement: S25 Fig — Fecal pellets collected daily from male and female C57BL6/J mice sourced from barriers RB16 and RB07 (n = 16–18 mice per group) following Kp inoculation were subjected to 16S rRNA gene sequencing and analyzed using PICRUSt2. Relative abundance of specific PICRUSt2 predicted fermentation to short-chain fatty acid pathways that were differentially abundant between RB16 and RB07 by LEfSe are displayed (*P < 0.05, **P < 0.005, ***P < 0.0005, ****P < 0.00005, Student’s t test). (TIF) [file ppat.1009537.s025.tif]

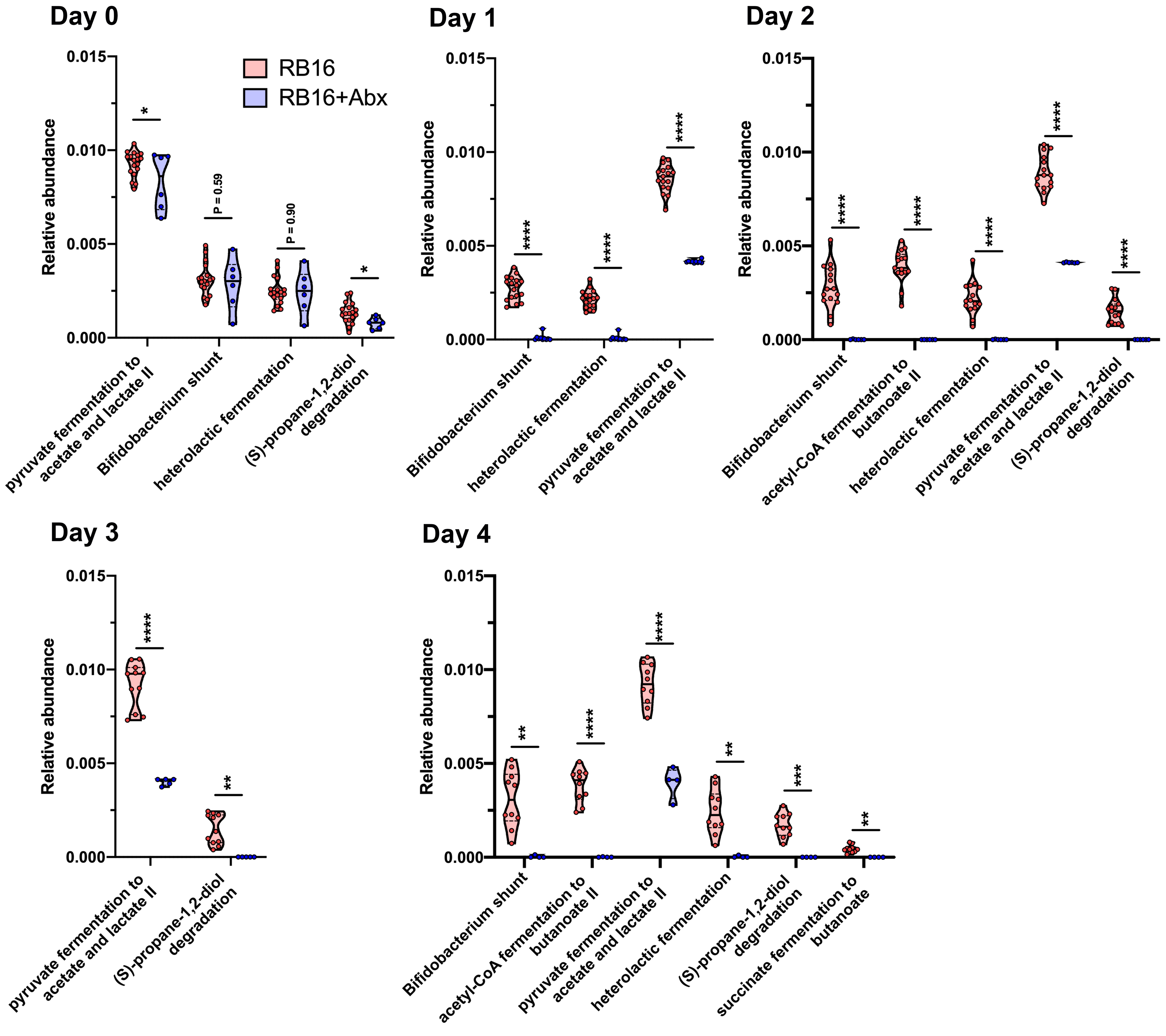

Supplement: S26 Fig — Fecal pellets collected daily from male and female C57BL6/J mice sourced from barriers RB16 with or without three days treatment with 0.5 g/L ampicillin (n = 10–18 mice per group) following Kp inoculation were subjected to 16S rRNA gene sequencing and analyzed using PICRUSt2. Relative abundance of specific PICRUSt2 predicted fermentation to short-chain fatty acid pathways that were differentially abundant between RB16 and RB07 by LEfSe are displayed (*P < 0.05, **P < 0.005, ***P < 0.0005, ****P < 0.00005, Student’s t test). (TIF) [file ppat.1009537.s026.tif]

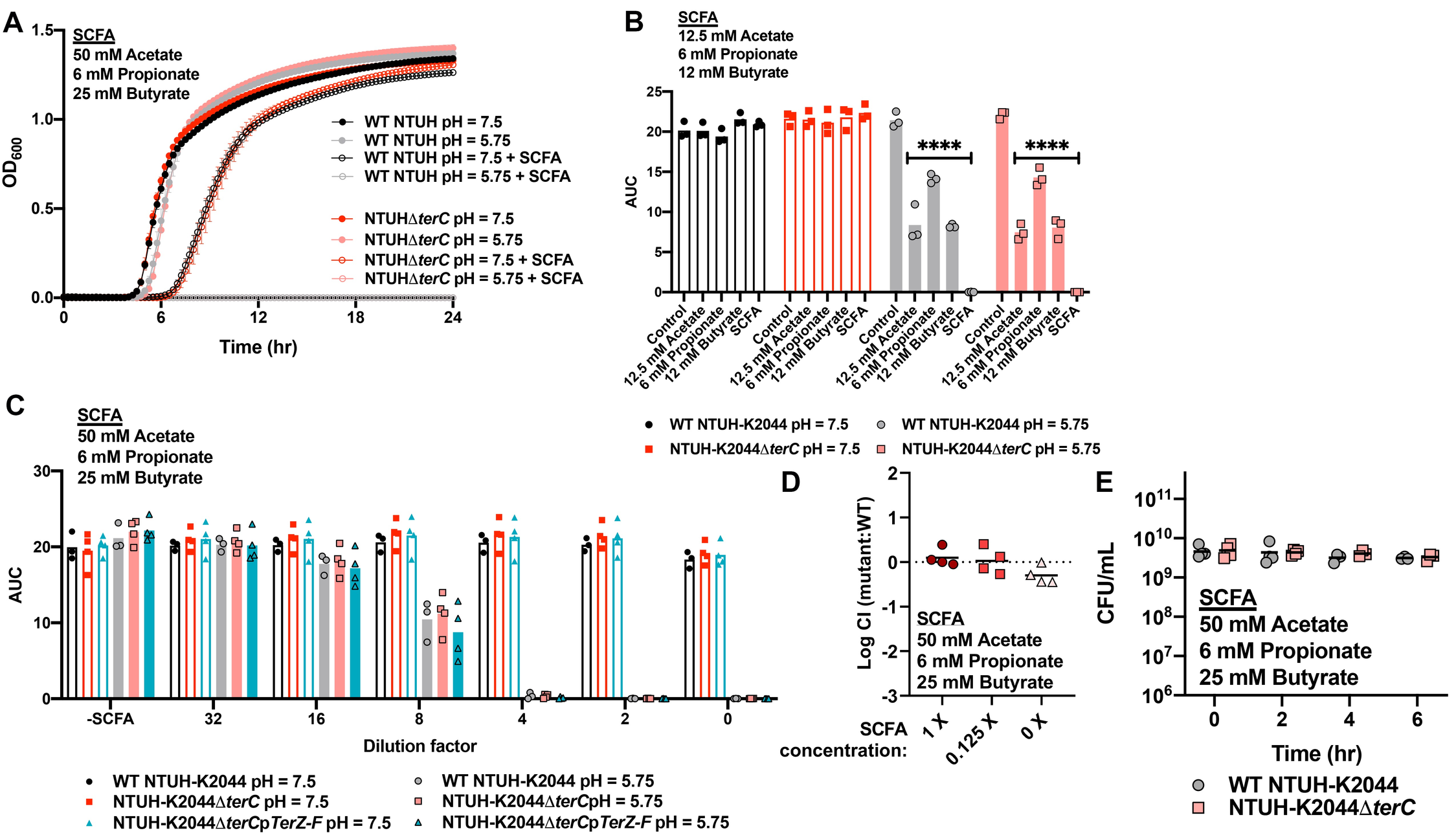

Supplement: S27 Fig — WT NTUH-K2044 and the isogenic ΔterC mutant (clone Kp2257) were grown in neutral (pH = 7.5) or acidic (pH = 5.75) conditions in the absence or presence of various SCFAs in combination or individually (A-B). Area under the curve (AUC) was calculated from growth curves (B, ****P < 0.00005 compared to no SCFA control, Tukey’s multiple comparison test following Two-way ANOVA, mean displayed ± SEM). WT NTUH-K2044, the isogenic ΔterC mutant, and the complement strain were grown in neutral (pH = 7.5) or acidic (pH = 5.75) conditions in the presence of increasing dilutions of SCFAs, and AUC was calculated (C). To assess inter-strain antagonism in a competitive growth assay, WT NTUH-K2044 and the isogenic ΔterC mutant were mixed 1:1 at an OD600 = 0.01 and exposed to indicated dilutions of SCFAs under acidic conditions (pH = 5.75) for 24 hours. After 24 hours, bacteria were dilution plated on selective media and log competitive indices (mutant:WT) were calculated (D). Stationary phase WT NTUH-K2044 and the isogenic ΔterC mutant were exposed to SCFAs under acidic conditions (pH = 5.75) to assess bacterial killing (E). (TIF) [file ppat.1009537.s027.tif]

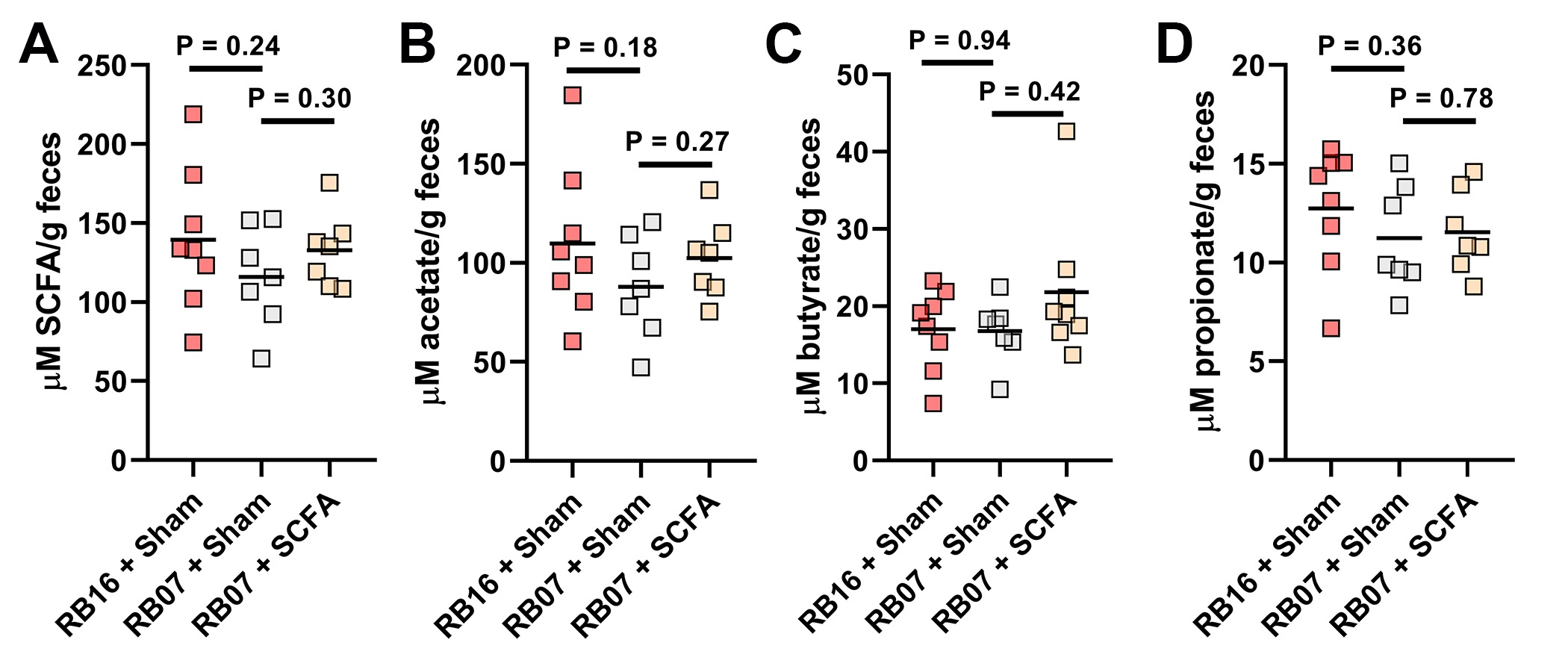

Supplement: S28 Fig — Male and female C57BL6/J mice sourced from barriers RB16 and RB07 were treated with a SCFA cocktail or regular drinking water (sham) for 7 days. Total SCFAs (A), acetate (B), butyrate (C), and propionate (D) were quantified from fecal pellets (mean displayed, LSD test following one-way ANOVA). (TIF) [file ppat.1009537.s028.tif]

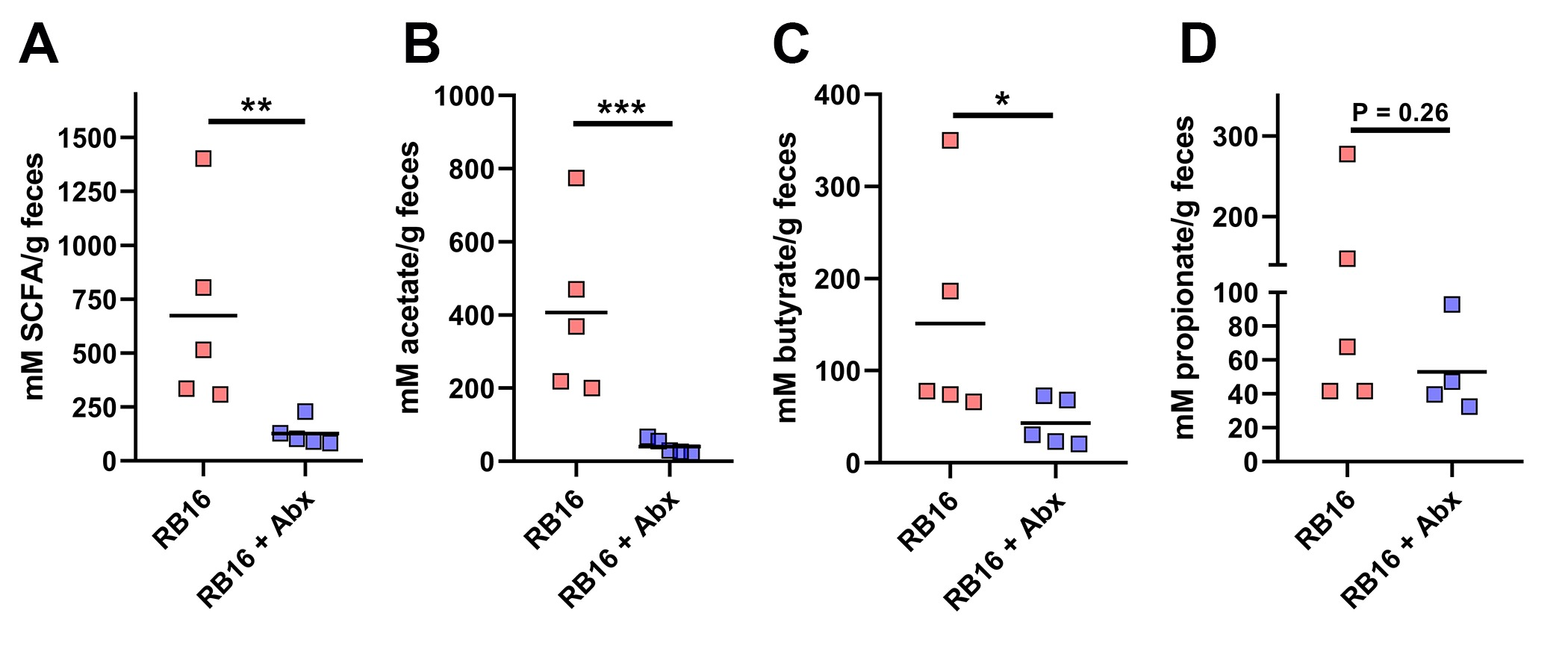

Supplement: S29 Fig — Male and female C57BL6/J mice sourced from barrier RB16 were treated with or without antibiotics = for 3 days. Total SCFAs (A), acetate (B), butyrate (C), and propionate (D) were quantified from fecal pellets (mean displayed, *P < 0.05, **P < 0.005, ***P < 0.0005, Student’s t test). (TIF) [file ppat.1009537.s029.tif]
